# Relevance of DNA repair gene polymorphisms to gastric cancer risk and phenotype

**DOI:** 10.18632/oncotarget.16261

**Published:** 2017-03-16

**Authors:** Patricia Carrera-Lasfuentes, Angel Lanas, Luis Bujanda, Mark Strunk, Enrique Quintero, Santos Santolaria, Rafael Benito, Federico Sopeña, Elena Piazuelo, Concha Thomson, Angeles Pérez-Aisa, David Nicolás-Pérez, Elizabeth Hijona, Jesús Espinel, Rafael Campo, Marisa Manzano, Fernando Geijo, María Pellise, Manuel Zaballa, Ferrán González-Huix, Jorge Espinós, Llúcia Titó, Luis Barranco, Mauro D'Amato, María Asunción García-González

**Affiliations:** ^1^ CIBER de Enfermedades Hepáticas y Digestivas (CIBERehd), Madrid, Spain; ^2^ Instituto de Investigación Sanitaria Aragón (IIS Aragón), Zaragoza, Spain; ^3^ Department of Gastroenterology, Hospital Clínico Universitario Lozano Blesa, Zaragoza, Spain; ^4^ Faculty of Medicine, Universidad de Zaragoza, Zaragoza, Spain; ^5^ Department of Gastroenterology, Hospital Donostia/Instituto Biodonostia, Universidad del País Vasco (UPV/EHU), San Sebastián, Spain; ^6^ Instituto Aragonés de Ciencias de la Salud (IACS), Zaragoza, Spain; ^7^ Department of Gastroenterology, Hospital Universitario de Canarias, Instituto Universitario de Tecnologías Biomédicas (ITB), Centro de Investigación Biomédica de Canarias (CIBICAN), Tenerife, Spain; ^8^ Department of Gastroenterology, Hospital San Jorge, Huesca, Spain; ^9^ Faculty of Medicine and Department of Microbiology, Hospital Clínico Universitario, Zaragoza, Spain; ^10^ Department of Gastroenterology, Hospital Obispo Polanco, Teruel, Spain; ^11^ Department of Gastroenterology, Hospital del Sol, Marbella, Spain; ^12^ Department of Gastroenterology, Complejo Hospitalario, León, Spain; ^13^ Department of Gastroenterology, Hospital Parc Tauli, Sabadell, Spain; ^14^ Department of Gastroenterology, Hospital 12 de Octubre, Madrid, Spain; ^15^ Department of Gastroenterology, Hospital Clínico Universitario, Salamanca, Spain; ^16^ Department of Gastroenterology, Hospital Clinic I Provincial, Institut d Investigacions Biomèdiques August Pi i Sunyer (IDIBAPS), Universidad de Barcelona, Barcelona, Spain; ^17^ Department of Gastroenterology, Hospital de Cruces, Barakaldo, Spain; ^18^ Department of Gastroenterology, Hospital Josep Trueta, Girona, Spain; ^19^ Department of Gastroenterology, Mutua de Tarrasa, Spain; ^20^ Department of Gastroenterology, Hospital de Mataró, Mataró, Spain; ^21^ Department of Gastroenterology, Hospital del Mar, Barcelona, Spain; ^22^ BioDonostia Health Research Institute, IKERBASQUE, Basque Foundation for Science, San Sebastián, Spain

**Keywords:** gastric cancer, Helicobacter pylori, polymorphism, DNA repair

## Abstract

Variations in DNA repair genes have been reported as key factors in gastric cancer (GC) susceptibility but results among studies are inconsistent. We aimed to assess the relevance of DNA repair gene polymorphisms and environmental factors to GC risk and phenotype in a Caucasian population in Spain. Genomic DNA from 603 patients with primary GC and 603 healthy controls was typed for 123 single nucleotide polymorphisms in DNA repair genes using the Illumina platform. *Helicobacter pylori* infection with CagA strains (odds ratio (OR): 1.99; 95% confidence interval (CI): 1.55–2.54), tobacco smoking (OR: 1.77; 95% CI: 1.22–2.57), and family history of GC (OR: 2.87; 95% CI: 1.85–4.45) were identified as independent risk factors for GC. By contrast, the *TP53* rs9894946A (OR: 0.73; 95% CI: 0.56–0.96), *TP53* rs1042522C (OR: 0.76; 95% CI: 0.56–0.96), and *BRIP1* rs4986764T (OR: 0.55; 95% CI: 0.38–0.78) variants were associated with lower GC risk. Significant associations with specific anatomopathological GC subtypes were also observed, most notably in the *ERCC4* gene with the rs1799801C, rs2238463G, and rs3136038T variants being inversely associated with cardia GC risk. Moreover, the *XRCC3* rs861528 allele A was significantly increased in the patient subgroup with diffuse GC (OR: 1.75; 95% CI: 1.30–2.37). Our data show that specific *TP53*, *BRIP1*, *ERCC4*, and *XRCC3* polymorphisms are relevant in susceptibility to GC risk and specific subtypes in Caucasians.

## INTRODUCTION

Gastric cancer (GC) represents the fifth most common neoplasia and the third leading cause of cancer–related death worldwide [1]. Despite continuous advances in diagnosis and treatment, most patients with GC present with late–stage disease and poor prognosis. Therefore, it is not surprising that detection of potential risk factors is regarded as the most effective option to prevent and reduce the incidence of GC.

GC is a heterogeneous disease that shows distinct clinical, epidemiological, and molecular features among tumors arising from the proximal (cardia) or distal (non–cardia) stomach, and among intestinal and diffuse histological subtypes [2, 3]. These differences in phenotype seem to be determined by complex interactions between environmental and host genetic factors. Among them, *Helicobacter pylori* (*H. pylori*) infection has been identified as the single most common cause of GC [4]. This organism, which colonizes over half of the world's population, first induces a chronic superficial gastritis in virtually all infected people, initiating a process that in certain individuals may lead to GC [5]. Several studies have found that bacterial virulence determinants, such as the presence of the CagA pathogenicity island, are associated with a higher risk of GC development [6, 7]. However, why only a minority (< 1%) of infected individuals develops gastric malignancy remains a matter of speculation, suggesting that factors other than bacterial infection alone are involved in the carcinogenesis process.

Additional host genetic factors are also likely to contribute in gastric carcinogenesis. In this respect, genetic susceptibility may be critical in several relevant processes such as mucosal protection, immune response, carcinogen detoxification, antioxidant protection, cell proliferation, and DNA repair [8]. Concerning the latter, a complex system involving multiple enzymes and pathways plays a crucial role in maintaining genome homeostasis in the face of exogenous and endogenous agents and general DNA replication errors. In this context, five major DNA repair pathways have been described: i) base excision repair (BER) fixes simple base modifications (oxidized/reduced bases) and DNA single-strand breaks caused by ionizing radiation, alkylating agents, and oxidative stress [9]; ii) nucleotide excision repair (NER) repairs the damage caused by bulky adducts produced by ultraviolet light and a wide array of chemical agents [10]; iii) mismatch repair (MMR) removes base–base mismatches, small loops, and insertion/deletion mispairs occurring during DNA replication and recombination [11]; iv) double–strand break repair (DSBR) evolves two main mechanisms, non–homologous end joining and homologous recombination repair, whichrepair the most severe type of DNA damage [12] and v) direct reversion repair (DR) corrects methylated bases, namely *O*^6^-methylguanine (*O*^6^MeG), induced by alkylating agents [13].

DNA repair genes harbor functional polymorphic regions that have been reported to influence the host capacity to repair damaged DNA. Therefore, it is plausible that individuals carrying deficient DNA repair alleles will be at greater risk of presenting mutations that can alter genome integrity and stability leading to cancer development. Common single nucleotide polymorphisms (SNPs) in DNA repair genes have been identified as potential risk factors for a wide array of cancers, including lung [14], ovarian [15], prostate [16], and breast cancer [17]. However, the data are not conclusive concerning the relationship between DNA repair variants and GC; whereas some studies report a link with GC risk [18, 19] or anatomopathological subtypes [20, 21], other studies have failed to confirm any associations [22, 23].

Trying to address this issue, we sought to evaluate the influence of 123 selected DNA repair gene polymorphisms and environmental factors (*H. pylori* infection and smoking habits) to GC risk and phenotype in a Caucasian population in Spain. Because GC shows marked heterogeneity in histology and anatomic involvement, we aimed also to analyze the role of both environmental and host genetic factors with regard to the site and histological type of the tumor.

## RESULTS

### Clinical and demographic characteristics of GC patients and healthy controls

The clinical and demographic characteristics of GC patients and healthy controls (HCs) are shown in Table 1. Of the 603 patients with GC, 117 (19.4%) were classified as cardia and 486 (80.6%) as non–cardia GC cases. According to Lauren's classification [3], non–cardia gastric adenocarcinomas were of intestinal histotype in 250 patients (51.4%), diffuse histotype in 178 patients (36.6%), and mixed or undetermined type in 58 patients (11.9%). Among the evaluated features, infection with *H. pylori* and *CagA+* strains was significantly associated with a higher risk of GC (*H. pylori*, odds ratio (OR): 1.38; 95% confidence interval (CI): 1.09–1.76; *P* = 0.008; *CagA*+ strains, OR: 1.95; 95% CI: 1.54–2.46; *P* < 0.0001). However, no differences in the prevalence of *VacA*+ strains were observed between GC patients and controls (40.1% *vs*. 39.3%). When considering the anatomic location of the tumor, infection with *CagA*+ strains was specifically associated with a higher risk of developing non–cardia GC (62.6% *vs*. 41.3%; OR: 2.32; 95% CI: 1.80–2.98; *P* < 0.0001) (Supplementary Table 1). Of interest, no association between *H. pylori* infection and cardia GC was found. Among non–cardia GC patients, *H. pylori* infection with *CagA*+ strains was significantly more frequent in both diffuse and intestinal tumor subtypes compared with controls (OR: 3.02; 95% CI: 2.08–4.37; *P* < 0.0001 *vs*. HC; and OR: 2.25; 95% CI: 1.65–3.07; *P* < 0.0001 *vs*. HC, respectively).

**Table 1:** Demographic and clinical characteristics of healthy controls and gastric cancer patients

	Healthy Controls *n* = 603	GC patients *n* = 603	ORs (95% CI)	*P* Value
Age yrs ± SD (range)	70.17 ± 12.32	70.32 ± 12.27		0.97
	(30–96)	(29–96)		
Gender	413 M (68.5%)	413 M (68.5%)		
	190 F (31.5%)	190 F (31.5%)	1 (0.78–1.27)	1.04
^a^Cigarette smoking				
Never	311/585 (53.2%)	291/582 (50%)	Reference	
Former	184/585 (31.4%)	162/582 (27.8 %)	0.94 (0.72–1.23)	0.68
Current smoker	90/585 (15.4%)	129/582 (22.2%)	1.53 (1.12–2.09)	0.009
^b^*H. pylori* +	364 (60.4%)	377/556 (67.8%)	1.38 (1.09–1.76)	0.008
CagA+	253 (41.9%)	325/556 (58.4%)	1.95 (1.54–2.46)	< 0.0001
VacA+	237 (39.3%)	223/556 (40.1%)	1.03 (0.82–1.31)	0.81
^c^Family history of GC	33/539 (6.1%)	88/534 (16.5%)	3.02 (1.99–4.60)	< 0.0001
Neoplasia location				
Cardia		117 (19.4%)		
Non–cardia		486 (80.6%)		
Lauren's classification				
Intestinal		250 (51.4%)		
Diffuse		178 (36.6%)		
Mixed or undetermined		58 (11.9%)		
^d^TNM stage				
Stage I		80 (13.3%)		
Stage II		79 (13.1%)		
Stage III		131 (21.7%)		
Stage IV		264 (43.8%)		
Could not be assessed		49 (8.1%)		
Surgical treatment		410 (68%)		
Chemotherapy		225 (37.3%)		
Radiotherapy		105 (17.4%)		

GC, gastric cancer; n, number of individuals; OR, odds ratio; CI, confidence interval; SD, standard deviation; M, male; F, female.

^a^Information was available for 585 HC and 582 GC patients.

^b^*Helicobacter pylori* infection and CagA/VacA antibody data obtained by western blot analysis in serum samples. Information was available for 603 HC and 556 GC patients.

^c^Information was available for 539 HC and 534 GC patients.

^d^Clinical tumor stages according to the International Union Against Cancer (UICC) criteria. In 49 GC patients tumor stage could not be assessed.

In addition to *H. pylori* infection, active smoking (OR: 1.53; 95% CI: 1.12–2.09) and family history of GC (OR: 3.02; 95%: 1.99–4.60) were also found to be risk factors for the development of GC (Table 1). Stratified analysis by tumor location showed that smoking habit was strongly associated with cardia GC (OR: 2.93; 95% CI: 1.68–5.11; *P* = 0.0002) whereas a positive family history of GC was specifically associated with a higher risk of non–cardia GC (OR: 3.29; 95% CI: 2.14–5.06; *P* < 0.0001), (Supplementary Table 1).

### Genotyping

#### Single marker analysis

Of the 123 SNPs initially evaluated in our study, 108 SNPs were successfully genotyped in 1206 subjects (603 GC patients and 603 HCs) and available for analysis. Supplementary Table 2 summarizes the genotype distribution of each polymorphism in GC patients and controls according to the location and histological type of the tumor. Genotype frequencies did not deviate significantly from those expected under Hardy–Weinberg equilibrium in the control group. Moreover, no evidence of genetic heterogeneity among study participants, either patients or controls, was observed (data not shown).

Fourteen SNPs (*TP53* rs1042522, *RAD52* rs11226, *ERCC5* rs17655, *POLG* rs176641, *BRCA2* rs1801406, *LIG3* rs2074522, *XPC* rs2228000, *ERCC4* rs2238463, *MGMT* rs2308321, *MSH3* rs26779, *ERCC4* rs3136038, *BRIP1* rs4986764, *XRCC3* rs861528, and *TP53* rs9894946) revealed significant associations with GC risk (*P* < 0.05) in at least one of the four genetic models evaluated in the analysis (Supplementary Table 3). However, after false discovery rate (FDR) multiple test correction, only four SNPs in the *TP53* (rs1042522G>C, rs9894946G>A), *LIG3* (rs2074522G>A), and *BRIP1* (rs4986764C>T) genes retained significance (Table 2). Thus, the *TP53* rs1042522C and *TP53* rs9894946A variants were inversely associated with GC risk (dominant models, OR: 0.67; 95% CI: 0.53–0.85; and OR: 0.69; 95% CI: 0.53–0.90, respectively). The *BRIP1* rs4986764T variant also showed a protective effect (recessive model, OR: 0.59; 95% CI: 0.42–0.83). By contrast, the rare allele A of *LIG3* rs2074522 was associated with a higher risk of developing the disease (recessive model, OR: 0.59; 95% CI: 1.58–31.5).

**Table 2:** Association of DNA repair gene polymorphisms with GC risk

			HC Genotype			GC Genotype			Dominant model		Recessive model		Log-Additive model		
Gen db SNP ID		A/a	*H. pylori*	AA	Aa	aa	AA	Aa	aa	OR (95% CI)^a^ *P*-value	FDR^b^	OR (95% CI)^a^ *P*-value	FDR^b^	OR (95% CI)^a^ *P*-value	FDR^b^
*TP53* rs1042522		G/C	Overall n	314	247	40	372	192	39	0.67 (0.53–0.85) **0.001**	**0.012**	1.05 (0.66–1.67) 0.832	0.896	0.78 (0.65–0.94) **0.009**	0.068
			*H. pylori +*	180	159	23	241	115	21	0.55 (0.41–0.74) **0.0001**	**0.002**	0.85 (0.46–1.58) 0.619	0.734	0.65 (0.51–0.83) **0.001**	**0.013**
			*H. pylori –*	134	88	17	103	59	17	0.95 (0.64–1.41) 0.803	0.902	1.35 (0.66–2.74) 0.413	0.912	1.02 (0.76–1.39) 0.875	0.983
*TP53* rs9894946		G/A	Overall n	409	165	18	452	138	6	0.69 (0.53–0.90) **0.006**	**0.013**	0.23 (0.08–0.68) **0.002**	**0.014**	0.66 (0.52–0.85) **0.001**	**0.017**
			*H. pylori +*	249	98	11	288	82	3	0.65 (0.46–0.91) **0.019**	0.095	0.27 (0.07–0.97) **0.021**	0.143	0.64 (0.47–0.87) **0.004**	0.053
			*H. pylori –*	160	67	7	132	44	1	0.73 (0.47–1.14) 0.168	0.832	0.19 (0.02–1.57) 0.065	0.674	0.70 (0.47–1.05) 0.085	0.659
*LIG3* rs2074522		G/A	Overall n	509	89	2	496	94	13	1.22 (0.89–1.67) 0.217	0.268	7.05 (1.58–31.5) **0.001**	**0.014**	1.32 (0.99–1.75) 0.055	0.080
			*H. pylori +*	306	54	2	309	59	9	1.23 (0.83–1.81) 0.302	0.393	4.3 (0.92–20.28) **0.035**	0.143	1.30 (0.92–1.85) 0.135	0.201
			*H. pylori –*	203	35	–	148	27	4	1.16 (0.68–1.97) 0.591	0.902	NA	NA	1.33 (0.80–2.11) 0.296	0.659
*BRIP* rs4986764		C/T	Overall n	224	270	107	232	297	69	0.94 (0.74–1.20) 0.633	0.724	0.59 (0.42–0.83) **0.002**	**0.015**	0.85 (0.72–1.00) 0.055	0.075
			*H. pylori +*	134	166	64	145	188	42	0.93 (0.69–1.26) 0.657	0.603	0.58 (0.38–0.89) **0.011**	0.143	0.84 (0.68–1.04) 0.106	0.180
			*H. pylori –*	90	104	43	68	89	21	0.99 (0.66–1.48) 0.866	0.908	0.62 (0.35–1.09) 0.089	0.686	0.87 (0.66–1.16) 0.451	0.836

Notes: Stratified analysis by *H. pylori* infection status

HC, healthy control; GC, gastric cancer; n, number of individuals.

A/a, major/minor alleles; OR, odds ratio; CI, confidence interval; NA, not applied.

^a^*P*-values, ORs, and 95% CIs adjusted by gender, age, smoking habit, and family history of GC.

^b^Q_*FDR*_-values obtained after applying the False Discovery Rate (FDR) test.

*P*-values < 0.05 are highlighted in bold.

None of the 108 DNA repair gene polymorphisms analyzed in our study were significantly associated with prevalence of infection with *H. pylori* or CagA/VacA strains (Supplementary Table 4). Of note, when subgroup analysis by *H. pylori* infection status was performed, the previously reported risk associations of *TP53* rs1042522, *TP53* rs9894946G, *LIG3* rs2074522, and *BRIP1* rs4986764 with GC were observed only in the group of *H. pylori*–infected individuals (Table 2). However, after FDR corrections, these associations did not remain significant with the exception of *TP53* rs1042522 (P72R). Tests for interaction under a multiplicative model showed no statistically significant interactions between *TP53* rs1042522 genotypes and *H. pylori* infection (*P*-_interaction_= 0.081, dominant model).

On the other hand, stratified analysis by tumor location (cardia/distal) and histological type of GC (intestinal/diffuse) showed significant associations with specific GC subtypes (Supplementary Tables 5 and 6). Table 3 summarizes those SNPs significantly associated with GC subtypes after applying the FDR correction test. Of interest, three SNPs (rs1799801, rs2238463, and rs3136038) located in the NER gene *ERCC4* were inversely associated with cardia GC risk. The *ERCC4* rs2238463 and rs3136038 loci were in strong linkage disequilibrium (LD) in our data set (D’ = 1, r^2^ = 0.92) with lower values for the rs1799801/rs2238463 (D’ = 0.98, r^2^ = 0.70), and rs1799801/rs3136038 (D’ = 0.93, r^2^ = 0.69) loci. In addition, carriers of the *XRCC3* rs861528A and *POLG* rs176641C variants were significantly increased in the subgroup of patients with non–cardia GC compared to controls (dominant models, OR: 1.45; 95% CI: 1.11–1.88; and OR: 1.40, 95% CI: 1.09–1.81, respectively). By contrast, the *XPC* rs2228000T variant was associated with a lower risk of non–cardia GC (dominant model, OR: 0.70; 95% CI: 0.55–0.90). When considering the histological type of tumor, the most remarkable association was observed in the DSBR gene *XRCC3*, with the A allele of rs861528 being associated with a higher risk of diffuse GC (dominant model, OR: 2.11; 95% CI: 1.43–3.12) (Table 3.).

**Table 3:** Association of DNA repair gene polymorphisms with anatomical and histological subtypes of gastric cancer

CARDIA GC			HC Genotype			GC Genotype			Dominant model		Recessive model		Log-Additive model	
Gen db SNP ID		A/a	AA	Aa	aa	AA	Aa	aa	OR (95% CI)^a^	FDR^b^	OR (95% CI)^a^	FDR^b^	OR (95% CI)^a^	FDR^b^
*ERCC4*	rs1799801	T/C	280	261	59	71	35	11	0.58 (0.38-0.88)	0.036	0.84 (0.40-1.77)	0.640	0.69 (0.49-0.97)	0.077
*ERCC4*	rs2238463	C/G	210	303	89	58	44	15	0.54 (0.35-0.81)	0.036	0.79 (0.42-1.49)	0.555	0.67 (0.49-0.92)	0.077
*ERCC4*	rs3136038	C/T	222	298	81	58	44	14	0.57 (0.38-0.87)	0.036	0.80 (0.42-1.53)	0.555	0.69 (0.50-0.96)	0.077
NON-CARDIA GC			HC Genotype			GC Genotype			Dominant model		Recessive model		Log-Additive model	
Gen	db SNP ID	A/a	AA	Aa	aa	AA	Aa	aa	OR (95% CI)^a^	FDR^b^	OR (95% CI)^a^	FDR^b^	OR (95% CI)^a^	FDR^b^
*XPC*	rs2228000	C/T	267	277	59	255	187	44	0.70 (0.55-0.90)	0.034	0.98 (0.64-1.48)	0.907	0.81 (0.67-0.98)	0.067
*XRCC3*	rs861528	G/A	350	205	33	218	176	27	1.45 (1.11-1.88)	0.034	1.21 (0.71-2.06)	0.626	1.31 (1.06-1.62)	0.067
*POLG*	rs176641	A/C	262	266	75	170	253	62	1.40 (1.09-1.81)	0.041	1.04 (0.72-1.50)	0.895	1.21 (1.00-1.46)	0.071
*TP53*	rs1042522	G/C	314	247	40	302	149	35	0.67 (0.52-0.87)	0.028	1.03 (0.76-1.97)	0.582	0.80 (0.65-0.98)	0.067
*LIG3*	rs2074522	G/A	509	89	2	393	81	12	1.37 (0.99-1.90)	0.084	7.94 (1.7-35.91)	0.027	1.46 (1.09-1.96)	0.067
*BRIP1*	rs4986764	C/T	224	270	107	197	230	56	0.87 (0.67-1.11)	0.315	0.59 (0.41-0.85)	0.037	0.81 (0.68- 0.97)	0.067
INTESTINAL GC			HC Genotype			GC Genotype			Dominant model		Recessive model		Log-Additive model	
Gen	db SNP ID	A/a	AA	Aa	aa	AA	Aa	aa	OR (95% CI)^a^	FDR^b^	OR (95% CI)^a^	FDR^b^	OR (95% CI)^a^	FDR^b^
*ERCC5*	rs17655	C/G	318	234	49	157	73	18	0.60 (0.43-0.82)	0.018	0.75 (0.40-1.39)	0.491	0.69 (0.53-0.89)	0.046
*BRIP1*	rs4986764	C/T	224	270	107	93	130	25	0.95 (0.69-1.31	0.809	0.48 (0.29-0.78)	0.026	0.82 (0.65-1.02)	0.157
DIFFUSE GC			HC Genotype			GC Genotype			Dominant model		Recessive model		Log-Additive model	
Gen	db SNP ID	A/a	AA	Aa	aa	AA	Aa	aa	OR (95% CI)^a^	FDR^b^	OR (95% CI)^a^	FDR^b^	OR (95% CI)^a^	FDR^b^
*XRCC3*	rs861528	G/A	350	205	33	66	73	13	2.11 (1.43-3.12)	0.001	1.75 (0.87-3.51)	0.239	1.75 (1.30-2.37)	0.003
*XRCC3*	rs861531	G/T	230	277	88	50	96	31	1.89 (1.26-2.84)	0.009	1.17 (0.72-1.89)	0.685	1.39 (1.07-1.81)	0.077
*APEX1*	rs1130409	T/G	155	300	145	62	78	38	0.61 (0.41-0.89)	0.044	0.76 (0.49-1.19)	0.333	0.74 (0.58-0.96)	0.078
*TP53*	rs1042522	G/C	314	247	40	113	53	12	0.64 (0.44-0.92)	0.046	1.01 (0.52-1.98)	0.723	0.77 (0.57-1.05)	0.185

HC, healthy control; GC, gastric cancer; A/a, major/minor alleles; OR, odds ratio; CI, confidence interval.

^a^ORs and 95% CIs were calculated by logistic regression analysis adjusted by gender, age, *H. pylori* infection, smoking, and family history of GC.

^b^Q_*FDR*_-values obtained after applying the False Discovery Rate (FDR) test. Q_*FDR*_-values < 0.05 are highlighted in bold.

Finally, no significant differences in genotype distribution and allele frequencies between GC patients and controls were found when subjects were stratified according to other evaluated features such as age, gender, smoking habit, and family history of GC (Supplementary Tables 7, 8, 9, 10).

### Haplotype analysis

**Figure 1:**
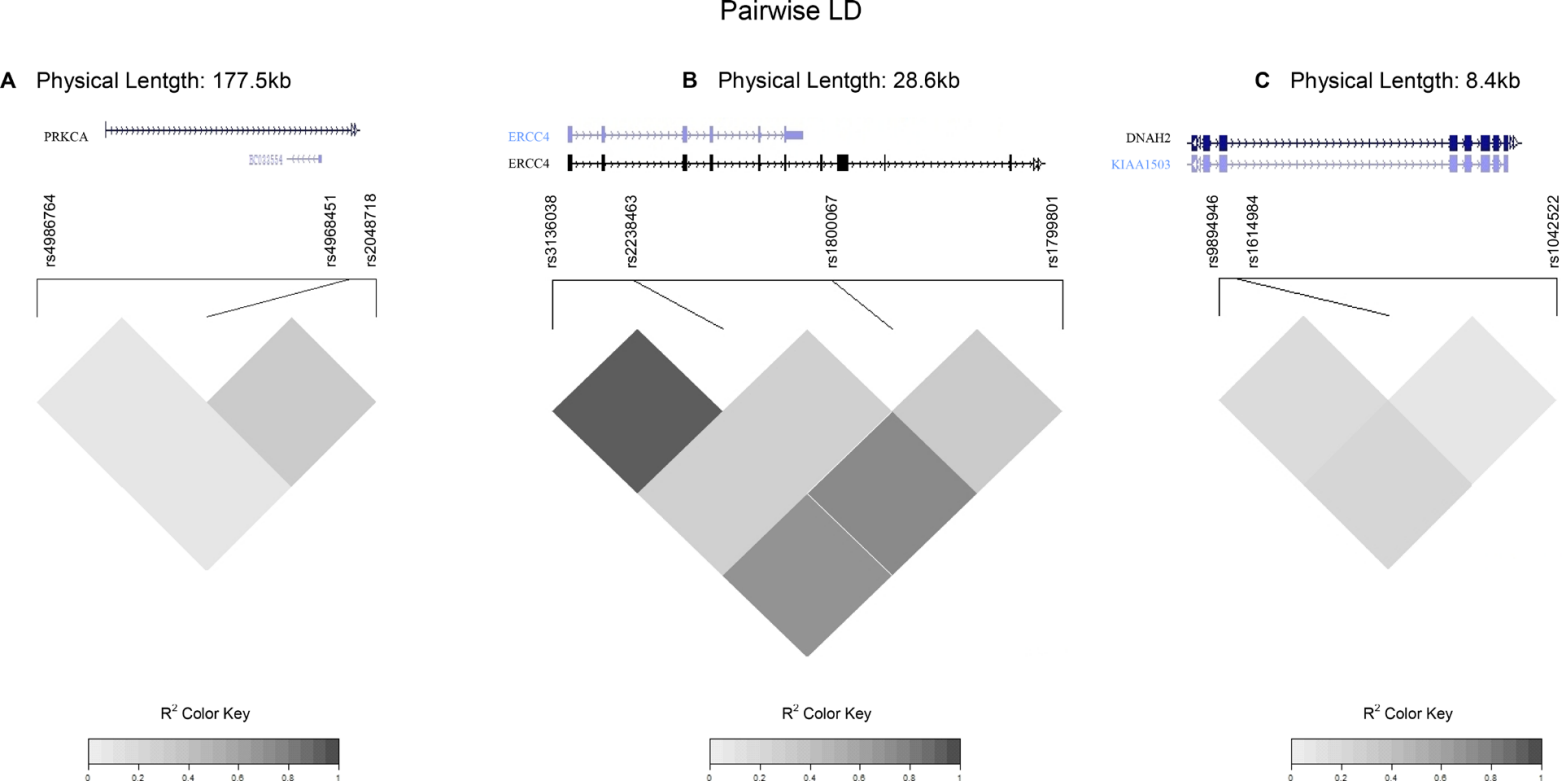
Linkage disequilibrium (LD) maps covering the *BRIP1*, *ERCC4*, and *TP53* genes LD maps based on pairwise r^2^ values for SNPs investigated in the (**A**) *BRIP1* gene, (**B**) *ERCC4* gene, and (**C**) *TP53* gene. Gene annotations obtained from the UCSC genome browser. Only LD maps of haplotypes in DNA repair genes significantly associated with gastric cancer risk (*P* < 0.05) are shown in the figure.

The comparisons of common haplotype frequencies (> 0.05) in each gene block between GC patients and controls are presented in Supplementary Table 11. Haplotype analysis revealed significant differences in four blocks covering the *BRIP1*, *ERCC4*, *ERCC5*, and *TP53* genes (Table 4, Figure 1). The most robust association was observed in the *TP53* block. Thus, the haplotype rs1042522C, rs1614984C, and rs9894946A, at a frequency of 10% in our population, was inversely associated with GC risk (12% in HC *vs*. 8% in GC; OR: 0.68; 95% CI: 0.51–0.91). This haplotype contains the rs1042522C and rs9894946A alleles previously reported in the single SNP analysis as protective factors for the development of GC. In the same way, the *BRIP1* (rs2048718T, rs4968451C, rs4986764T) and *ERCC4* (rs1799801T, rs1800067G, rs2238463G, rs3136038T) haplotypes carry the risk alleles identified in the single SNP–based analysis, associated with overall GC risk and cardial subtype, respectively. Stratified haplotype analysis by tumor location and GC histological type did not provide additional information beyond individual SNP results (data not shown).

**Table 4:** DNA repair gene haplotypes associated with gastric cancer risk

Gene	SNPs	Haplotype	Frequency HC	Frequency GC	OR (95% CI)^a^	*P*-value
***BRIP1***	rs2048718	CAC	0.3730	0.4043	Reference	
	rs4968451	CAT	0.2021	0.1852	0.85 (0.65–1.1)	0.2203
	rs4986764	TAC	0.1622	0.1609	0.91 (0.69–1.20)	0.5112
		TCT	0.1117	0.0888	0.73 (0.53–0.99)	**0.0459**
		TCC	0.0621	0.0712	1.07 (0.71–1.62)	0.7384
***ERCC4***	rs1799801	TGCC	0.5944	0.6408	Reference	
	rs1800067	CGGT	0.1857	0.1666	0.83 (0.66–1.03)	0.0927
	rs2238463	CAGT	0.1167	0.1134	0.89 (0.69–1.16)	0.3962
	rs3136038	TGGT	0.0790	0.0599	0.70 (0.50–0.96)	**0.0291**
***ERCC5***	rs1047768	CC	0.5330	0.5460	Reference	
	rs17655	TG	0.2335	0.1908	0.80 (0.66–0.98)	**0.0350**
		TC	0.1908	0.2155	1.11 (0.89–1.37)	0.3500
***TP53***	rs1042522	GCG	0.4331	0.4417	Reference	
	rs1614984	GTG	0.2490	0.2939	1.13 (0.92–1.38)	0.2367
	rs9894946	CTG	0.1347	0.1180	0.88 (0.67–1.14)	0.3292
		CCA	0.1222	0.0847	0.68 (0.51–0.91)	**0.0081**

HC, healthy control; GC, gastric cancer; OR, odds ratio; CI, confidence interval.

Haplotypes with frequencies > 0.05 are shown in the table.

^a^Odds ratios and 95% confidence intervals were calculated according to the additive genetic model taking as a reference the more common haplotype.

*P*-values < 0.05 are highlighted in bold.

### Multivariate analysis

In summary, of the environmental and genetic factors evaluated in this study, logistic regression analysis identified *H. pylori* infection with CagA strains (OR: 1.99; 95% CI: 1.55–2.54), smoking habit (OR: 1.77; 95% CI: 1.22–2.57), and positive family history of GC (OR: 2.87; 95% CI: 1.85–4.45) as independent risk factors for the development of GC. Concerning genetic factors, the *LIG3* rs2074522 polymorphism was associated with a higher risk of GC (OR: 5.67; 95% CI: 1.24–25.95, recessive model) whereas the *TP53* rs9894946 (OR:0.73; 95% CI: 0.56–0.96, log-additive model), *TP53* rs1042522 (OR: 0.76; 95% CI: 0.56–0.96, dominant model), and *BRIP1* rs4986764 (OR: 0.55; 95% CI: 0.38–0.78, recessive model) variants were associated with a lower risk of developing the disease (Table 5).

**Table 5:** Logistic regression analysis environmental and genetic factors associated with GC risk

Factor	OR	(95% CI)	*P*-value
Gender	0.98	(0.72–1.33)	0.892
Age	1.00	(0.99–1.01)	0.533
Infection with *H. pylori* CagA+	1.99	(1.55–2.54)	**0.000**
Tobacco (being current smoker)	1.77	(1.22–2.57)	**0.003**
Positive family history of GC	2.87	(1.85–4.45)	**0.000**
*TP53* rs1042522 allele C (dominant model)	0.76	(0.58–0.98)	**0.043**
*TP53* rs9894946 allele A (log-additive model)	0.73	(0.56–0.96)	**0.027**
*LIG3* rs2074522 allele A (recessive model)	5.67	(1.24–25.95)	**0.025**
*BRIP1* rs4986764 allele T (recessive model)	0.55	(0.38–0.78)	**0.001**

GC, gastric cancer; OR, odds ratio; 95% CI, 95% confidence interval.

*P*- values < 0.05 are highlighted in bold.

## DISCUSSION

Over the last few years, numerous studies concerning the association between DNA repair gene polymorphisms and GC risk have been conducted in different geographic areas and ethnic groups. However, most studies have yielded inconsistent and discrepant results [18, 19, 22, 23]. To assess the relevance of DNA repair gene polymorphisms to GC susceptibility and phenotype, we analyzed a total of 123 SNPs located in 52 genes involved in different DNA repair pathways.

In our population, four SNPs located in the *TP53* (rs1042522G>C, rs9894946G>A), *LIG3* (rs2074522G>A), and *BRIP1* (rs4986764C>T) genes were significantly associated with GC susceptibility after FDR multiple test correction. Thus, allele A of *LIG3* rs2074522 was associated with a higher risk of GC whereas the *TP53* rs9894946A, *TP53* rs1042522C, and *BRIP1* rs4986764T variants were associated with a lower risk of developing the disease. Interestingly, these four variants are located in the long (*LIG3* q11.2–q12, *BRIP1* q22.2) and short arms (*TP53* p.13.1) of chromosome 17, suggesting that this region of the genome represents a promising target for more extensive investigations in the field of GC research.

*TP53* (tumor protein p53) is a tumor suppressor gene that encodes a potent nuclear transcription factor with a fundamental role in the maintenance of genomic stability. When activated in response to cellular stress, the p53 protein induces cell cycle arrest and apoptosis, inhibits cell growth, and interacts with proteins involved in DNA repair [24]. High frequencies of somatic mutations in the *TP53* gene and/or overexpression of p53 protein have been reported in many types of human cancers, including GC [25]. Although *TP53* is a highly polymorphic gene, the most explored polymorphism is a nonsynonymous SNP (rs1042522G>C) located in a proline–rich domain in exon 4, which causes a proline–to–arginine substitution at codon 72 (Arg72Pro) [26]. Evidence indicates that this change in amino acid sequence affects the biochemical and biological functions of p53 [27], suggesting that the rs1042522 SNP may be relevant in cancer development. In this context, a number of case–control studies have reported the association of both the rs1042522G (Arg) and rs1042522C (Pro) variants with GC risk [28, 29]. Allele frequencies of rs1042522G>C differ notably among populations with values for the rs1042522C (Pro) variant ranging from ~63% in African Blacks to ~41% in Asians or ~17% in Swedish Saamis [30]. The relevance of these major ethnic and geographical variations in *TP53* rs1042522 profiles are supported by two recent meta–analyses showing a significant association between the rs1042522C (Pro) variant and GC in Eastern Asian populations but not in Caucasians and South Americans [31, 32]. Similarly, some opposing associations have been reported among ethnicities when considering the location and histological subtypes of GC [33, 34]. However, and as the authors note, the specific meta–analysis performed in Caucasians comprised very few studies, which were limited by sample size, differed in methodology, or lacked information about other well-documented risk factors for GC, such as *H. pylori* infection, tobacco smoking, and diet. In line with our results, Pérez-Pérez *et al*. [28] and Zhang *et al*. [32] observed a significantly lower frequency of the rs1042522C (Pro) allele in GC patients compared to HCs. Moreover, an Italian study by De Feo *et al*. [35] showed a significant interaction between both *TP53* rs1042522C and rs1625895A minor alleles and protection against GC.

The rs1042522 SNP is located in a proline–rich domain of the *TP53* gene, which is essential for the regulation of p53–mediated apoptosis. In this regard, Marin *et al*. [36] first reported the contribution of rs1042522 allele variants to the induction of apoptosis in p53 mutant cells. According to the authors, p53 mutants encoded by the rs1042522G (Arg) allele are preferentially selected during tumorigenesis because they prevent tumor cells from apoptotic cell death. Moreover, Schneider-Stock *et al*. [37] demonstrated that the rs1042522C (Pro) allele induces FasL/Fas–mediated apoptosis of tumor cells by cytotoxic T lymphocytes more effectively than does the rs1042522G (Arg) allele. Taken together, these results indicate a positive association between rs1042522G (Arg) status and reduction of apoptotic tumor cell death, an inference that is in line with the association of the *TP53* rs1042522C (Pro) variant and lower risk of GC observed in our population.

A second polymorphism in the *TP53* gene (rs9894946G>A), located in intron 10, was identified as a protective factor for GC development in our population. In line with these findings, Sprague *et al*. [38] reported a ~40% reduction in invasive breast cancer risk among women < 50 years carrying the rs9894946A allele. However, two other studies by García-Closas *et al*. [39] and Schildkraut *et al*. [40] found no evidence of association for rs9894946 and cancer development. Although variations in intronic structure have been proposed to influence cancer susceptibility via regulation of gene expression, gene splicing, or mRNA stability [41], the functional relevance of rs9894946 for p53 expression or function is still unknown. It is also plausible that this intronic polymorphism is in LD with other functional SNPs that may affect cancer risk. In our study, the *TP53* rs1042522 and rs9894946 variants showed a moderate LD (D’ = 0.63, r^2^ = 0.22). Haplotype analysis did not provide additional information beyond individual SNP results, and haplotype *TP53* rs1042522C, rs1614984C, rs9894946A, containing both rs1042522C and rs9894946A protective alleles, was inversely associated with GC risk in our population. To our knowledge, this is the first study reporting the link between rs9894946 and risk of GC. Therefore, further studies with larger populations and different ethnic groups are required to conclusively assess the relevance of this SNP for GC development.

As noted, the nonsynonymous rs4986764C>T polymorphism (S919P) located in exon 18 of the *BRIP1* gene was associated with a lower risk of GC in our study. The *BRIP1* (BRCA1-interacting protein 1) gene encodes a DEAH-box DNA helicase that directly interacts with the C–terminal domain of *BRCA1*. This bound complex is crucial for the normal double–strand break repair function of BRCA1 and checkpoint functions [42]. The *BRIP1* rs2048718, rs4986764, and rs4968451 SNPs, all evaluated in our study, have been associated with susceptibility to meningioma [43], breast, and ovarian cancer [44]. However, no previous studies have addressed the contribution of *BRIP1* gene variants to GC risk. In line with our results, Ma *et al*. [45] reported a protective effect of the rs4986764T allele against cervical cancer in a Chinese Han population, and individuals carrying the rs11079454T–rs4986763T–rs4986764T haplotype were less prone to cervical cancer. The same authors demonstrated that BRIP1 mRNA levels correlated with rs4986764 genotypes [46]. Based on the major anti–oncogenic role of the *BRIP1* pathway, a low–level BRIP1 activation associated with the rs4986764C allele may lead to cancer development through an impaired DNA repair process. The scarcity of *BRIP1* association studies highlights the need to characterize the genetic variation defined by the rs4986764 SNP and the functional consequences affecting BRIP1 expression or protein function.

Similar to *BRIP* variants, knowledge is very limited about the influence of *LIG3* (DNA ligase 3) gene polymorphisms to GC cancer susceptibility. *LIG3* is one of three mammalian genes encoding DNA ligases I, III, and IV. These proteins catalyze the joining of DNA ends although they each have a distinct functional significance [47]. DNA Lig III participates in the BER pathway and DNA single strand break repair by forming a stable complex with XRCC1. Polymorphisms in the *LIG3* gene have been associated with increased risk of several cancers such as colon [48], lung [49], and esophageal cancer [50]. In the present study, the rare allele A of the intronic rs2074522 variant (MAF: 0.089) was significantly associated with a higher overall risk of GC. In contrast with our findings, patients homozygous for the rs2074522 A allele were less prone to developing pancreatic cancer in a US study by Li *et al*. [51]. In addition to tissue–specific factors, these discrepant results could be explained by the low frequency of the rs2074522 AA genotype among Caucasians. In our population, the frequencies of the rs2074522 AA genotype in healthy individuals (0.33%) and GC patients (2.15%) were similar to those reported in European populations. Therefore, although *Q*_FDR_ values for *LIG3* rs2074522 retained significance in several genetic models, our results should be interpreted with caution and confirmed in future studies with larger sample sizes.

Stratified SNP analysis by tumor location (cardia/non–cardia) and histological type of GC (intestinal/diffuse) revealed some additional significant associations. Of interest, three SNPs (rs1799801T>C, rs2238463C>G, and rs3136038C>T) located in the NER gene *ERCC4* (excision repair cross-complementary group 4) and in strong LD with each other were inversely associated with cardia GC risk. The *ERCC4* gene, also known as *XPF*, is a key component of the NER pathway that also plays an important role in removal of DNA interstrand cross–links and DNA double-strand breaks [52]. Information concerning the relevance of *ERCC4* gene variants to GC susceptibility is very limited. To date, only studies performed in Asians have been published. Two Chinese reports by Gong *et al*. [53] and He *et al*. [54] found no association between risk of GC and rs6498486T>G, a tag SNP located in the promoter region which tags the rs3136038 SNP evaluated in our study. Similarly, Zhang *et al*. [55] observed a non–significantly decreased risk in patients carrying the rs180067 G or rs1799801 T alleles. In agreement with these results, we found no significant associations between *ERCC4* variants and overall risk of GC after FDR multiple test corrections. However, carriers of the minor rs1799801C, rs2238463G, or rs3136038T alleles had a significantly lower risk of developing cardia GC. Functional studies by Shi *et al*. [56] reported higher XPF transcript expression levels in subjects carrying the rs1799801 CC genotype compared to those carrying the wild T allele, a finding that is biologically plausible with the protective effect of the rs1799801 C variant observed in our study.

When considering the histological type of tumor, the most remarkable associations were observed in the DSBR gene *XRCC3* (X-ray repair cross complementing 3), with the rs861528G>A and rs861531G>T intronic variants being associated with a higher risk of diffuse GC. The *XRCC3* gene encodes a member of the RecA/Rad51–related protein family that functions in homologous recombination repair of DNA double–strand breaks [57]. The most explored polymorphism is a C>T transition in exon 7 (rs861539), which causes a threonine–to–methionine substitution at codon 241 (Thr241Met) [58]. In agreement with a recent meta–analysis [59], we found no evidence of association for rs861539 and GC risk. However, FDR values (*Q*_FDR_ = 0.054) showed a borderline increased risk of diffuse GC in patients carrying the T allele. Unlike rs861539, very few studies have been published on rs861528/rs861531 variants and cancer susceptibility, most of them reporting inconclusive results. In the current work, rs861528 and rs861531 showed a moderate–high LD (D’ = 0.97, r^2^ = 0.55). Of interest, the rs861531 SNP was highly linked with the functional rs861539 Thr241Met variant (D’ = 0.99, r^2^ = 0.94), with lower values for rs861528 and rs861539 (D’ = 0.90, r^2^ = 0.47). Despite the potential influence of intronic variants in gene regulation, the functional relevance of the susceptible intronic rs861528 and rs861531 SNPs remains unknown. Taken together, these major differences in genotype distribution and environmental exposures observed in our study among GC subtypes reveal the marked heterogeneity of GC and highlight the need to investigate each type separately when possible. Because GC subtypes may result from different pathogenic mechanisms, this strategy of refining the phenotype may improve power for detecting genetic associations.

Finally, we also examined whether the contribution of DNA repair genes to GC risk could be modified by other risk factors identified in our study, such as *H. pylori* infection, smoking, and positive family history of GC. Whereas smoking and family history of GC showed no effect in modifying the contribution of DNA repair polymorphisms to GC, a significant association between the previously reported *TP53* rs1042522, rs9894946, *LIG3* rs2074522, and *BRIP1* rs4986764 variants and overall GC risk was observed among *H. pylori* infected patients. After FDR correction, only *TP53* rs1042522 (P72R) remained significant, although tests for interaction between rs1042522 and *H. pylori* infection did not reach statistical significance. Taking into account that none of the SNPs analyzed in our study were associated with prevalence of infection or CagA/VacA strains, our findings suggest that *TP53* rs1042522 is likely to be associated with GC development after bacterial infection occurs and not with susceptibility to *H. pylori* infection *per se*. In this context, mutants of p53 protein have been recently reported as potential markers of *H. pylori*–associated gastric carcinogenesis [60]. Moreover, inflammation induced by *H. pylori* infection results in the generation of DNA–damaging reactive oxygen and nitrogen species in gastric epithelial cells [61]. Under normal conditions, there is a balance between DNA damage and DNA repair; however, reduced DNA repair capacity associated with gene variants and increased DNA damage generated by *H. pylori* infection may alter this status and give rise to the accumulation of DNA damage and consequently cancer development. Unfortunately, the scarcity of studies [53, 62, 63] addressing this issue makes it very difficult to conclude whether *H. pylori* infection has any effect on the relationship between DNA repair gene variants and GC risk.

Our study has several strengths and limitations. A comprehensive analysis of 123 SNPs in candidate DNA repair genes, some of them not previously evaluated for the risk of GC, was carried out in a homogeneous population of Spanish Caucasian subjects (603 HCs and 603 GC patients). To our knowledge, the current study is the first to show a significant effect of *TP53* rs9894946, *LIG3* rs2074522, and *BRIP1* rs4986764 variants on GC susceptibility. Moreover, additional associations with specific anatomic locations and histological subtypes of GC were observed. The fact that these associations remained significant after FDR multiple test corrections indicates that our results may not be a chance finding. However, some limitations also should be considered. In particular, although our study is one of the largest performed in Western populations, the sample size limited the power to detect small ORs, mainly in low–frequency variant polymorphisms. Taking into account the prevalence of the SNPs evaluated in our population and setting an a value of 0.05, the study had a power of 85% to detect ORs > 1.45 or < 0.70 except for the less prevalent variants (MAF: 0.05–0.10), with a power of 80% to observe OR > 1.97 in the whole data set. As a result, it is possible that we could have missed minor statistical differences, especially when subgroup analyses and assessment of gene–environment interactions were performed.

In summary, we can conclude that the *TP53* (rs1042522, rs9894946G), *LIG3* (rs2074522G), and *BRIP1* (rs4986764) variants are involved in the susceptibility to GC, particularly in subjects infected by *H. pylori*. Like many other complex diseases, GC is the result of a multifactorial interplay involving environmental, lifestyle, and host genetic factors. Because the magnitude of each etiologic factor might differ among populations, larger studies in different geographic areas and ethnic groups are warranted to elucidate the contribution of DNA repair gene polymorphisms and their interactions with other risk factors in the susceptibility to GC and phenotype.

## MATERIALS AND METHODS

### Subjects

A total of 684 Spanish Caucasian patients with primary GC diagnosed in a network of 16 general hospitals in Spain, from May 2003 to December 2010, were invited to take part in the study. Patients with gastric neoplasms other than adenocarcinoma, secondary or recurrent GC, previous history of other malignancies, or refusal to participate were excluded. Finally, 646 GC patients were initially selected as cases for this study. Gastric tumors were classified according to their histological type [3] as intestinal, diffuse, or indeterminate, and by anatomical location as proximal and non–cardia or distal GC. Information regarding demographic characteristics and potential risk factors including smoking habits and family history of GC were collected by a questionnaire administered by trained personnel as previously described [64].

The control group consisted of 646 Spanish, Caucasian, cancer–free volunteers with no previous history or symptoms of gastrointestinal disease, matched by gender, age (±5 years), and area of residence. Most controls were blood donors and individuals recruited from the outpatient clinical services in the same hospitals as cases. Eligible controls were also interviewed with the same standard questionnaire designed for patients.

Following completion of the interview, 10 ml of peripheral blood was collected from each participant for DNA extraction and serological study of *H. pylori* infection. Once processed, whole blood and serum samples were aliquoted and stored at −80°C until analysis. All patients and controls gave written informed consent to the study protocol, which was approved by the Ethical Committee of the Hospitals.

### *Helicobacter pylori* diagnosis

The presence of *H. pylori* infection was determined in GC patients by urease test (CLO-test; Delta West Ltd., Canning Vale, Bentley, Australia) and histological examination of biopsies taken at the antrum and corpus of the stomach during the endoscopic procedure. In addition, GC patients and controls were analyzed to determine in serum the presence of *H. pylori* infection and antibodies to CagA and/or to VacA antigens by western blot analysis (Bioblot Helicobacter, Izasa, Barcelona, Spain). This test for *H. pylori* infection and CagA/VacA antibodies has been previously validated in our area [65]. GC patients were considered positive for bacterial infection if any of the three tests was positive. However, for statistical and data analysis, only information related to western blot analysis in serum samples from CG patients and controls was considered.

### SNP selection and genotyping

All DNA repair gene polymorphisms evaluated in our study were selected from the NCBI data base (http://www.ncbi.nlm.nih.gov/snp), Genome build 38.p2. The panel of SNPs was chosen based on three criteria: (1) a reported prevalence of at least 5% for the less frequent allele among Caucasians; (2) potential functional consequences or (3) published evidence of an association with different types of cancer. We assessed a total of 123 SNPs located in 52 genes related to different DNA repair pathways (Supplementary Table 12). The selected panel comprised 18 SNPs located in genes involved in BER genes (*PARP1*, *OGG1*, *POLB*, *POLG*, *POLI*, *FEN1*, *APEX1*, *NEIL1*, *LIG1*, *LIG3*, *XRCC1*, *PCNA*), 26 SNPs located in NER genes (*ERCC1*, *ERCC2*, *ERCC3*, *ERCC4*, *ERCC5*, *ERCC6*, *XPA*, *XPC*, *POLE*), 29 SNPs located in MMR genes (*MUTHY*, *EXO1*, *MSH2*, *MSH3*, *MSH6*, *MLH1*, *MLH3*, *PMS2*), 38 SNPs located in DSBR genes by homologous recombination (*XRCC2*, *XRCC3*, *BRCA1*, *BRCA2*, *BRIP1*, *RAD51*, *RAD51B*, *RAD52*, *RAD54L*, *MRE11A*) or non–homologous end joining (*XRCC4*, *XRCC5*, *XRCC6*, *RAD50*, *WRN*, *NES1*, *LIG4*), 8 SNPs located in cell cycle checkpoint genes (*ATM*, *ATR*, *TP53*), and 4 SNPs located in genes (*MGMT*, *ALKBH2*, *ALKBH3*) coding for proteins involved in direct repair of DNA damage produced by alkylating agents.

Genomic DNA from patients and controls was extracted from ethylenediamine-tetraacetic acid–preserved whole blood using the QIAamp DNA Blood Mini extraction kit (Qiagen, Izasa, Barcelona, Spain). Genotyping was performed at the Spanish National Genotyping Centre (CEGEN-Santiago de Compostela) using the Illumina Veracode Platform (Illumina, Eindhoven, The Netherlands). As a quality control, 5% of samples, including internal controls by CEGEN, were analyzed in duplicate with a concordance rate of 100% for all assays. Among the 123 SNPs evaluated, 15 SNPs were excluded from the analysis due to failure of genotyping (*MUTYH* rs3219484, *MSH2* rs2303426, *ATR* rs2227928, *MSH3* rs184967, *ERCC6* rs2228526, *POLE* rs5744751, *RAD51B* rs10483813, *XRCC1* rs25489, *ERCC1* rs11615, *XRCC6* rs132788), SNP call rate < 90% (*BRCA1* rs3737559, *MSH2* rs1863332, *ERCC6* rs2228524, *MGMT* rs12917), or monomorphism (*POLB* rs12678588). Samples in which more than 20% of the SNPs failed genotyping were excluded (cases = 29, HCs = 15). In this study, genotype completion on genomic DNA samples exceeded 95%. Finally, after excluding 42 unmatched samples, 108 SNPs in 1206 subjects (603 GC patients and 603 HCs) were successfully genotyped and available for analysis.

### Statistical analysis

Genotype frequencies for each DNA repair gene polymorphism among controls were tested for Hardy–Weinberg equilibrium by a Chi-square (χ^2^) test with one degree of freedom (Supplementary Table 12). Genotype and allele frequencies between CG patients and controls were compared using the χ^2^ test with Yates’ correction or Fisher's exact test. The magnitude of the association of each polymorphism was estimated by ORs and 95% CIs using the *SNPassoc* package implemented in R. Analyses were performed using codominant, dominant, recessive, and log-additive genetic models. In addition, unconditional logistic regression analysis was conducted to quantify the influence of both genetic and environmental factors for GC as dependent variable. A variable was entered in the model if the significance level of its coefficient was less than 0.05 and was removed if it was greater than 0.10. Categorical variables included in the model were codified as dummy variables. For all tests, a two–sided *P* value < 0.05 was considered statistically significant. To address the issue of adjustment for multiple testing, the FDR test using a Benjamini–Hochberg method was applied [66]. Finally, comparison of common haplotype frequencies (> 0.05) in each gene block between GC patients and controls was performed. Estimated haplotype frequencies and LD coefficients (D’ and *r*^2^) were calculated using the *haplo.stats* package implemented in R. For each marker, the more common haplotype was used as the reference category. The statistical analyses were performed using SPSS 23.0 for Windows (SPSS Ibérica, Madrid, Spain).

Taking into account the prevalence of the analyzed SNPs in our population, the size of the study was sufficient to detect ORs > 1.45 or < 0.70 with a power of 80% and a a value of 0.05. For the less prevalent polymorphisms (MAF: 0.05–0.10), the study had a power of 80% to detect an OR of > 1.97 in the whole data set. All power calculations were performed using the program Epidat 4.1.

## SUPPLEMENTARY MATERIALS FIGURES AND TABLES

























## Abbreviations

*ALKBH2*: alkB homolog 2, alpha-ketoglutarate dependent dioxygenase
*ALKBH3*: alkB homolog 3, alpha-ketoglutarate dependent dioxygenase
*APEX1*: apurinic/apyrimidinic endodeoxyribonuclease 1
*ATM*: ATM serine/threonine kinase
ATR: ATR serine/threonine kinase
*BRCA1*: BRCA1, DNA repair associated
*BRCA2*: BRCA2, DNA repair associated
*BRIP1*: BRCA1 interacting protein C-terminal helicase 1
*ERCC1*: ERCC excision repair 1, endonuclease non-catalytic subunit
*ERCC2*: ERCC excision repair 2, TFIIH core complex helicase subunit
*ERCC3*: ERCC excision repair 3, TFIIH core complex helicase subunit
*ERCC4*: ERCC excision repair 4, endonuclease catalytic subunit
*ERCC5*: ERCC excision repair 5, endonuclease
*ERCC6*: ERCC excision repair 6, chromatin remodeling factor
*EXO1*: exonuclease 1
*FEN1*: flap structure-specific endonuclease 1
*LIG1*: DNA ligase 1
*LIG3*: DNA ligase 3
*LIG4*: DNA ligase 4
*MGMT*: O-6-methylguanine-DNA methyltransferase
MLH1: mutL homolog 1
MLH3: mutL homolog 3
*MRE11A*: MRE11 homolog, double strand break repair nuclease
*MSH2*: mutS homolog 2
*MSH3*: mutS homolog 3
*MSH6*: mutS homolog 6
*MUTYH*: mutY DNA glycosylase
NBS1: nibrin
*NEIL1*: nei like DNA glycosylase
*OGG1*: 8-oxoguanine DNA glycosylase
*PARP1*: poly(ADP-ribose) polymerase 1
*PCNA*: proliferating cell nuclear antigen
*PMS2*: PMS1 homolog 2, mismatch repair system component
*POLB*: DNA polymerase beta
*POLE*: DNA polymerase epsilon, catalytic subunit
*POLG*: DNA polymerase gamma, catalytic subunit
POLI: DNA polymerase iota
RAD50: RAD50 double strand break repair protein
RAD51B: RAD51 paralog B
RAD52: RAD52 homolog, DNA repair protein
RAD54L: RAD54-like
TP53: tumor protein p53
WRN: Werner syndrome RecQ like helicase
XPA: XPA, DNA damage recognition and repair factor
XPC: XPC complex subunit, DNA damage recognition and repair factor
XRCC1: X-ray repair cross complementing 1
XRCC2: X-ray repair cross complementing 2
XRCC3: X-ray repair cross complementing 3
XRCC4: X-ray repair cross complementing 4
XRCC5: X-ray repair cross complementing 5
XRCC6: X-ray repair cross complementing 6
