# Supplementary material for: Relevance of DNA repair gene polymorphisms to gastric cancer risk and phenotype
Source: Oncotarget. 2017 Mar 16;8(22):35848–62. doi: 10.18632/oncotarget.16261 (PMC5482622; doi:10.18632/oncotarget.16261)
Supplement: Supplementary file 1 [file oncotarget-08-35848-s001.pdf]

Relevance of DNA repair gene polymorphisms to gastric cancer risk and phenotype

Supplementary Materials

Supplementary Table 1: Demographic and clinical characteristics of HCs and GC patients according to the location and histological type of tumor

	Healthy Controls *n* = 603	Cardia GC *n* = 117	ORs (95% CI)	*P* Value	Non–Cardia GC *n* = 486	ORs (95% CI)	*P* Value
Age yrs ± SD (range)	70.17 ± 12.32 (30–96)	69 ± 11.06 (30–91)	0.99 (0.98–1.01)	0.67	70.59 ± 12.55 (30–96)	1 (0.99–1.01)	0.69
Gender	413 M (68.5%)	102 M (87.2%)			311 M (64%)		
	190 F (31.5%)	15 F (12.8%)	3.15 (1.78–5.57)	**< 0.0001**	175 F (36%)	0.83 (0.64–1.07)	0.14
Cigarette smoking							
Never	311/585 (53.2%)	33/114 (28.9%)	Reference		258/468 (55.1%)	Reference	
Former	184/585 (31.4%)	53/114 (46.5%)	2.71 (1.69–4.35)	**< 0.0001**	101/468 (21.6%)	0.71 (0.53–0.95)	**0.02**
Current smoker	90/585 (15.4%)	28/114 (24.6%)	2.93 (1.68–5.11)	**0.0002**	109/468 (23.3%)	1.35 (0.97–1.88)	0.07
H. pylori +	364 (60.4%)	67/109 (61.5%)	1.01 (0.72–1.44)	0.93	310/447 (69.3%)	1.49 (1.15–1.94)	**0.002**
CagA+	253 (41.9%)	45/109 (41.3%)	0.97 (0.68–1.32)	0.96	280/447 (62.6%)	2.32 (180–2.98)	**< 0.0001**
VacA+	237 (39.3%)	38/109 (34.9%)	0.82 (0.54–1.27)	0.39	185/447 (41.4%)	1.09 (0.85–1.40)	0.49
Family history of GC	33/539 (6.1%)	12/104 (11.5 %)	1.42 (0.68–3.01)	0.42	76/430 (17.7%)	3.29 (2.14–5.06)	**< 0.0001**
	**Healthy Controls *n* = 603**	**Intestinal GC *n* = 250**	**ORs (95% CI)**	***P* Value**	**Diffuse GC *n* = 178**	**ORs (95% CI)**	***P* Value**
Age yrs ± SD (range)	70.17 ± 12.32 (30–96)	72.82 ± 10.73 (33–92)	1.02 (1.01–1.03)	**0.004**	67.34 ± 14.32 (30–96)	0.98 (0.98–0.99)	**0.005**
Gender	413 M (68.5%)	167 M (66.8%)			101 M (56.7%)		
	190 F (31.5%)	83 F (33.2%)	0.98 (0.72–1.36)	0.94	77 F (43.3%)	0.58 (0.41–0.82)	0.002
Cigarette smoking							
Never	311/585 (53.2%)	134/240 (55.8%)	Reference		102/171 (59.6%)	Reference	
Former	184/585 (31.4%)	65/240 (27.1%)	0.82 (0.58–1.16)	0.29	40/171 (23.4%)	0.66 (0.44–1.00)	0.06
Current smoker	90/585 (15.4%)	41/240 (17.1%)	1.06 (0.69–1.61)	0.83	29/171 (17%)	0.98 (0.61–1.58)	1
H. pylori +	364 (60.4%)	158/231 (68.4%)	1.47 (1.06–2.03)	**0.02**	123/159 (77.3%)	2.17 (1.44–3.26)	**< 0.0001**
CagA+	253 (41.9%)	143/231 (61.9%)	2.25 (1.65–3.07)	**< 0.0001**	109/159 (68.5%)	3.02 (2.08–4.37)	**< 0.0001**
VacA+	237 (39.3%)	100/231 (43.3%)	1.21 (0.88–1.64)	0.23	68/159 (42.8%)	1.11 (0.78–1.59)	0.56
Family history of GC	33/539 (6.1%)	42/222 (18.9%)	3.58 (2.20–5.82)	**< 0.0001**	23/159 (14.5%)	2.60 (1.47–4.56)	**0.001**

HCs, healthy controls; GC, gastric cancer; n, number of individuals; OR, odds ratio; CI, confidence interval; SD, standard deviation; M, male; F, female. OR and 95% CI values adjusted by sex and age. *P* values < 0.05 are highlighted in bold.

Supplementary Table 2: Genotype frequencies of DNA repair gene polymorphisms in gastric cancer patients and healthy controls. See Supplementary_Table_2

Supplementary Table 3: Significant SNPs in DNA repair genes associated with gastric cancer risk. See Supplementary_Table_3

Supplementary Table 4: Association of DNA repair gene polymorphisms with risk of *Helicobacter pylori* infection in healthy controls. See Supplementary_Table_4

Supplementary Table 5: Significant SNPs associated with gastric cancer risk. Association analysis stratified by anatomical location of the tumor. See Supplementary_Table_5

Supplementary Table 6: Significant SNPs associated with gastric cancer risk. Association analysis stratified by histological tumor type. See Supplementary_Table_6

Supplementary Table 7: Association of DNA repair gene polymorphisms with GC risk. Stratified analysis by age. See Supplementary_Table_7

Supplementary Table 8: Association of DNA repair polymorphisms with gastric cancer risk. Stratified analysis by gender. See Supplementary_Table_8

Supplementary Table 9: Association of DNA repair gene polymorphisms with gastric cancer risk. Stratified analysis by smoking status (never smokers / current smokers). See Supplementary_Table_9

Supplementary Table 10: Association of DNA repair gene polymorphisms with gastric cancer risk. Stratified analysis by family history of gastric cancer. See Supplementary_Table_10

Supplementary Table 11: Haplotypes in DNA repair genes and association with GC risk.
See Supplementary_Table_11

Supplementary Table 12: Characteristics of candidate SNPs analyzed in the study.
See Supplementary_Table_12
