# Supplementary material for: Relevance of DNA repair gene polymorphisms to gastric cancer risk and phenotype
Source: Oncotarget. 2017 Mar 16;8(22):35848–62. doi: 10.18632/oncotarget.16261 (PMC5482622; doi:10.18632/oncotarget.16261)
Supplement: Supplementary file 2 [file oncotarget-08-35848-s002.doc]

**Supplementary Table 2: Genotype frequencies of DNA repair gene polymorphisms in gastric cancer patients and healthy controls.**

|  | | | **Healthy Controls**  **n = 603** | | | | | **Gastric Cancer patients**  **n = 603** | | | | | **Anatomic location** | | | | | | | | **Histological type** | | | | | | | |
| --- | --- | --- | --- | --- | --- | --- | --- | --- | --- | --- | --- | --- | --- | --- | --- | --- | --- | --- | --- | --- | --- | --- | --- | --- | --- | --- | --- | --- |
|  |  |  |  |  |  |  |  |  |  |  |  |  | **Cardia GC** | | | | **Distal GC** | | | | **Intestinal GC** | | | | **Diffuse GC** | | | |
|  |  |  |  |  |  |  |  |  |  |  |  |  | **n = 117** | | | | **n = 486** | | | | **n = 250** | | | | **n = 178** | | | |
| **db SNP ID** | **Gene** | **A/a** | **AA** | **Aa** | **aa** | **N** | **MAF** | **AA** | **Aa** | **aa** | **N** | **MAF** | **AA** | **Aa** | **aa** | **MAF** | **AA** | **Aa** | **aa** | **MAF** | **AA** | **Aa** | **aa** | **MAF** | **AA** | **Aa** | **aa** | **MAF** |
| rs10079641 | *MSH3* | C/G | 472 | 117 | 4 | 593 | 0.105 | 473 | 110 | 6 | 589 | 0.104 | 94 | 22 | ― | 0.095 | 379 | 88 | 6 | 0.106 | 195 | 47 | 3 | 0.108 | 139 | 33 | 2 | 0.106 |
| rs1042522 | *TP53* | G/C | 314 | 247 | 40 | 601 | 0.272 | 372 | 192 | 39 | 603 | 0.224 | 70 | 43 | 4 | 0.218 | 302 | 149 | 35 | 0.225 | 150 | 80 | 20 | 0.240 | 113 | 53 | 12 | 0.216 |
| rs1047768 | *ERCC5* | C/T | 204 | 284 | 113 | 601 | 0.424 | 221 | 274 | 108 | 603 | 0.406 | 47 | 50 | 20 | 0.385 | 174 | 224 | 88 | 0.412 | 95 | 106 | 49 | 0.408 | 61 | 89 | 28 | 0.407 |
| rs1047840 | *EXO1* | G/A | 217 | 290 | 94 | 601 | 0.398 | 232 | 273 | 84 | 589 | 0.374 | 42 | 57 | 16 | 0.387 | 190 | 216 | 68 | 0.371 | 100 | 114 | 29 | 0.354 | 66 | 78 | 30 | 0.397 |
| rs1048771 | *RAD54L* | C/T | 465 | 125 | 5 | 595 | 0.113 | 413 | 108 | 10 | 531 | 0.121 | 88 | 18 | 1 | 0.093 | 325 | 90 | 9 | 0.127 | 160 | 53 | 5 | 0.144 | 121 | 29 | 4 | 0.120 |
| rs1051677 | *XRCC5* | T/C | 500 | 98 | 4 | 602 | 0.088 | 487 | 113 | 2 | 602 | 0.097 | 94 | 23 | ­― | 0.098 | 393 | 90 | 2 | 0.097 | 201 | 46 | 2 | 0.100 | 146 | 32 | ― | 0.090 |
| rs1051685 | *XRCC5* | A/G | 493 | 98 | 10 | 601 | 0.098 | 489 | 110 | 4 | 603 | 0.098 | 90 | 27 | ― | 0.115 | 399 | 83 | 4 | 0.094 | 201 | 47 | 2 | 0.102 | 149 | 27 | 2 | 0.087 |
| rs1052133 | *OGG1* | C/G | 367 | 210 | 26 | 603 | 0.217 | 381 | 195 | 27 | 603 | 0.206 | 74 | 37 | 6 | 0.209 | 307 | 158 | 21 | 0.206 | 153 | 83 | 14 | 0.222 | 118 | 53 | 7 | 0.188 |
| rs1059262 | *ALKBH2* | T/G | 398 | 181 | 22 | 601 | 0.187 | 417 | 162 | 23 | 602 | 0.173 | 81 | 30 | 6 | 0.179 | 336 | 132 | 17 | 0.171 | 174 | 66 | 10 | 0.172 | 129 | 43 | 5 | 0.150 |
| rs1060915 | *BRCA1* | T/C | 272 | 268 | 62 | 602 | 0.326 | 248 | 291 | 62 | 601 | 0.345 | 44 | 61 | 12 | 0.363 | 204 | 230 | 50 | 0.341 | 107 | 115 | 26 | 0.337 | 73 | 91 | 14 | 0.334 |
| rs11226 | *RAD52* | C/T | 211 | 281 | 96 | 588 | 0.402 | 165 | 253 | 112 | 530 | 0.450 | 28 | 56 | 23 | 0.477 | 137 | 197 | 89 | 0.443 | 74 | 99 | 45 | 0.433 | 42 | 79 | 33 | 0.471 |
| rs1130409 | *APEX1* | T/G | 155 | 300 | 145 | 600 | 0.492 | 180 | 282 | 139 | 601 | 0.466 | 33 | 53 | 30 | 0.487 | 150 | 229 | 106 | 0.455 | 75 | 121 | 53 | 0.456 | 62 | 78 | 38 | 0.433 |
| rs1136410 | *PARP1* | T/C | 459 | 138 | 6 | 603 | 0.124 | 438 | 154 | 11 | 603 | 0.146 | 85 | 30 | 2 | 0.145 | 353 | 124 | 9 | 0.146 | 187 | 56 | 7 | 0.140 | 122 | 54 | 2 | 0.163 |
| rs13180316 | *XRCC4* | G/A | 317 | 237 | 43 | 597 | 0.271 | 324 | 223 | 50 | 597 | 0.271 | 63 | 42 | 12 | 0.282 | 261 | 181 | 38 | 0.268 | 136 | 89 | 21 | 0.266 | 85 | 75 | 16 | 0.304 |
| rs13181 | *ERCC2* | T/G | 275 | 254 | 74 | 603 | 0.333 | 258 | 261 | 83 | 602 | 0.355 | 52 | 43 | 22 | 0.372 | 206 | 218 | 61 | 0.351 | 108 | 107 | 34 | 0.351 | 75 | 80 | 23 | 0.354 |
| rs1346044 | *WRN* | T/C | 360 | 207 | 34 | 601 | 0.229 | 374 | 202 | 22 | 598 | 0.206 | 79 | 33 | 3 | 0.170 | 295 | 169 | 19 | 0.214 | 158 | 85 | 6 | 0.195 | 104 | 64 | 9 | 0.232 |
| rs144848 | *BRCA2* | T/G | 305 | 256 | 39 | 600 | 0.278 | 295 | 256 | 50 | 601 | 0.296 | 59 | 49 | 9 | 0.286 | 236 | 207 | 41 | 0.299 | 111 | 116 | 23 | 0.324 | 94 | 68 | 14 | 0.273 |
| rs1478485 | *XRCC4* | C/T | 221 | 285 | 95 | 601 | 0.395 | 196 | 312 | 94 | 602 | 0.415 | 39 | 56 | 22 | 0.427 | 157 | 256 | 72 | 0.412 | 78 | 135 | 37 | 0.418 | 61 | 94 | 22 | 0.390 |
| rs1540354 | *MLH1* | T/A | 469 | 120 | 13 | 602 | 0.121 | 478 | 111 | 11 | 600 | 0.111 | 95 | 20 | 2 | 0.103 | 383 | 91 | 9 | 0.113 | 192 | 50 | 6 | 0.125 | 144 | 30 | 3 | 0.102 |
| rs1614984 | *TP53* | C/T | 227 | 273 | 88 | 588 | 0.382 | 183 | 252 | 90 | 525 | 0.411 | 31 | 57 | 18 | 0.439 | 152 | 195 | 72 | 0.405 | 72 | 97 | 44 | 0.434 | 62 | 69 | 23 | 0.373 |
| rs1618536 | *ERCC2* | G/A | 162 | 264 | 155 | 581 | 0.494 | 155 | 245 | 124 | 524 | 0.470 | 30 | 52 | 25 | 0.477 | 125 | 193 | 99 | 0.469 | 60 | 101 | 52 | 0.481 | 46 | 72 | 34 | 0.461 |
| rs1650697 | *MSH3* | C/T | 322 | 221 | 44 | 587 | 0.263 | 307 | 185 | 37 | 529 | 0.245 | 53 | 47 | 7 | 0.285 | 254 | 138 | 30 | 0.235 | 134 | 72 | 12 | 0.220 | 91 | 50 | 12 | 0.242 |
| rs174538 | *FEN1* | G/A | 289 | 242 | 57 | 588 | 0.303 | 259 | 214 | 51 | 524 | 0.302 | 45 | 53 | 9 | 0.332 | 214 | 161 | 42 | 0.294 | 116 | 83 | 17 | 0.271 | 73 | 61 | 16 | 0.310 |
| rs175080 | *MLH3* | G/A | 198 | 281 | 120 | 599 | 0.435 | 186 | 293 | 116 | 595 | 0.441 | 34 | 63 | 20 | 0.440 | 152 | 230 | 96 | 0.441 | 74 | 127 | 47 | 0.446 | 61 | 78 | 36 | 0.429 |
| rs1760944 | *APEX1* | C/A | 212 | 271 | 72 | 555 | 0.374 | 207 | 253 | 75 | 535 | 0.377 | 37 | 49 | 15 | 0.391 | 170 | 204 | 60 | 0.373 | 84 | 117 | 24 | 0.367 | 56 | 68 | 29 | 0.412 |
| rs17655 | *ERCC5* | C/G | 318 | 234 | 49 | 601 | 0.276 | 350 | 214 | 36 | 600 | 0.238 | 68 | 45 | 4 | 0.226 | 282 | 169 | 32 | 0.241 | 157 | 73 | 18 | 0.220 | 95 | 73 | 10 | 0.261 |
| rs176641 | *POLG* | A/C | 262 | 266 | 75 | 603 | 0.345 | 221 | 302 | 79 | 602 | 0.382 | 51 | 49 | 17 | 0.355 | 170 | 253 | 62 | 0.389 | 88 | 130 | 31 | 0.386 | 62 | 99 | 17 | 0.374 |
| rs1776148 | *EXO1* | G/A | 227 | 282 | 91 | 600 | 0.387 | 223 | 295 | 82 | 600 | 0.382 | 45 | 56 | 15 | 0.371 | 178 | 239 | 67 | 0.385 | 95 | 122 | 33 | 0.376 | 54 | 95 | 27 | 0.423 |
| rs1799793 | *ERCC2* | G/A | 292 | 249 | 62 | 603 | 0.309 | 277 | 252 | 73 | 602 | 0.331 | 58 | 40 | 19 | 0.333 | 219 | 212 | 54 | 0.330 | 113 | 110 | 26 | 0.325 | 81 | 76 | 21 | 0.331 |
| rs1799794 | *XRCC3* | A/G | 334 | 225 | 42 | 601 | 0.257 | 342 | 224 | 34 | 600 | 0.243 | 67 | 44 | 6 | 0.239 | 275 | 180 | 28 | 0.244 | 144 | 86 | 19 | 0.249 | 100 | 72 | 4 | 0.227 |
| rs1799796 | *XRCC3* | A/G | 338 | 230 | 31 | 599 | 0.244 | 359 | 217 | 27 | 603 | 0.225 | 72 | 39 | 6 | 0.218 | 287 | 178 | 21 | 0.226 | 142 | 99 | 9 | 0.234 | 112 | 56 | 10 | 0.213 |
| rs1799801 | *ERCC4* | T/C | 280 | 261 | 59 | 600 | 0.316 | 306 | 239 | 57 | 602 | 0.293 | 71 | 35 | 11 | 0.244 | 235 | 204 | 46 | 0.305 | 118 | 106 | 25 | 0.313 | 85 | 78 | 15 | 0.303 |
| rs1799955 | *BRCA2* | A/G | 358 | 218 | 27 | 603 | 0.226 | 354 | 230 | 18 | 602 | 0.221 | 66 | 47 | 4 | 0.235 | 288 | 183 | 14 | 0.218 | 152 | 91 | 6 | 0.207 | 102 | 71 | 5 | 0.228 |
| rs1799966 | *BRCA1* | A/G | 267 | 271 | 65 | 603 | 0.333 | 244 | 291 | 60 | 595 | 0.345 | 44 | 62 | 11 | 0.359 | 200 | 229 | 49 | 0.342 | 104 | 114 | 26 | 0.340 | 73 | 90 | 14 | 0.333 |
| rs1799977 | *MLH1* | A/G | 272 | 258 | 73 | 603 | 0.335 | 265 | 278 | 60 | 603 | 0.330 | 41 | 58 | 18 | 0.402 | 224 | 220 | 42 | 0.313 | 107 | 121 | 22 | 0.330 | 91 | 74 | 13 | 0.281 |
| rs1800067 | *ERCC4* | G/A | 458 | 129 | 7 | 594 | 0.120 | 465 | 124 | 6 | 595 | 0.114 | 97 | 19 | 1 | 0.090 | 368 | 105 | 5 | 0.120 | 187 | 54 | 3 | 0.123 | 135 | 40 | 1 | 0.119 |
| rs1800389 | *WRN* | T/C | 328 | 224 | 49 | 601 | 0.268 | 347 | 211 | 40 | 598 | 0.243 | 65 | 46 | 5 | 0.241 | 282 | 165 | 35 | 0.244 | 146 | 81 | 21 | 0.248 | 100 | 63 | 14 | 0.257 |
| rs1800734 | *MLH1* | G/A | 349 | 210 | 40 | 599 | 0.242 | 341 | 218 | 37 | 596 | 0.245 | 71 | 38 | 7 | 0.224 | 270 | 180 | 30 | 0.250 | 141 | 93 | 11 | 0.235 | 98 | 65 | 14 | 0.263 |
| rs1800935 | *MSH6* | T/C | 310 | 244 | 43 | 597 | 0.276 | 282 | 276 | 42 | 600 | 0.300 | 54 | 55 | 8 | 0.303 | 228 | 221 | 34 | 0.299 | 116 | 110 | 22 | 0.310 | 87 | 79 | 12 | 0.289 |
| rs1800975 | *XPA* | G/A | 282 | 256 | 65 | 603 | 0.320 | 296 | 248 | 59 | 603 | 0.303 | 47 | 50 | 20 | 0.385 | 249 | 198 | 39 | 0.284 | 128 | 102 | 20 | 0.284 | 96 | 69 | 13 | 0.267 |
| rs1801406 | *BRCA2* | A/G | 284 | 264 | 55 | 603 | 0.310 | 284 | 283 | 32 | 599 | 0.290 | 52 | 58 | 7 | 0.308 | 232 | 225 | 25 | 0.285 | 122 | 113 | 13 | 0.280 | 80 | 88 | 8 | 0.295 |
| rs1801516 | *ATM* | G/A | 447 | 146 | 9 | 602 | 0.136 | 433 | 157 | 12 | 602 | 0.150 | 84 | 31 | 2 | 0.150 | 349 | 126 | 10 | 0.151 | 174 | 68 | 7 | 0.165 | 126 | 49 | 3 | 0.154 |
| rs1802904 | *ATR* | A/G | 459 | 138 | 6 | 603 | 0.124 | 446 | 140 | 13 | 599 | 0.139 | 85 | 30 | 2 | 0.145 | 361 | 110 | 11 | 0.137 | 176 | 68 | 5 | 0.157 | 138 | 33 | 5 | 0.122 |
| rs1805386 | *LIG4* | T/C | 412 | 173 | 15 | 600 | 0.169 | 415 | 172 | 13 | 600 | 0.165 | 79 | 31 | 7 | 0.192 | 336 | 141 | 6 | 0.158 | 169 | 77 | 3 | 0.167 | 130 | 44 | 3 | 0.141 |
| rs1805388 | *LIG4* | C/T | 442 | 136 | 10 | 588 | 0.133 | 383 | 131 | 11 | 525 | 0.146 | 68 | 33 | 3 | 0.188 | 315 | 98 | 8 | 0.135 | 157 | 56 | 5 | 0.151 | 121 | 28 | 3 | 0.112 |
| rs1805794 | *NBS1* | C/G | 310 | 233 | 59 | 602 | 0.292 | 300 | 241 | 61 | 602 | 0.301 | 51 | 53 | 12 | 0.332 | 249 | 188 | 49 | 0.294 | 140 | 81 | 29 | 0.278 | 91 | 71 | 16 | 0.289 |
| rs1981928 | *MSH2* | T/A | 315 | 241 | 45 | 601 | 0.275 | 307 | 258 | 34 | 599 | 0.272 | 56 | 51 | 10 | 0.303 | 251 | 207 | 24 | 0.265 | 128 | 108 | 11 | 0.263 | 90 | 76 | 11 | 0.277 |
| rs2020911 | *MSH6* | A/T | 253 | 268 | 80 | 601 | 0.356 | 255 | 274 | 74 | 603 | 0.350 | 43 | 60 | 14 | 0.376 | 212 | 214 | 60 | 0.344 | 106 | 112 | 32 | 0.352 | 74 | 80 | 24 | 0.360 |
| rs2040639 | *XRCC2* | G/A | 175 | 291 | 133 | 599 | 0.465 | 194 | 284 | 117 | 595 | 0.435 | 35 | 56 | 24 | 0.452 | 159 | 228 | 93 | 0.431 | 79 | 120 | 47 | 0.435 | 58 | 81 | 37 | 0.440 |
| rs2048718 | *BRIP1* | C/T | 189 | 282 | 105 | 576 | 0.427 | 202 | 281 | 100 | 583 | 0.413 | 44 | 56 | 9 | 0.339 | 158 | 225 | 91 | 0.429 | 79 | 117 | 45 | 0.429 | 56 | 85 | 34 | 0.437 |
| rs20580 | *LIG1* | C/A | 171 | 286 | 143 | 600 | 0.477 | 155 | 295 | 150 | 600 | 0.496 | 29 | 59 | 28 | 0.496 | 127 | 236 | 121 | 0.494 | 68 | 124 | 56 | 0.476 | 51 | 84 | 43 | 0.478 |
| rs2074522 | *LIG3* | G/A | 509 | 89 | 2 | 600 | 0.078 | 496 | 94 | 13 | 603 | 0.100 | 103 | 13 | 1 | 0.064 | 393 | 81 | 12 | 0.108 | 207 | 38 | 5 | 0.095 | 145 | 30 | 3 | 0.101 |
| rs2075685 | *XRCC4* | G/T | 193 | 296 | 113 | 602 | 0.434 | 174 | 319 | 109 | 602 | 0.446 | 32 | 61 | 24 | 0.466 | 142 | 258 | 85 | 0.441 | 71 | 135 | 44 | 0.446 | 54 | 99 | 24 | 0.415 |
| rs207906 | *XRCC5* | G/A | 464 | 127 | 12 | 603 | 0.125 | 466 | 127 | 9 | 602 | 0.12 | 83 | 33 | 1 | 0.150 | 383 | 94 | 8 | 0.113 | 198 | 48 | 3 | 0.108 | 140 | 34 | 4 | 0.118 |
| rs2228000 | *XPC* | C/T | 267 | 277 | 59 | 603 | 0.328 | 314 | 235 | 54 | 603 | 0.284 | 59 | 48 | 10 | 0.291 | 255 | 187 | 44 | 0.283 | 134 | 94 | 22 | 0.276 | 91 | 72 | 15 | 0.287 |
| rs2228001 | *XPC* | A/C | 220 | 284 | 94 | 598 | 0.395 | 207 | 231 | 92 | 530 | 0.392 | 42 | 43 | 22 | 0.407 | 165 | 188 | 70 | 0.388 | 82 | 97 | 38 | 0.399 | 58 | 70 | 26 | 0.396 |
| rs2228006 | *PMS2* | G/A | 436 | 140 | 20 | 596 | 0.151 | 371 | 146 | 13 | 530 | 0.162 | 69 | 34 | 4 | 0.196 | 302 | 112 | 9 | 0.154 | 154 | 57 | 7 | 0.163 | 108 | 44 | 1 | 0.150 |
| rs2238463 | *ERCC4* | C/G | 210 | 303 | 89 | 602 | 0.400 | 250 | 275 | 76 | 601 | 0.355 | 58 | 44 | 15 | 0.316 | 192 | 231 | 61 | 0.365 | 93 | 122 | 34 | 0.382 | 73 | 84 | 20 | 0.350 |
| rs2252775 | *RAD50* | A/C | 370 | 213 | 18 | 601 | 0.207 | 367 | 214 | 20 | 601 | 0.211 | 71 | 42 | 4 | 0.214 | 296 | 172 | 16 | 0.211 | 147 | 92 | 9 | 0.222 | 114 | 59 | 5 | 0.194 |
| rs2272615 | *POLB* | A/G | 459 | 125 | 11 | 595 | 0.124 | 464 | 120 | 11 | 595 | 0.119 | 92 | 23 | 2 | 0.115 | 372 | 97 | 9 | 0.120 | 197 | 44 | 6 | 0.113 | 128 | 43 | 3 | 0.141 |
| rs2286940 | *MLH1* | C/T | 170 | 280 | 149 | 599 | 0.482 | 154 | 308 | 139 | 601 | 0.488 | 31 | 61 | 25 | 0.474 | 129 | 247 | 108 | 0.478 | 67 | 125 | 57 | 0.480 | 47 | 92 | 38 | 0.475 |
| rs2303428 | *MSH2* | T/C | 495 | 98 | 9 | 602 | 0.095 | 476 | 120 | 6 | 602 | 0.110 | 84 | 32 | 1 | 0.145 | 392 | 88 | 5 | 0.101 | 204 | 41 | 4 | 0.098 | 138 | 39 | 1 | 0.115 |
| rs2308321 | *MGMT* | A/G | 476 | 90 | 11 | 577 | 0.097 | 468 | 97 | 2 | 567 | 0.089 | 88 | 18 | ― | 0.085 | 380 | 79 | 2 | 0.090 | 193 | 40 | ­― | 0.085 | 136 | 33 | 1 | 0.103 |
| rs2345060 | *PMS2* | A/G | 334 | 229 | 38 | 601 | 0.254 | 365 | 206 | 31 | 602 | 0.223 | 74 | 37 | 6 | 0.209 | 291 | 169 | 25 | 0.226 | 151 | 84 | 15 | 0.228 | 103 | 66 | 8 | 0.232 |
| rs2348244 | *MSH6* | T/C | 448 | 144 | 11 | 603 | 0.138 | 471 | 127 | 4 | 602 | 0.112 | 84 | 32 | 1 | 0.145 | 387 | 95 | 3 | 0.104 | 202 | 46 | 2 | 0.100 | 136 | 40 | 1 | 0.119 |
| rs238406 | *ERCC2* | G/T | 162 | 282 | 159 | 603 | 0.498 | 169 | 268 | 166 | 603 | 0.498 | 31 | 55 | 31 | 0.500 | 138 | 213 | 135 | 0.497 | 69 | 116 | 65 | 0.492 | 53 | 76 | 49 | 0.489 |
| rs2434470 | *ALKBH3* | C/G | 349 | 217 | 35 | 601 | 0.239 | 370 | 200 | 27 | 597 | 0.213 | 67 | 41 | 8 | 0.246 | 303 | 159 | 19 | 0.205 | 152 | 88 | 9 | 0.213 | 107 | 57 | 10 | 0.221 |
| rs2440 | *XRCC5* | C/T | 185 | 301 | 116 | 602 | 0.443 | 177 | 307 | 117 | 601 | 0.450 | 38 | 56 | 23 | 0.436 | 139 | 251 | 94 | 0.454 | 70 | 136 | 42 | 0.444 | 51 | 87 | 40 | 0.469 |
| rs25487 | *XRCC1* | G/A | 230 | 271 | 102 | 603 | 0.394 | 254 | 260 | 89 | 603 | 0.363 | 53 | 47 | 17 | 0.346 | 201 | 213 | 72 | 0.367 | 106 | 104 | 40 | 0.368 | 72 | 78 | 28 | 0.376 |
| rs26279 | *MSH3* | A/G | 287 | 243 | 70 | 600 | 0.319 | 281 | 254 | 68 | 603 | 0.323 | 61 | 47 | 9 | 0.278 | 220 | 207 | 59 | 0.334 | 112 | 104 | 34 | 0.344 | 84 | 77 | 17 | 0.312 |
| rs26779 | *MSH3* | G/A | 203 | 301 | 95 | 599 | 0.410 | 239 | 276 | 87 | 602 | 0.374 | 40 | 63 | 14 | 0.389 | 199 | 213 | 73 | 0.370 | 103 | 112 | 34 | 0.361 | 74 | 71 | 33 | 0.385 |
| rs293794 | *OGG1* | T/C | 410 | 168 | 19 | 597 | 0.173 | 401 | 190 | 10 | 601 | 0.175 | 76 | 40 | 1 | 0.179 | 325 | 150 | 9 | 0.174 | 178 | 68 | 4 | 0.152 | 110 | 62 | 4 | 0.199 |
| rs3136038 | *ERCC4* | C/T | 222 | 298 | 81 | 601 | 0.383 | 258 | 277 | 66 | 601 | 0.340 | 58 | 44 | 14 | 0.310 | 200 | 233 | 52 | 0.347 | 98 | 123 | 28 | 0.359 | 75 | 84 | 19 | 0.343 |
| rs3136228 | *MSH6* | T/G | 256 | 274 | 68 | 598 | 0.343 | 242 | 280 | 77 | 599 | 0.362 | 50 | 51 | 16 | 0.355 | 192 | 229 | 61 | 0.364 | 98 | 114 | 36 | 0.375 | 73 | 84 | 20 | 0.350 |
| rs3212948 | *ERCC1* | C/G | 250 | 262 | 89 | 601 | 0.366 | 243 | 268 | 85 | 596 | 0.367 | 50 | 55 | 12 | 0.338 | 193 | 213 | 73 | 0.375 | 99 | 112 | 36 | 0.372 | 72 | 73 | 30 | 0.380 |
| rs3212961 | *ERCC1* | C/A | 468 | 128 | 6 | 602 | 0.116 | 466 | 125 | 12 | 603 | 0.124 | 88 | 26 | 3 | 0.137 | 378 | 99 | 9 | 0.120 | 190 | 54 | 6 | 0.132 | 138 | 37 | 3 | 0.121 |
| rs3212986 | *ERCC1* | G/T | 329 | 223 | 43 | 595 | 0.260 | 294 | 200 | 37 | 531 | 0.258 | 65 | 37 | 5 | 0.220 | 229 | 163 | 32 | 0.268 | 122 | 84 | 12 | 0.248 | 80 | 59 | 15 | 0.289 |
| rs3213245 | *XRCC1* | T/C | 230 | 281 | 90 | 601 | 0.384 | 227 | 265 | 110 | 602 | 0.403 | 48 | 43 | 26 | 0.406 | 179 | 222 | 84 | 0.402 | 93 | 110 | 46 | 0.406 | 70 | 78 | 30 | 0.388 |
| rs3218536 | *XRCC2* | G/A | 486 | 113 | 3 | 602 | 0.099 | 483 | 115 | 5 | 603 | 0.104 | 100 | 15 | 2 | 0.080 | 383 | 100 | 3 | 0.109 | 193 | 56 | 1 | 0.116 | 142 | 34 | 2 | 0.107 |
| rs3219489 | *MUTYH* | G/C | 309 | 248 | 43 | 600 | 0.278 | 318 | 242 | 32 | 592 | 0.258 | 67 | 40 | 8 | 0.243 | 251 | 202 | 24 | 0.262 | 124 | 104 | 15 | 0.276 | 92 | 76 | 8 | 0.261 |
| rs3626 | *PCNA* | G/C | 455 | 135 | 12 | 602 | 0.132 | 460 | 136 | 7 | 603 | 0.124 | 88 | 29 | ― | 0.124 | 372 | 107 | 7 | 0.124 | 193 | 55 | 2 | 0.118 | 135 | 40 | 3 | 0.129 |
| rs3730668 | *POLI* | G/T | 186 | 314 | 98 | 598 | 0.426 | 199 | 288 | 107 | 594 | 0.423 | 36 | 49 | 29 | 0.469 | 163 | 239 | 78 | 0.411 | 84 | 115 | 46 | 0.422 | 60 | 92 | 25 | 0.401 |
| rs3793784 | *ERCC6* | C/G | 200 | 282 | 117 | 599 | 0.431 | 211 | 288 | 101 | 600 | 0.408 | 45 | 48 | 24 | 0.410 | 166 | 240 | 77 | 0.408 | 95 | 105 | 48 | 0.405 | 57 | 96 | 24 | 0.407 |
| rs4150416 | *ERCC3* | T/G | 269 | 273 | 59 | 601 | 0.325 | 301 | 255 | 47 | 603 | 0.289 | 51 | 61 | 5 | 0.303 | 250 | 194 | 42 | 0.286 | 132 | 94 | 24 | 0.284 | 86 | 79 | 13 | 0.295 |
| rs4150441 | *ERCC3* | A/G | 216 | 292 | 92 | 600 | 0.397 | 201 | 300 | 98 | 599 | 0.414 | 42 | 58 | 17 | 0.393 | 159 | 242 | 81 | 0.419 | 86 | 116 | 45 | 0.417 | 58 | 93 | 27 | 0.413 |
| rs4150474 | *ERCC3* | T/G | 325 | 242 | 33 | 600 | 0.257 | 353 | 220 | 30 | 603 | 0.232 | 65 | 49 | 3 | 0.235 | 288 | 171 | 27 | 0.231 | 149 | 87 | 14 | 0.230 | 104 | 65 | 9 | 0.233 |
| rs4234259 | *MLH1* | A/G | 165 | 278 | 154 | 597 | 0.491 | 150 | 309 | 141 | 600 | 0.493 | 31 | 63 | 23 | 0.466 | 127 | 246 | 110 | 0.482 | 65 | 124 | 59 | 0.488 | 47 | 92 | 38 | 0.475 |
| rs4253160 | *ERCC6* | A/T | 181 | 290 | 131 | 602 | 0.458 | 192 | 293 | 114 | 599 | 0.435 | 37 | 54 | 25 | 0.448 | 155 | 239 | 89 | 0.432 | 87 | 107 | 54 | 0.433 | 54 | 94 | 29 | 0.429 |
| rs4968451 | *BRIP1* | A/C | 407 | 176 | 17 | 600 | 0.175 | 422 | 166 | 13 | 601 | 0.160 | 82 | 33 | 2 | 0.158 | 340 | 133 | 11 | 0.160 | 172 | 71 | 5 | 0.163 | 121 | 53 | 4 | 0.171 |
| rs4986764 | *BRIP1* | C/T | 224 | 270 | 107 | 601 | 0.403 | 232 | 297 | 69 | 598 | 0.364 | 35 | 67 | 13 | 0.404 | 197 | 230 | 56 | 0.354 | 93 | 130 | 25 | 0.363 | 76 | 74 | 27 | 0.362 |
| rs4987876 | *ATM* | G/T | 497 | 94 | 9 | 600 | 0.093 | 511 | 79 | 11 | 601 | 0.084 | 96 | 20 | 1 | 0.094 | 415 | 59 | 10 | 0.082 | 217 | 26 | 7 | 0.080 | 151 | 23 | 2 | 0.077 |
| rs569143 | *MRE11A* | C/G | 158 | 297 | 146 | 601 | 0.490 | 158 | 308 | 132 | 598 | 0.478 | 29 | 59 | 28 | 0.496 | 130 | 249 | 103 | 0.472 | 66 | 127 | 54 | 0.476 | 51 | 89 | 37 | 0.460 |
| rs5744934 | *POLE* | A/G | 415 | 168 | 16 | 599 | 0.167 | 417 | 159 | 25 | 601 | 0.174 | 84 | 29 | 3 | 0.151 | 333 | 130 | 22 | 0.179 | 174 | 63 | 13 | 0.178 | 115 | 54 | 8 | 0.198 |
| rs601341 | *MRE11A* | G/A | 179 | 307 | 113 | 599 | 0.445 | 203 | 299 | 97 | 599 | 0.412 | 35 | 63 | 19 | 0.432 | 168 | 236 | 78 | 0.407 | 84 | 122 | 40 | 0.411 | 61 | 88 | 29 | 0.410 |
| rs6413436 | *RAD52* | T/C | 274 | 263 | 64 | 601 | 0.325 | 257 | 261 | 84 | 602 | 0.356 | 49 | 50 | 18 | 0.368 | 208 | 211 | 66 | 0.354 | 108 | 106 | 35 | 0.353 | 68 | 85 | 25 | 0.379 |
| rs664143 | *ATM* | C/T | 226 | 288 | 89 | 603 | 0.386 | 248 | 280 | 73 | 601 | 0.354 | 52 | 50 | 15 | 0.342 | 196 | 230 | 58 | 0.357 | 105 | 116 | 28 | 0.345 | 69 | 83 | 25 | 0.376 |
| rs7182283 | *NEIL1* | G/T | 171 | 280 | 144 | 595 | 0.477 | 169 | 309 | 118 | 596 | 0.457 | 29 | 65 | 22 | 0.470 | 140 | 244 | 96 | 0.454 | 70 | 126 | 49 | 0.457 | 55 | 86 | 36 | 0.446 |
| rs735943 | *EXO1* | C/T | 174 | 279 | 149 | 602 | 0.479 | 173 | 302 | 124 | 599 | 0.459 | 35 | 58 | 24 | 0.453 | 138 | 244 | 100 | 0.461 | 75 | 130 | 44 | 0.438 | 44 | 87 | 44 | 0.500 |
| rs7797466 | *PMS2* | G/A | 439 | 142 | 20 | 601 | 0.151 | 419 | 170 | 13 | 602 | 0.163 | 87 | 26 | 4 | 0.145 | 332 | 144 | 9 | 0.167 | 175 | 69 | 5 | 0.159 | 123 | 52 | 3 | 0.163 |
| rs799917 | *BRCA1* | C/T | 250 | 281 | 69 | 600 | 0.349 | 234 | 297 | 69 | 600 | 0.363 | 41 | 63 | 13 | 0.380 | 193 | 234 | 56 | 0.358 | 100 | 118 | 31 | 0.361 | 73 | 88 | 15 | 0.335 |
| rs8305 | *POLI* | A/G | 329 | 236 | 38 | 603 | 0.259 | 308 | 245 | 50 | 603 | 0.286 | 66 | 43 | 8 | 0.252 | 242 | 202 | 42 | 0.294 | 130 | 99 | 21 | 0.282 | 85 | 77 | 16 | 0.306 |
| rs861528 | *XRCC3* | G/A | 350 | 205 | 33 | 588 | 0.230 | 279 | 219 | 30 | 528 | 0.264 | 61 | 43 | 3 | 0.229 | 218 | 176 | 27 | 0.273 | 124 | 81 | 12 | 0.242 | 66 | 73 | 13 | 0.326 |
| rs861531 | *XRCC3* | G/T | 230 | 277 | 88 | 595 | 0.381 | 210 | 298 | 89 | 597 | 0.399 | 43 | 56 | 17 | 0.388 | 167 | 242 | 72 | 0.401 | 97 | 116 | 33 | 0.370 | 50 | 96 | 31 | 0.446 |
| rs861539 | *XRCC3* | C/T | 238 | 277 | 88 | 603 | 0.376 | 227 | 293 | 83 | 603 | 0.381 | 43 | 58 | 16 | 0.385 | 184 | 235 | 67 | 0.380 | 106 | 115 | 29 | 0.346 | 57 | 91 | 30 | 0.424 |
| rs9350 | *EXO1* | C/T | 430 | 150 | 19 | 599 | 0.157 | 439 | 143 | 14 | 596 | 0.143 | 87 | 25 | 4 | 0.142 | 352 | 118 | 10 | 0.144 | 184 | 58 | 4 | 0.134 | 127 | 45 | 4 | 0.151 |
| rs963248 | *XRCC4* | A/G | 422 | 165 | 15 | 602 | 0.162 | 419 | 168 | 16 | 603 | 0.166 | 83 | 33 | 1 | 0.150 | 336 | 135 | 15 | 0.170 | 164 | 77 | 9 | 0.190 | 127 | 48 | 3 | 0.152 |
| rs9876116 | *MLH1* | A/G | 173 | 279 | 149 | 601 | 0.480 | 161 | 308 | 131 | 600 | 0.475 | 29 | 64 | 24 | 0.479 | 137 | 244 | 102 | 0.464 | 70 | 120 | 58 | 0.476 | 52 | 93 | 32 | 0.444 |
| rs9894946 | *TP53* | G/A | 409 | 165 | 18 | 592 | 0.170 | 452 | 138 | 6 | 596 | 0.126 | 92 | 23 | ― | 0.100 | 360 | 115 | 6 | 0.132 | 188 | 56 | 4 | 0.129 | 123 | 51 | 2 | 0.156 |
|  |  |  |  |  |  |  |  |  |  |  |  |  |  |  |  |  |  |  |  |  |  |  |  |  |  |  |  |  |

n, number of individuals; A/a, major/minor alleles; MAF, minor allele frequency.

N, number of individuals successfully genotyped.
