# Supplementary material for: Relevance of DNA repair gene polymorphisms to gastric cancer risk and phenotype
Source: Oncotarget. 2017 Mar 16;8(22):35848–62. doi: 10.18632/oncotarget.16261 (PMC5482622; doi:10.18632/oncotarget.16261)
Supplement: Supplementary file 3 [file oncotarget-08-35848-s003.doc]

**Supplementary Table 3: Significant SNPs in DNA repair genes associated with gastric cancer risk.**

|  | | **Univariate analysis** | | | | **Multivariate analysis*** | | | |
| --- | --- | --- | --- | --- | --- | --- | --- | --- | --- |
|  |  | **Codominant** | **Dominant** | **Recessive** | **Log-additive** | **Codominant** | **Dominant** | **Recessive** | **Log-Additive** |
| **Gen** | **db SNP ID** | **OR (95% CI) *P* value** | **OR (95% CI) *P* value** | **OR (95% CI) *P* value** | **OR (95% CI) *P* value** | **OR (95% CI) *P* value** | **OR (95% CI) *P* value** | **OR (95% CI) *P* value** | **OR (95% CI)  *P* value** |
| *TP53* | rs1042522 | 0.82 (0.52–1.31) 0.471 | 0.68 (0.54–0.85) **0.001** | 0.97 (0.61-1.53) 0.912 | 0.78 (0.64–0.93) **0.007** | 0.88 (0.55–1.42) 0.609 | 0.67 (0.53–0.85) **0.001** | 1.05 (0.66–1.67) 0.832 | 0.78 (0.65–0.94) **0.009** |
| *RAD52* | rs11226 | 1.49 (1.06–2.10) **0.024** | 1.24 (0.96–1.59) 0.092 | 1.37 (1.02–1.86) **0.039** | 1.21 (1.03–1.43) **0.023** | 1.51 (1.06–2.13) **0.017** | 1.23 (0.95–1.59) 0.111 | 1.40 (1.03–1.91) **0.032** | 1.21 (1.02–1.44) **0.025** |
| *ERCC5* | rs17655 | 0.67 (0.42–1.05) 0.085 | 0.80 (0.64–1.01) 0.058 | 0.72 (0.46–1.12) 0.144 | 0.82 (0.69–0.99) **0.035** | 0.63 (0.39–1.01) 0.052 | 0.77 (0.61–0.97) **0.025** | 0.69 (0.43–1.09) 0.109 | 0.79 (0.66–0.96) **0.015** |
| *POLG* | rs176641 | 1.25 (0.86–1.79) 0.265 | 1.32 (1.05–1.67) **0.016** | 1.06 (0.76–1.49) 0.721 | 1.18 (1.00–1.39) 0.055 | 1.24 (0.86–1.80) 0.108 | 1.29 (1.02–1.63) **0.036** | 1.08 (0.76–1.53) 0.659 | 1.16 (0.98–1.38) 0.082 |
| *BRCA2* | rs1801406 | 0.58 (0.36–0.92) **0.028** | 0.99 (0.79–1.24) 0.913 | 0.56 (0.36–0.88) **0.011** | 0.90 (0.75–1.08) 0.252 | 0.61 (0.38–0.97) **0.038** | 0.98 (0.77–1.23) 0.837 | 0.60 (0.38–0.95) **0.027** | 0.90 (0.75–1.09) 0.277 |
| *LIG3* | rs2074522 | 6.67 (1.50–29.67) **0.009** | 1.21 (0.89-1.64) 0.227 | 6.59 (1.48–29.28) **0.002** | 1.30 (0.98 –1.71) 0.064 | 7.15 (1.60–31.97) **0.007** | 1.22 (0.89–1.67) 0.217 | 7.05 (1.58–31.53) **0.001** | 1.32 (0.99–1.75) 0.055 |
| *XPC* | rs2228000 | 0.78 (0.52–1.17) 0.257 | 0.73 (0.58–0.92) **0.006** | 0.91 (0.62–1.34) 0.621 | 0.82 (0.68–0.97) **0.021** | 0.82 (0.55–1.24) 0.829 | 0.73 (0.58–0.92) **0.008** | 0.96 (0.65–1.43) 0.854 | 0.83 (0.69–0.99) **0.035** |
| *ERCC4* | rs2238463 | 0.71 (0.50–1.02) 0.070 | 0.75 (0.60–0.95) **0.016** | 0.83 (0.60–1.16) 0.280 | 0.82 (0.70–0.97) **0.023** | 0.71 (0.49–1.03) 0.059 | 0.75 (0.59–0.95) **0.019** | 0.83 (0.59–1.17) 0.325 | 0.82 (0.69–0.98) **0.025** |
| *MGMT* | rs2308321 | 0.18 (0.04–0.84) **0.028** | 1.00 (0.73–1.35) 0.984 | 0.18 (0.04–0.83) **0.009** | 0.91 (0.69–1.20) 0.517 | 0.20 (0.04–0.93) **0.039** | 1.03 (0.75–1.41) 0.858 | 0.20 (0.04–0.91) **0.015** | 0.94 (0.71–1.25) 0.665 |
| *MSH3* | rs26779 | 0.77 (0.55–1.10) 0.159 | 0.78 (0.62–0.98) **0.036** | 0.90 (0.62–0.98) 0.496 | 0.86 (0.73–1.01) 0.068 | 0.80 (0.56–1.14) 0.242 | 0.81 (0.64–1.04) 0.093 | 0.90 (0.65–1.24) 0.509 | 0.88 (0.74–1.04) 0.127 |
| *ERCC4* | rs3136038 | 0.70 (0.48–1.01) 0.073 | 0.78 (0.62–0.98) **0.033** | 0.79 (0.56–1.12) 0.186 | 0.83 (0.70–0.98) **0.027** | 0.68 (0.46–0.99) **0.049** | 0.77 (0.61–0.98) **0.032** | 0.76 (0.53–1.09) 0.138 | 0.81 (0.68–0.97) **0.021** |
| *BRIP1* | rs4986764 | 0.62 (0.43–0.88) **0.009** | 0.94 (0.74–1.18) 0.586 | 0.60 (0.43–0.83) **0.002** | 0.85 (0.72–0.99) **0.049** | 0.62 (0.43–0.89) **0.007** | 0.94 (0.74–1.20) 0.633 | 0.59 (0.42–0.83) **0.002** | 0.85 (0.72–1.00) 0.055 |
| *XRCC3* | rs861528 | 1.14 (0.67–1.91) 0.690 | 1.31 (1.04–1.36) **0.024** | 1.01 (0.61–1.69) 0.959 | 1.20 (0.99–1.46) 0.061 | 1.20 (0.71–2.03) 0.432 | 1.35 (1.06–1.72) **0.015** | 1.05 (0.62–1.76) 0.862 | 1.23 (1.01–1.51) **0.038** |
| *TP53* | rs9894946 | 0.30 (0.11–0.76) **0.011** | 0.71 (0.55–0.92) **0.009** | 0.32 (0.13–0.82) **0.010** | 0.70 (0.56–0.88) **0.002** | 0.21 (0.07–0.64) **0.001** | 0.69 (0.53–0.90) **0.006** | 0.23 (0.08–0.68) **0.002** | 0.67 (0.53–0.85) **0.001** |

OR, odds ratio; 95% CI, 95% confidence interval.

ORs and 95% CI in the multivariate analysis were adjusted for age, gender, *H. pylori* infection, smoking habit, and family history of GC.

*P* values < 0.05 are highlighted in bold.
