# Supplementary material for: Relevance of DNA repair gene polymorphisms to gastric cancer risk and phenotype
Source: Oncotarget. 2017 Mar 16;8(22):35848–62. doi: 10.18632/oncotarget.16261 (PMC5482622; doi:10.18632/oncotarget.16261)
Supplement: Supplementary file 4 [file oncotarget-08-35848-s004.doc]

**Supplementary Table 4: Association of DNA repair gene polymorphisms with risk of *Helicobacter pylori* infection in healthy controls**.

|  | ***Helicobacter pylori* infection** | | | | | | **CagA status** | | | | | **VacA status** | | | | |
| --- | --- | --- | --- | --- | --- | --- | --- | --- | --- | --- | --- | --- | --- | --- | --- | --- |
|  |  | **Log-additive genetic model** | | | | | **Log-additive genetic model** | | | | | **Log-additive genetic model** | | | | |
|  |  |  | **95% CI** | |  |  |  | **95% CI** | |  |  |  | **95% CI** | |  |  |
| **db SNP ID** | **Gen** | **OR^a^** | **Lower** | **Upper** | ***P-*value** | **FDR^b^** | **OR^a^** | **Lower** | **Upper** | ***P-*value** | **FDR^b^** | **OR^a^** | **Lower** | **Upper** | ***P-*value** | **FDR^b^** |
| rs10079641 | *MSH3* | 0.72 | 0.49 | 1.05 | 0.092 | 0.583 | 0.88 | 0.52 | 1.50 | 0.651 | 0.964 | 0.65 | 0.39 | 1.07 | **0.025** | 0.672 |
| rs1042522 | *TP53* | 1.16 | 0.89 | 1.51 | 0.277 | 0.869 | 0.84 | 0.58 | 1.20 | 0.339 | 0.924 | 0.93 | 0.66 | 1.33 | 0.708 | 0.968 |
| rs1047768 | *ERCC5* | 1.13 | 0.90 | 1.42 | 0.301 | 0.869 | 0.95 | 0.70 | 1.29 | 0.741 | 0.964 | 0.81 | 0.60 | 1.09 | 0.170 | 0.708 |
| rs1047840 | *EXO1* | 0.95 | 0.75 | 1.20 | 0.654 | 0.989 | 0.70 | 0.51 | 0.97 | **0.033** | 0.924 | 0.99 | 0.73 | 1.36 | 0.963 | 0.973 |
| rs1048771 | *RAD54L* | 1.09 | 0.75 | 1.59 | 0.657 | 0.989 | 0.66 | 0.40 | 1.08 | 0.102 | 0.924 | 0.78 | 0.48 | 1.28 | 0.282 | 0.794 |
| rs1051677 | *XRCC5* | 1.15 | 0.76 | 1.74 | 0.517 | 0.989 | 0.71 | 0.41 | 1.21 | 0.208 | 0.924 | 0.66 | 0.39 | 1.11 | 0.120 | 0.672 |
| rs1051685 | *XRCC5* | 1.18 | 0.81 | 1.73 | 0.384 | 0.869 | 1.08 | 0.65 | 1.78 | 0.766 | 0.964 | 0.92 | 0.57 | 1.47 | 0.719 | 0.970 |
| rs1052133 | *OGG1* | 1.16 | 0.87 | 1.54 | 0.321 | 0.869 | 0.88 | 0.61 | 1.27 | 0.485 | 0.940 | 0.81 | 0.57 | 1.15 | 0.239 | 0.739 |
| rs1059262 | *ALKBH2* | 0.86 | 0.64 | 1.15 | 0.301 | 0.869 | 0.89 | 0.59 | 1.35 | 0.587 | 0.940 | 1.18 | 0.90 | 1.54 | 0.236 | 0.739 |
| rs1060915 | *BRCA1* | 1.05 | 0.82 | 1.35 | 0.706 | 0.989 | 1.17 | 0.83 | 1.64 | 0.371 | 0.924 | 1.02 | 0.74 | 1.41 | 0.903 | 0.973 |
| rs11226 | *RAD52* | 1.02 | 0.80 | 1.29 | 0.881 | 0.989 | 1 | 0.72 | 1.39 | 0.983 | 0.990 | 0.91 | 0.66 | 1.25 | 0.553 | 0.910 |
| rs1130409 | *APEX1* | 1 | 0.79 | 1.26 | 0.995 | 0.995 | 1.18 | 0.86 | 1.62 | 0.318 | 0.924 | 1.16 | 0.85 | 1.57 | 0.356 | 0.854 |
| rs1136410 | *PARP1* | 1.20 | 0.84 | 1.73 | 0.316 | 0.869 | 1.15 | 0.71 | 1.86 | 0.580 | 0.940 | 1.09 | 0.68 | 1.72 | 0.287 | 0.794 |
| rs13180316 | *XRCC4* | 0.94 | 0.72 | 1.21 | 0.615 | 0.989 | 1.34 | 0.92 | 1.96 | 0.118 | 0.924 | 0.8 | 0.56 | 1.13 | 0.198 | 0.708 |
| rs13181 | *ERCC2* | 0.93 | 0.74 | 1.18 | 0.570 | 0.989 | 0.99 | 0.71 | 1.38 | 0.943 | 0.964 | 0.75 | 0.54 | 1.03 | 0.074 | 0.672 |
| rs1346044 | *WRN* | 1.29 | 0.98 | 1.70 | 0.072 | 0.583 | 0.76 | 0.53 | 1.08 | 0.131 | 0.924 | 0.87 | 0.62 | 1.23 | 0.438 | 0.885 |
| rs144848 | *BRCA2* | 1.03 | 0.79 | 1.34 | 0.840 | 0.989 | 0.85 | 0.59 | 1.22 | 0.380 | 0.924 | 0.74 | 0.52 | 1.05 | 0.095 | 0.672 |
| rs1478485 | *XRCC4* | 1.12 | 0.89 | 1.42 | 0.330 | 0.869 | 0.98 | 0.71 | 1.35 | 0.895 | 0.964 | 1.01 | 0.73 | 1.38 | 0.973 | 0.973 |
| rs1540354 | *MLH1* | 0.97 | 0.69 | 1.36 | 0.856 | 0.989 | 1.54 | 0.91 | 2.60 | 0.097 | 0.924 | 0.82 | 0.52 | 1.30 | 0.402 | 0.885 |
| rs1614984 | *TP53* | 1.20 | 0.95 | 1.53 | 0.130 | 0.666 | 1.09 | 0.79 | 1.51 | 0.592 | 0.940 | 1.19 | 0.87 | 1.62 | 0.280 | 0.794 |
| rs1618536 | *ERCC2* | 1.21 | 0.96 | 1.51 | 0.101 | 0.583 | 0.90 | 0.67 | 1.23 | 0.522 | 0.940 | 0.99 | 0.73 | 1.33 | 0.944 | 0.973 |
| rs1650697 | *MSH3* | 1.03 | 0.79 | 1.34 | 0.831 | 0.989 | 0.95 | 0.66 | 1.35 | 0.762 | 0.964 | 1.06 | 0.75 | 1.50 | 0.747 | 0.973 |
| rs174538 | *FEN1* | 0.98 | 0.76 | 1.26 | 0.865 | 0.989 | 0.95 | 0.67 | 1.35 | 0.771 | 0.964 | 1.32 | 0.93 | 1.87 | 0.116 | 0.672 |
| rs175080 | *MLH3* | 0.98 | 0.78 | 1.23 | 0.828 | 0.989 | 0.88 | 0.64 | 1.2 | 0.423 | 0.924 | 1.02 | 0.76 | 1.39 | 0.884 | 0.973 |
| rs1760944 | *APEX1* | 0.88 | 0.68 | 1.13 | 0.315 | 0.869 | 0.96 | 0.68 | 1.34 | 0.804 | 0.964 | 0.78 | 0.56 | 1.08 | 0.131 | 0.672 |
| rs17655 | *ERCC5* | 1.13 | 0.87 | 1.46 | 0.360 | 0.869 | 1.23 | 0.86 | 1.74 | 0.253 | 0.924 | 0.82 | 0.59 | 1.14 | 0.239 | 0.739 |
| rs176641 | *POLG* | 1.06 | 0.83 | 1.35 | 0.634 | 0.989 | 0.86 | 0.62 | 1.20 | 0.382 | 0.924 | 1.14 | 0.83 | 1.57 | 0.421 | 0.885 |
| rs1776148 | *EXO1* | 1.05 | 0.83 | 1.32 | 0.713 | 0.989 | 0.90 | 0.65 | 1.24 | 0.512 | 0.940 | 0.94 | 0.69 | 1.28 | 0.693 | 0.968 |
| rs1799793 | *ERCC2* | 0.94 | 0.73 | 1.20 | 0.603 | 0.989 | 1.01 | 0.72 | 1.42 | 0.946 | 0.964 | 0.85 | 0.62 | 1.18 | 0.328 | 0.805 |
| rs1799794 | *XRCC3* | 0.95 | 0.73 | 1.24 | 0.725 | 0.989 | 1.16 | 0.80 | 1.68 | 0.426 | 0.924 | 1.12 | 0.78 | 1.59 | 0.544 | 0.910 |
| rs1799796 | *XRCC3* | 0.80 | 0.61 | 1.05 | 0.108 | 0.583 | 1.21 | 0.81 | 1.81 | 0.352 | 0.924 | 1.10 | 0.75 | 1.61 | 0.635 | 0.939 |
| rs1799801 | *ERCC4* | 0.99 | 0.77 | 1.27 | 0.933 | 0.989 | 0.94 | 0.68 | 1.31 | 0.723 | 0.964 | 1.28 | 0.92 | 1.78 | 0.137 | 0.672 |
| rs1799955 | *BRCA2* | 0.73 | 0.55 | 0.97 | **0.030** | 0.539 | 0.98 | 0.65 | 1.46 | 0.907 | 0.964 | 1.23 | 0.82 | 1.83 | 0.316 | 0.798 |
| rs1799966 | *BRCA1* | 1.05 | 0.82 | 1.34 | 0.712 | 0.989 | 1.12 | 0.80 | 1.56 | 0.526 | 0.940 | 0.99 | 0.71 | 1.36 | 0.932 | 0.973 |
| rs1799977 | *MLH1* | 0.96 | 0.75 | 1.22 | 0.725 | 0.989 | 0.79 | 0.57 | 1.09 | 0.158 | 0.924 | 0.82 | 0.60 | 1.12 | 0.203 | 0.708 |
| rs1800067 | *ERCC4* | 1.31 | 0.91 | 1.91 | 0.146 | 0.685 | 0.89 | 0.56 | 1.41 | 0.613 | 0.945 | 1.37 | 0.85 | 2.21 | 0.189 | 0.708 |
| rs1800389 | *WRN* | 1.01 | 0.78 | 1.30 | 0.947 | 0.989 | 0.96 | 0.68 | 1.35 | 0.808 | 0.964 | 1.17 | 0.83 | 1.64 | 0.370 | 0.867 |
| rs1800734 | *MLH1* | 0.92 | 0.70 | 1.19 | 0.522 | 0.989 | 1.21 | 0.83 | 1.76 | 0.319 | 0.924 | 1.21 | 0.85 | 1.74 | 0.289 | 0.794 |
| rs1800935 | *MSH6* | 1.13 | 0.87 | 1.48 | 0.348 | 0.869 | 0.92 | 0.65 | 1.31 | 0.655 | 0.964 | 0.75 | 0.53 | 1.05 | 0.099 | 0.672 |
| rs1800975 | *XPA* | 1.06 | 0.83 | 1.36 | 0.618 | 0.989 | 1.10 | 0.79 | 1.54 | 0.579 | 0.940 | 0.87 | 0.63 | 1.19 | 0.386 | 0.885 |
| rs1801406 | *BRCA2* | 0.77 | 0.60 | 1 | **0.048** | 0.583 | 0.97 | 0.68 | 1.39 | 0.874 | 0.964 | 1.04 | 0.74 | 1.47 | 0.830 | 0.973 |
| rs1801516 | *ATM* | 1 | 0.71 | 1.41 | 0.985 | 0.994 | 1.62 | 0.98 | 2.67 | 0.153 | 0.924 | 1.77 | 1.09 | 2.87 | **0.017** | 0.614 |
| rs1802904 | *ATR* | 1.25 | 0.86 | 1.80 | 0.235 | 0.869 | 1.24 | 0.76 | 2.02 | 0.383 | 0.924 | 0.99 | 0.63 | 1.56 | 0.971 | 0.973 |
| rs1805386 | *LIG4* | 1.30 | 0.95 | 1.80 | 0.102 | 0.583 | 1.18 | 0.77 | 1.79 | 0.441 | 0.924 | 0.96 | 0.76 | 1.22 | 0.751 | 0.973 |
| rs1805388 | *LIG4* | 0.99 | 0.70 | 1.39 | 0.937 | 0.989 | 1.26 | 0.77 | 2.05 | 0.357 | 0.924 | 0.84 | 0.54 | 1.32 | 0.451 | 0.885 |
| rs1805794 | *NBS1* | 1.11 | 0.87 | 1.43 | 0.394 | 0.869 | 1.02 | 0.73 | 1.44 | 0.892 | 0.964 | 1.01 | 0.73 | 1.40 | 0.945 | 0.973 |
| rs1981928 | *MSH2* | 0.87 | 0.67 | 1.13 | 0.295 | 0.869 | 0.87 | 0.62 | 1.24 | 0.444 | 0.924 | 0.77 | 0.55 | 1.08 | 0.128 | 0.672 |
| rs2020911 | *MSH6* | 0.92 | 0.72 | 1.16 | 0.482 | 0.989 | 0.83 | 0.6 | 1.15 | 0.265 | 0.924 | 0.94 | 0.68 | 1.28 | 0.678 | 0.963 |
| rs2040639 | *XRCC2* | 1.06 | 0.84 | 1.33 | 0.614 | 0.989 | 0.94 | 0.69 | 1.28 | 0.696 | 0.964 | 0.97 | 0.72 | 1.31 | 0.845 | 0.973 |
| rs2048718 | *BRIP1* | 1.16 | 0.92 | 1.48 | 0.213 | 0.869 | 0.69 | 0.49 | 0.95 | **0.023** | 0.924 | 0.73 | 0.53 | 1.01 | 0.054 | 0.672 |
| rs20580 | *LIG1* | 1.34 | 1.07 | 1.69 | **0.011** | 0.420 | 1.01 | 0.74 | 1.39 | 0.940 | 0.964 | 1.26 | 0.92 | 1.71 | 0.147 | 0.687 |
| rs2074522 | *LIG3* | 1.10 | 0.71 | 1.72 | 0.752 | 0.989 | 1.30 | 0.70 | 2.40 | 0.825 | 0.964 | 1.22 | 0.68 | 2.19 | 0.494 | 0.909 |
| rs2075685 | *XRCC4* | 1.22 | 0.97 | 1.55 | 0.090 | 0.583 | 0.92 | 0.67 | 1.26 | 0.584 | 0.940 | 1.13 | 0.83 | 1.54 | 0.426 | 0.885 |
| rs207906 | *XRCC5* | 1.03 | 0.73 | 1.45 | 0.882 | 0.989 | 1.27 | 0.78 | 2.08 | 0.327 | 0.924 | 1.06 | 0.67 | 1.67 | 0.799 | 0.973 |
| rs2228000 | *XPC* | 0.78 | 0.60 | 1 | **0.048** | 0.583 | 1.07 | 0.75 | 1.51 | 0.721 | 0.964 | 0.91 | 0.65 | 1.27 | 0.583 | 0.925 |
| rs2228001 | *XPC* | 1.03 | 0.81 | 1.30 | 0.823 | 0.989 | 1.22 | 0.88 | 1.69 | 0.226 | 0.924 | 1.03 | 0.76 | 1.41 | 0.837 | 0.973 |
| rs2228006 | *PMS2* | 1.04 | 0.76 | 1.42 | 0.802 | 0.989 | 1.25 | 0.81 | 1.94 | 0.307 | 0.924 | 1.06 | 0.70 | 1.59 | 0.787 | 0.973 |
| rs2238463 | *ERCC4* | 1.05 | 0.82 | 1.34 | 0.696 | 0.989 | 1.02 | 0.73 | 1.42 | 0.912 | 0.964 | 1.36 | 0.99 | 1.89 | 0.059 | 0.672 |
| rs2252775 | *RAD50* | 0.88 | 0.66 | 1.19 | 0.414 | 0.885 | 0.93 | 0.62 | 1.39 | 0.733 | 0.964 | 1.14 | 0.77 | 1.70 | 0.505 | 0.909 |
| rs2272615 | *POLB* | 1.03 | 0.73 | 1.47 | 0.851 | 0.989 | 1.23 | 0.76 | 2.01 | 0.395 | 0.924 | 1.27 | 0.79 | 2.04 | 0.318 | 0.798 |
| rs2286940 | *MLH1* | 1.01 | 0.81 | 1.26 | 0.940 | 0.989 | 0.83 | 0.61 | 1.13 | 0.233 | 0.924 | 0.89 | 0.66 | 1.19 | 0.436 | 0.885 |
| rs2303428 | *MSH2* | 0.96 | 0.66 | 1.40 | 0.826 | 0.989 | 0.72 | 0.44 | 1.18 | 0.199 | 0.924 | 0.88 | 0.54 | 1.45 | 0.626 | 0.939 |
| rs2308321 | *MGMT* | 0.89 | 0.66 | 1.21 | 0.463 | 0.885 | 1.30 | 0.72 | 2.35 | 0.369 | 0.924 | 0.84 | 0.50 | 1.43 | 0.530 | 0.909 |
| rs2345060 | *PMS2* | 1.22 | 0.93 | 1.6 | 0.141 | 0.685 | 0.89 | 0.62 | 1.28 | 0.535 | 0.940 | 1.11 | 0.78 | 1.58 | 0.565 | 0.910 |
| rs2348244 | *MSH6* | 0.91 | 0.65 | 1.27 | 0.584 | 0.989 | 1.16 | 0.72 | 1.86 | 0.541 | 0.940 | 1.54 | 0.95 | 2.48 | 0.069 | 0.672 |
| rs238406 | *ERCC2* | 1.21 | 0.97 | 1.52 | 0.091 | 0.583 | 0.87 | 0.64 | 1.17 | 0.355 | 0.924 | 0.94 | 0.70 | 1.27 | 0.704 | 0.968 |
| rs2434470 | *ALKBH3* | 0.94 | 0.72 | 1.23 | 0.645 | 0.989 | 1.40 | 0.95 | 2.06 | 0.086 | 0.924 | 0.95 | 0.67 | 1.36 | 0.782 | 0.973 |
| rs2440 | *XRCC5* | 0.95 | 0.76 | 1.21 | 0.695 | 0.989 | 1.03 | 0.75 | 1.42 | 0.837 | 0.964 | 1.08 | 0.80 | 1.47 | 0.607 | 0.939 |
| rs25487 | *XRCC1* | 1.11 | 0.88 | 1.39 | 0.394 | 0.869 | 1.13 | 0.82 | 1.55 | 0.454 | 0.924 | 0.74 | 0.55 | 1.01 | 0.054 | 0.672 |
| rs26279 | *MSH3* | 1.02 | 0.80 | 1.30 | 0.875 | 0.989 | 1.20 | 0.86 | 1.67 | 0.292 | 0.924 | 1.01 | 0.74 | 1.39 | 0.933 | 0.973 |
| rs26779 | *MSH3* | 0.88 | 0.69 | 1.12 | 0.285 | 0.869 | 0.93 | 0.67 | 1.30 | 0.676 | 0.964 | 0.98 | 0.71 | 1.34 | 0.876 | 0.973 |
| rs293794 | *OGG1* | 0.87 | 0.65 | 1.18 | 0.388 | 0.869 | 1.22 | 0.79 | 1.87 | 0.363 | 0.924 | 1.01 | 0.68 | 1.51 | 0.967 | 0.973 |
| rs3136038 | *ERCC4* | 1.07 | 0.84 | 1.36 | 0.604 | 0.989 | 1.04 | 0.74 | 1.44 | 0.835 | 0.964 | 1.37 | 0.99 | 1.90 | 0.056 | 0.672 |
| rs3136228 | *MSH6* | 1.09 | 0.85 | 1.39 | 0.515 | 0.989 | 0.96 | 0.69 | 1.35 | 0.824 | 0.964 | 0.74 | 0.53 | 1.02 | 0.067 | 0.672 |
| rs3212948 | *ERCC1* | 0.80 | 0.63 | 1.01 | 0.061 | 0.583 | 0.91 | 0.66 | 1.25 | 0.555 | 0.940 | 0.85 | 0.62 | 1.16 | 0.294 | 0.794 |
| rs3212961 | *ERCC1* | 0.80 | 0.56 | 1.15 | 0.232 | 0.869 | 1.15 | 0.67 | 1.96 | 0.946 | 0.964 | 1.33 | 0.78 | 2.26 | 0.518 | 0.909 |
| rs3212986 | *ERCC1* | 0.89 | 0.69 | 1.16 | 0.391 | 0.869 | 0.87 | 0.61 | 1.26 | 0.472 | 0.940 | 0.76 | 0.54 | 1.08 | 0.133 | 0.672 |
| rs3213245 | *XRCC1* | 1 | 0.79 | 1.27 | 0.969 | 0.989 | 0.94 | 0.68 | 1.28 | 0.685 | 0.964 | 1.21 | 0.89 | 1.64 | 0.226 | 0.739 |
| rs3218536 | *XRCC2* | 1.01 | 0.68 | 1.50 | 0.961 | 0.989 | 1.18 | 0.67 | 2.07 | 0.843 | 0.964 | 1.36 | 0.78 | 2.36 | 0.610 | 0.939 |
| rs3219489 | *MUTYH* | 0.99 | 0.76 | 1.29 | 0.945 | 0.989 | 0.95 | 0.66 | 1.37 | 0.801 | 0.964 | 1.03 | 0.73 | 1.47 | 0.863 | 0.973 |
| rs3626 | *PCNA* | 0.91 | 0.65 | 1.28 | 0.590 | 0.989 | 1.61 | 0.95 | 2.71 | 0.150 | 0.924 | 1.37 | 0.85 | 2.22 | 0.193 | 0.708 |
| rs3730668 | *POLI* | 1.08 | 0.85 | 1.38 | 0.524 | 0.989 | 1.42 | 1 | 2.01 | **0.047** | 0.924 | 1.14 | 0.82 | 1.59 | 0.446 | 0.885 |
| rs3793784 | *ERCC6* | 1.22 | 0.96 | 1.53 | 0.097 | 0.583 | 0.85 | 0.62 | 1.17 | 0.330 | 0.924 | 0.99 | 0.73 | 1.34 | 0.934 | 0.973 |
| rs4150416 | *ERCC3* | 1.26 | 0.98 | 1.62 | 0.075 | 0.583 | 1.08 | 0.76 | 1.52 | 0.670 | 0.964 | 0.79 | 0.57 | 1.09 | 0.153 | 0.687 |
| rs4150441 | *ERCC3* | 0.98 | 0.77 | 1.25 | 0.885 | 0.989 | 1.02 | 0.75 | 1.40 | 0.884 | 0.964 | 1.14 | 0.84 | 1.55 | 0.406 | 0.885 |
| rs4150474 | *ERCC3* | 1.18 | 0.9 | 1.54 | 0.236 | 0.869 | 1.02 | 0.71 | 1.47 | 0.899 | 0.964 | 0.79 | 0.56 | 1.12 | 0.194 | 0.708 |
| rs4234259 | *MLH1* | 1 | 0.80 | 1.25 | 0.970 | 0.989 | 0.84 | 0.62 | 1.14 | 0.269 | 0.924 | 0.91 | 0.68 | 1.21 | 0.506 | 0.909 |
| rs4253160 | *ERCC6* | 1.23 | 0.98 | 1.55 | 0.077 | 0.583 | 0.87 | 0.63 | 1.19 | 0.369 | 0.924 | 1.02 | 0.75 | 1.38 | 0.902 | 0.973 |
| rs4968451 | *BRIP1* | 1.36 | 0.99 | 1.87 | 0.052 | 0.583 | 0.77 | 0.52 | 1.14 | 0.194 | 0.924 | 0.92 | 0.62 | 1.35 | 0.658 | 0.961 |
| rs4986764 | *BRIP1* | 1.01 | 0.81 | 1.27 | 0.920 | 0.989 | 1.16 | 0.84 | 1.59 | 0.365 | 0.924 | 0.97 | 0.71 | 1.31 | 0.826 | 0.973 |
| rs4987876 | *ATM* | 1.52 | 1.01 | 2.28 | **0.041** | 0.583 | 0.83 | 0.51 | 1.33 | 0.436 | 0.924 | 0.96 | 0.60 | 1.54 | 0.864 | 0.973 |
| rs569143 | *MRE11A* | 0.98 | 0.78 | 1.23 | 0.835 | 0.989 | 1.09 | 0.79 | 1.49 | 0.601 | 0.941 | 0.91 | 0.67 | 1.23 | 0.523 | 0.909 |
| rs5744934 | *POLE* | 1.17 | 0.85 | 1.61 | 0.327 | 0.869 | 1 | 0.66 | 1.52 | 0.990 | 0.990 | 0.94 | 0.63 | 1.41 | 0.765 | 0.973 |
| rs601341 | *MRE11A* | 0.89 | 0.70 | 1.13 | 0.326 | 0.869 | 0.98 | 0.71 | 1.36 | 0.905 | 0.964 | 1.12 | 0.81 | 1.53 | 0.491 | 0.909 |
| rs6413436 | *RAD52* | 1.08 | 0.84 | 1.38 | 0.543 | 0.989 | 0.86 | 0.61 | 1.2 | 0.363 | 0.924 | 0.95 | 0.69 | 1.32 | 0.780 | 0.973 |
| rs664143 | *ATM* | 1 | 0.78 | 1.26 | 0.971 | 0.989 | 0.96 | 0.69 | 1.34 | 0.825 | 0.964 | 0.95 | 0.69 | 1.31 | 0.750 | 0.973 |
| rs7182283 | *NEIL1* | 0.89 | 0.71 | 1.12 | 0.313 | 0.869 | 0.76 | 0.56 | 1.04 | 0.090 | 0.924 | 0.85 | 0.63 | 1.16 | 0.308 | 0.798 |
| rs735943 | *EXO1* | 1.11 | 0.89 | 1.39 | 0.358 | 0.869 | 0.89 | 0.65 | 1.20 | 0.429 | 0.924 | 1.29 | 0.96 | 1.74 | 0.085 | 0.672 |
| rs7797466 | *PMS2* | 0.78 | 0.57 | 1.06 | 0.107 | 0.583 | 1.14 | 0.72 | 1.81 | 0.566 | 0.940 | 1.04 | 0.68 | 1.61 | 0.844 | 0.973 |
| rs799917 | *BRCA1* | 1.04 | 0.81 | 1.33 | 0.782 | 0.989 | 1.07 | 0.76 | 1.49 | 0.708 | 0.964 | 0.93 | 0.68 | 1.29 | 0.676 | 0.963 |
| rs8305 | *POLI* | 1.03 | 0.79 | 1.35 | 0.821 | 0.989 | 0.87 | 0.61 | 1.25 | 0.453 | 0.924 | 1.31 | 0.92 | 1.89 | 0.134 | 0.672 |
| rs861528 | *XRCC3* | 1.12 | 0.85 | 1.48 | 0.418 | 0.885 | 0.90 | 0.62 | 1.30 | 0.580 | 0.940 | 0.88 | 0.62 | 1.26 | 0.491 | 0.909 |
| rs861531 | *XRCC3* | 1.15 | 0.91 | 1.47 | 0.240 | 0.869 | 0.81 | 0.59 | 1.11 | 0.181 | 0.924 | 0.81 | 0.60 | 1.10 | 0.186 | 0.708 |
| rs861539 | *XRCC3* | 1.12 | 0.88 | 1.41 | 0.363 | 0.869 | 0.79 | 0.57 | 1.08 | 0.135 | 0.924 | 0.83 | 0.61 | 1.12 | 0.217 | 0.731 |
| rs9350 | *EXO1* | 0.83 | 0.61 | 1.13 | 0.250 | 0.869 | 0.73 | 0.48 | 1.12 | 0.155 | 0.924 | 0.72 | 0.48 | 1.10 | 0.131 | 0.672 |
| rs963248 | *XRCC4* | 0.96 | 0.70 | 1.31 | 0.799 | 0.989 | 0.97 | 0.63 | 1.51 | 0.906 | 0.964 | 0.88 | 0.58 | 1.34 | 0.557 | 0.910 |
| rs9876116 | *MLH1* | 0.99 | 0.79 | 1.24 | 0.954 | 0.989 | 0.80 | 0.59 | 1.08 | 0.139 | 0.924 | 0.93 | 0.70 | 1.25 | 0.629 | 0.939 |
| rs9894946 | *TP53* | 0.96 | 0.71 | 1.31 | 0.807 | 0.989 | 0.83 | 0.55 | 1.25 | 0.379 | 0.924 | 1.02 | 0.68 | 1.54 | 0.906 | 0.973 |

OR, odds ratio; CI, confidence interval.

^a^ORs adjusted by gender, age, smoking habit, and family history of GC.

^b^QFDR-values obtained after applying the False Discovery Rate (FDR) test.

*P*-values <0.05 are highlighted in bold.
