# Supplementary material for: Relevance of DNA repair gene polymorphisms to gastric cancer risk and phenotype
Source: Oncotarget. 2017 Mar 16;8(22):35848–62. doi: 10.18632/oncotarget.16261 (PMC5482622; doi:10.18632/oncotarget.16261)
Supplement: Supplementary file 5 [file oncotarget-08-35848-s005.doc]

**Supplementary Table 5: Significant SNPs associated with gastric cancer risk. Association analysis stratified by anatomical location of the tumor.**

| **CARDIA GC** | | **Univariate analysis** | | | | **Multivariate analysis*** | | | |
| --- | --- | --- | --- | --- | --- | --- | --- | --- | --- |
|  |  | **Codominant** | **Dominant** | **Recessive** | **Log-additive** | **Codominant** | **Dominant** | **Recessive** | **Log-Additive** |
| **Gen** | **db SNP ID** | **OR (95% CI) *P* value** | **OR (95% CI) *P* value** | **OR (95% CI) *P* value** | **OR (95% CI) *P* value** | **OR (95% CI) *P* value** | **OR (95% CI) *P* value** | **OR (95% CI) *P* value** | **OR (95% CI)  *P* value** |
| *RAD52* | rs11226 | 1.81 (0.99-3.30) 0.112 | 1.58 (0.99-2.58) 0.059 | 1.40 (0.84-2.34) 0.202 | 1.35 (1.01-1.82) **0.042** | 1.65 (0.88-3.09) 0.143 | 1.59 (0.99-2.57) **0.049** | 1.25 (0.73-2.14) 0.416 | 1.31 (0.97-1.77) 0.081 |
| *ERCC4* | rs1799801 | 0.74 (0.37-1.47) 0.508 | 0.57 (0.38-0.85) **0.005** | 0.95 (0.48-1.87) 0.855 | 0.70 (0.51-0.97) **0.027** | 0.66 (0.31-1.42) 0.289 | 0.58 (0.38-0.88) **0.010** | 0.84 (0.40-1.77) 0.640 | 0.69 (0.49-0.97) **0.028** |
| *MLH1* | rs1799977 | 1.64 (0.89-3.01) 0.128 | 1.52 (1.01-2.30) **0.042** | 1.32 (0.76-2.31) 0.339 | 1.32 (1.00-1.76) 0.055 | 1.65 (0.87-3.11) 0.122 | 1.46 (0.95-2.24) 0.079 | 1.38 (0.77-2.47) 0.291 | 1.31 (0.97-1.77) 0.075 |
| *LIG4* | rs1805388 | 1.95 (0.52-7.26) 0.399 | 1.60 (1.03-2.50) **0.041** | 1.72 (0.46-6.35) 0.440 | 1.52 (1.03-2.24) **0.041** | 2.16 (0.56-8.29) 0.261 | 1.58 (0.99-2.51) 0.051 | 1.91 (0.50-7.26) 0.368 | 1.52 (1.01-2.28) **0.048** |
| *BRIP1* | rs2048718 | 0.37 (0.17-0.78) **0.007** | 0.72 (0.47-1.10) 0.130 | 0.40 (0.20-0.82) **0.005** | 0.68 (0.50-0.93) **0.014** | 0.43 (0.20-0.93) **0.031** | 0.71 (0.46-1.11) 0.135 | 0.49 (0.24-0.93) **0.034** | 0.71 (0.51-0.96) **0.033** |
| *ERCC4* | rs2238463 | 0.61 (0.33-1.13) 0.145 | 0.54 (0.37-0.81) **0.002** | 0.85 (0.47-1.52) 0.575 | 0.69 (0.51-0.95) **0.017** | 0.57 (0.29-1.10) 0.096 | 0.54 (0.35-0.81) **0.003** | 0.79 (0.42-1.49) 0.462 | 0.67 (0.49-0.92) **0.012** |
| *ERCC4* | rs3136038 | 0.66 (0.35-1.25) 0.229 | 0.59 (0.39-0.87) **0.008** | 0.88 (0.48-1.61) 0.678 | 0.72 (0.53-0.98) **0.032** | 0.60 (0.30-1.19) 0.143 | 0.57 (0.38-0.87) **0.009** | 0.80 (0.42-1.53) 0.486 | 0.69 (0.50-0.96) **0.023** |
| *POL1* | rs3730668 | 1.53 (0.88-2.64) 0.152 | 0.98 (0.64-1.51) 0.920 | 1.74 (1.08-2.80) **0.025** | 1.20 (0.90-1.60) 0.222 | 1.49 (0.84-2.63) 0.175 | 0.98 (0.63-1.54) 0.938 | 1.68 (1.02-2.74) **0.046** | 1.19 (0.88-1.60) 0.268 |
| *TP53* | rs9894946 | NA | 0.56 (0.34-0.91) **0.015** | NA | 0.54 (0.34-0.86) **0.020** | NA | 0.51 (0.30-0.86) **0.011** | NA | 0.50 (0.30-0.81) **0.002** |
| **NON-CARDIA GC** | | **Univariate analysis** | | | | **Multivariate analysis*** | | | |
|  |  | **Codominant** | **Dominant** | **Recessive** | **Log-additive** | **Codominant** | **Dominant** | **Recessive** | **Log-Additive** |
| **Gen** | **db SNP ID** | **OR (95% CI) *P* value** | **OR (95% CI) *P* value** | **OR (95% CI) *P* value** | **OR (95% CI) *P* value** | **OR (95% CI) *P* value** | **OR (95% CI) *P* value** | **OR (95% CI) *P* value** | **OR (95% CI)  *P* value** |
| *TP53* | rs1042522 | 0.91 (0.56-1.47) 0.715 | 0.67 (0.52-0.85) **0.001** | 1.09 (0.68-1.74) 0.724 | 0.78 (0.64-0.95) **0.013** | 1.01 (0.62-1.66) 0.938 | 0.67 (0.52-0.87) **0.001** | 1.22 (0.76-1.97) 0.416 | 0.80 (0.65-0.98) **0.028** |
| *POLG* | rs176641 | 1.27 (0.86-1.88) 0.232 | 1.42 (1.11-1.82) **0.004** | 1.03 (0.72-1.48) 0.864 | 1.21 (1.02-1.45) **0.032** | 1.27 (0.85-1.89) 0.245 | 1.40 (1.09-1.81) **0.008** | 1.04 (0.72-1.50) 0.848 | 1.21 (1.00-1.46) 0.054 |
| *BRCA2* | rs1801406 | 0.56 (0.34-0.92) **0.021** | 0.96 (0.75-1.22) 0.734 | 0.55 (0.33-0.89) **0.012** | 0.88 (0.72-1.07) 0.191 | 0.58 (0.35-0.97) **0.039** | 0.96 (0.75-1.23) 0.761 | 0.57 (0.34-0.94) **0.023** | 0.89 (0.73-1.08) 0.235 |
| *LIG3* | rs2074522 | 7.7 (1.73-34.90) **0.002** | 1.32 (0.961.82) 0.083 | 7.57 (1.69-34.97) **0.001** | 1.42 (1.06-1.89) **0.017** | 8.20 (1.81-37.14) **0.006** | 1.37 (0.99-1.90) 0.057 | 7.94 (1.76-35.91) **0.001** | 1.46 (1.09-1.96) **0.010** |
| *XPC* | rs2228000 | 0.78 (0.51-1.20) 0.281 | 0.72 (0.57-0.91) **0.007** | 0.92 (0.61-1.38) 0.681 | 0.81 (0.67-0.97) **0.024** | 0.81 (0.53-1.26) 0.354 | 0.70 (0.55-0.90) **0.004** | 0.98 (0.64-1.48) 0.907 | 0.81 (0.67-0.98) **0.026** |
| *MSH6* | rs2348244 | 0.32 (0.09-1.14) 0.101 | 0.73 (0.55-0.98) **0.032** | 0.33 (0.09-1.21) 0.068 | 0.72 (0.55-0.94) **0.016** | 0.33 (0.09-1.20) 0.092 | 0.74 (0.55-0.99) **0.044** | 0.35 (0.10-1.27) 0.082 | 0.73 (0.56-0.96) **0.023** |
| *MSH3* | rs26779 | 0.78 (0.55-1.13) 0.198 | 0.74 (0.58-0.94) **0.015** | 0.94 (0.67-1.31) 0.714 | 0.85 (0.71-1.01) 0.059 | 0.80 (0.55-1.17) 0.259 | 0.76 (0.59-0.99) **0.039** | 0.94 (0.67-1.33) 0.745 | 0.86 (0.72-1.03) 0.106 |
| *ERCC3* | rs4150416 | 0.77 (0.50-1.18) 0.232 | 0.76 (0.60-0.97) **0.028** | 0.87 (0.57-1.32) 0.506 | 0.83 (0.69-0.99) **0.047** | 0.72 (0.46-1.13) 0.157 | 0.73 (0.57-0.94) **0.013** | 0.84 (0.55-1.29) 0.429 | 0.80 (0.66-0.97) **0.024** |
| *BRIP1* | rs4986764 | 0.60 (0.41-0.87) **0.007** | 0.86 (0.67-1.10) 0.238 | 0.61 (0.43-0.86) **0.004** | 0.82 (0.69-0.97) **0.021** | 0.58 (0.39-0.86) **0.007** | 0.87 (0.67-1.11) 0.249 | 0.59 (0.41-0.85) **0.003** | 0.81 (0.68- 0.97) **0.022** |
| *XRCC3* | rs861528 | 1.31 (0.77-2.25) 0.332 | 1.37 (1.06-1.76) **0.014** | 1.15 (0.68-1.95) 0.596 | 1.26 (1.03-1.55) **0.027** | 1.41 (0.82-2.44) 0.218 | 1.45 (1.11-1.88) **0.010** | 1.21 (0.71-2.06) 0.494 | 1.31 (1.06-1.62) **0.011** |
| *TP53* | rs9894946 | 0.38 (0.15-0.96) **0.038** | 0.75 (0.57-0.98) **0.036** | 0.40 (0.16-1.02) 0.060 | 0.74 (0.58-0.94) **0.014** | 0.27 (0.09-0.82) **0.021** | 0.74 (0.56-0.98) **0.033** | 0.29 (0.10-0.87) **0.014** | 0.72 (0.56-0.93) **0.009** |

OR, odds ratio; 95% CI, 95% confidence interval; NA, not applied.

ORs and 95% CI in the multivariate analysis were adjusted for age, gender, *H. pylori* infection, smoking habit, and family history of GC.

*P* values < 0.05 are highlighted in bold.
