# Supplementary material for: Relevance of DNA repair gene polymorphisms to gastric cancer risk and phenotype
Source: Oncotarget. 2017 Mar 16;8(22):35848–62. doi: 10.18632/oncotarget.16261 (PMC5482622; doi:10.18632/oncotarget.16261)
Supplement: Supplementary file 6 [file oncotarget-08-35848-s006.doc]

**Supplementary Table 6: Significant SNPs associated with gastric cancer risk. Association analysis stratified by histological tumor type.**

| **INTESTINAL GC** | | **Univariate analysis** | | | | **Multivariate analysis*** | | | |
| --- | --- | --- | --- | --- | --- | --- | --- | --- | --- |
|  |  | **Codominant** | **Dominant** | **Recessive** | **Log-additive** | **Codominant** | **Dominant** | **Recessive** | **Log-Additive** |
| **Gen** | **db SNP ID** | **OR (95% CI) *P* value** | **OR (95% CI) *P* value** | **OR (95% CI) *P* value** | **OR (95% CI) *P* value** | **OR (95% CI) *P* value** | **OR (95% CI) *P* value** | **OR (95% CI) *P* value** | **OR (95% CI)  *P* value** |
| *TP53* | rs1042522 | 1.05 (0.59-1.85) 0.884 | 0.73 (0.54-0.98) **0.038** | 0.82 (0.47-1.43) 0.466 | 0.85 (0.66-1.08) 0.170 | 1.08 (0.60-1.95) 0.793 | 0.72 (0.53-0.98) **0.037** | 0.89 (0.71-2.26) 0.431 | 0.84 (0.66-1.08) 0.180 |
| *ERCC5* | rs17655 | 0.74 (0.42-1.32) 0.332 | 0.65 (0.48-0.88) **0.005** | 0.88 (0.50-1.55) 0.657 | 0.75 (0.59-0.95) **0.017** | 0.62 (0.33-1.16) 0.136 | 0.60 (0.43-0.82) **0.001** | 0.75 (0.40-1.39) 0.351 | 0.69 (0.53-0.89) **0.003** |
| *POLG* | rs176641 | 1.23 (0.76-2.00) 0.448 | 1.41 (1.04-1.91) **0.027** | 1.00 (0.64-1.57) 0.996 | 1.19 (0.96-1.49) 0.110 | 1.25 (0.75-2.06) 0.385 | 1.40 (1.02-1.93) **0.035** | 1.02 (0.64-1.62) 0.936 | 1.20 (0.96-1.50) 0.117 |
| *LIG3* | rs2074522 | 6.15 (1.1-31.94) **0.025** | 1.16 (0.78-1.73) 0.461 | 6.10 (1.1-31.66) **0.021** | 1.26 (0.88-1.81) 0.217 | 6.08 (1.1-32.23) **0.032** | 1.21 (0.80-1.82) 0.371 | 6.0 (1.14-31.53) 0.023 | 1.30 (0.90-1.89) 0.168 |
| *XPC* | rs2228000 | 0.74 (0.44-1.26) 0.299 | 0.69 (0.51-0.92) **0.013** | 0.89 (0.53-1.49) 0.653 | 0.78 (0.62-0.98) **0.034** | 0.78 (0.45-1.35) 0.374 | 0.69 (0.51-0.94) **0.017** | 0.94 (0.55-1.59) 0.808 | 0.79 (0.62-1.01) 0.052 |
| *MSH6* | rs2348244 | 0.40 (0.09-1.84) 0.363 | 0.69 (0.48-0.99) **0.039** | 0.43 (0.10-1.97) 0.237 | 0.69 (0.50-0.97) **0.029** | 0.43 (0.09-2.00) 0.284 | 0.74 (0.51-1.07) 0.106 | 0.46 (0.10-2.12) 0.279 | 0.74 (0.53-1.04) 0.080 |
| *ERCC3* | rs4150416 | 0.83 (0.49-1.39) 0.520 | 0.72 (0.54-0.97) **0.032** | 0.98 (0.59-1.61) 0.922 | 0.82 (0.65-1.03) 0.092 | 0.77 (0.45-1.32) 0.343 | 0.68 (0.50-0.92) **0.013** | 0.94 (0.56-1.57) 0.815 | 0.79 (0.62-1.00) 0.053 |
| *BRIP1* | rs4986764 | 0.56 (0.34-0.93) **0.025** | 0.99 (0.73-1.34) 0.950 | 0.52 (0.33-0.82) **0.003** | 0.85 (0.68-1.05) 0.127 | 0.53 (0.30-0.87) **0.013** | 0.95 (0.69-1.31) 0.751 | 0.48 (0.29-0.78) **0.001** | 0.82 (0.65-1.02) 0.074 |
| *EXO1* | rs735943 | 0.69 (0.44-1.05) 0.104 | 0.94 (0.68-1.30) 0.723 | 0.65 (0.45-0.95) **0.022** | 0.85 (0.69-1.05) 0.124 | 0.68 (0.44-1.07) 0.097 | 0.93 (0.66-1.30) 0.672 | 0.66 (0.45-0.97) **0.032** | 0.85 (0.68-1.05) 0.133 |
| *TP53* | rs9894946 | 0.48 (0.16-1.45) 0.242 | 0.71 (0.51-1.00) **0.047** | 0.52 (0.181.56) 0.216 | 0.73 (0.54-0.98) **0.034** | 0.37 (0.11-1.29) 0.119 | 0.71 (0.50-1.02) 0.057 | 0.40 (0.12-1.38) 0.111 | 0.71 (0.52-0.98) **0.032** |
| **DIFFUSE GC** | | **Univariate analysis** | | | | **Multivariate analysis*** | | | |
|  |  | **Codominant** | **Dominant** | **Recessive** | **Log-additive** | **Codominant** | **Dominant** | **Recessive** | **Log-Additive** |
| **Gen** | **db SNP ID** | **OR (95% CI) *P* value** | **OR (95% CI) *P* value** | **OR (95% CI) *P* value** | **OR (95% CI) *P* value** | **OR (95% CI) *P* value** | **OR (95% CI) *P* value** | **OR (95% CI) *P* value** | **OR (95% CI)  *P* value** |
| *TP53* | rs1042522 | 0.83 (0.42-1.65) 0.738 | 0.63 (0.45-0.89) **0.007** | 0.92 (0.57-1.47) 0.730 | 0.74 (0.56-0.98) **0.032** | 1.05 (0.52-2.12) 0.881 | 0.64 (0.44-0.92) **0.015** | 1.01 (0.52-1.98) 0.967 | 0.77 (0.57-1.05) 0.092 |
| *RAD52* | rs11226 | 1.73 (1.03-2.89) **0.041** | 1.49 (1.01-2.21) **0.041** | 1.40 (0.90-2.18) 0.145 | 1.32 (1.03-1.70) **0.030** | 1.72 (0.99-2.98) 0.052 | 1.36 (0.90-2.05) 0.141 | 1.51 (0.94-2.44) 0.095 | 1.30 (0.99-1.71) 0.055 |
| *APEX1* | rs1130409 | 0.66 (0.41-1.04) 0.082 | 0.65 (0.46-0.93) **0.020** | 0.85 (0.57-1.28) 0.432 | 0.79 (0.63-1.00) 0.052 | 0.57 (0.35-0.95) **0.030** | 0.61 (0.41-0.89) **0.011** | 0.76 (0.49-1.19) 0.222 | 0.74 (0.58-0.96) **0.019** |
| *POLG* | rs176641 | 0.96 (0.53-1.74) 1.00 | 1.44 (1.02-2.04) **0.038** | 0.74 (0.43-1.30) 0.283 | 1.14 (0.89-1.46) 0.314 | 0.96 (0.51-1.81) 0.891 | 1.41 (0.97-2.04) 0.069 | 0.75 (0.42-1.37) 0.342 | 1.13 (0.86-1.47) 0.374 |
| *XRCC3* | rs1799794 | 0.32 (0.11-0.91) **0.023** | 0.95 (0.68-1.33) 0.679 | 0.31 (0.11-0.88) **0.010** | 0.85 (0.64-1.13) 0.248 | 0.34 (0.12-0.99) **0.047** | 0.88 (0.61-1.26) 0.481 | 0.34 (0.12-0.98) **0.023** | 0.81 (0.59-1.09) 0.163 |
| *XRCC3* | rs861528 | 2.09 (1.04-4.18) **0.040** | 1.92 (1.34-2.75) **0.0003** | 1.57 (0.81-3.07) 0.197 | 1.62 (1.23-2.14) **0.0007** | 2.45 (1.18-5.07) **0.016** | 2.11 (1.43-3.12) **0.0001** | 1.75 (0.87-3.51) 0.128 | 1.75 (1.30-2.37) **0.0002** |
| *XRCC3* | rs861531 | 1.62 (0.97-2.70) 0.076 | 1.60 (1.11-2.31) **0.010** | 1.22 (0.78-1.92) 0.384 | 1.32 (1.03-1.68) **0.026** | 1.76 (1.01-3.09) **0.047** | 1.89 (1.26-2.84) **0.001** | 1.17 (0.72-1.89) 0.536 | 1.39 (1.07-1.81) **0.012** |

OR, odds ratio; 95% CI, 95% confidence interval.

ORs and 95% CI in the multivariate analysis were adjusted for age, gender, *H. pylori* infection, smoking habit, and family history of GC.

*P* values < 0.05 are highlighted in bold.
