# Supplementary material for: Relevance of DNA repair gene polymorphisms to gastric cancer risk and phenotype
Source: Oncotarget. 2017 Mar 16;8(22):35848–62. doi: 10.18632/oncotarget.16261 (PMC5482622; doi:10.18632/oncotarget.16261)
Supplement: Supplementary file 7 [file oncotarget-08-35848-s007.doc]

**Supplementary Table 7: Association of DNA repair gene polymorphisms with GC risk. Stratified analysis by age.**

| **Age < 50 years** | | | | | | | **Age > 50 years** | | | | |
| --- | --- | --- | --- | --- | --- | --- | --- | --- | --- | --- | --- |
|  |  | **Log-additive genetic model** | | | | | **Log-additive genetic model** | | | | |
|  |  |  | **95% CI** | |  |  |  | **95% CI** | |  |  |
| **db SNP ID** | **Gen** | **OR^a^** | **Lower** | **Upper** | ***P-*value** | **FDR^b^** | **OR^a^** | **Lower** | **Upper** | ***P*-value** | **FDR^b^** |
| rs10079641 | *MSH3* | 1.06 | 0.43 | 2.65 | 0.900 | 0.989 | 0.97 | 0.74 | 1.29 | 0.845 | 0.983 |
| rs1042522 | *TP53* | 0.91 | 0.45 | 1.84 | 0.797 | 0.942 | 0.77 | 0.63 | 0.93 | **0.006** | 0.334 |
| rs1047768 | *ERCC5* | 0.76 | 0.44 | 1.33 | 0.334 | 0.796 | 0.95 | 0.80 | 1.12 | 0.531 | 0.828 |
| rs1047840 | *EXO1* | 1.10 | 0.87 | 1.39 | 0.431 | 0.975 | 0.85 | 0.72 | 1.01 | 0.066 | 0.509 |
| rs1048771 | *RAD54L* | 1.04 | 0.71 | 1.52 | 0.839 | 0.796 | 1.09 | 0.83 | 1.44 | 0.524 | 0.828 |
| rs1051677 | *XRCC5* | 0.75 | 0.33 | 1.71 | 0.317 | 0.796 | 1.18 | 0.87 | 1.60 | 0.276 | 0.766 |
| rs1051685 | *XRCC5* | 0.61 | 0.27 | 1.42 | 0.230 | 0.796 | 1.09 | 0.83 | 1.44 | 0.533 | 0.828 |
| rs1052133 | *OGG1* | 0.98 | 0.53 | 1.82 | 0.951 | 0.989 | 0.93 | 0.76 | 1.15 | 0.507 | 0.828 |
| rs1059262 | *ALKBH2* | 1.08 | 0.51 | 2.27 | 0.849 | 0.975 | 0.90 | 0.73 | 1.11 | 0.319 | 0.771 |
| rs1060915 | *BRCA1* | 1.24 | 0.67 | 2.29 | 0.497 | 0.839 | 1.09 | 0.91 | 1.30 | 0.374 | 0.776 |
| rs11226 | *RAD52* | 1.29 | 0.67 | 2.50 | 0.448 | 0.839 | 1.21 | 1.01 | 1.43 | **0.033** | 0.477 |
| rs1130409 | *APEX1* | 1.04 | 0.57 | 1.74 | 0.998 | 1 | 0.90 | 0.76 | 1.06 | 0.193 | 0.631 |
| rs1136410 | *PARP1* | 1.55 | 0.66 | 3.63 | 0.394 | 0.805 | 1.18 | 0.92 | 1.52 | 0.181 | 0.616 |
| rs13180316 | *XRCC4* | 1.51 | 0.81 | 2.82 | 0.186 | 0.796 | 0.96 | 0.8 | 1.16 | 0.687 | 0.916 |
| rs13181 | *ERCC2* | 0.66 | 0.38 | 1.14 | 0.132 | 0.796 | 1.15 | 0.97 | 1.37 | 0.110 | 0.595 |
| rs1346044 | *WRN* | 0.92 | 0.46 | 1.82 | 0.862 | 0.975 | 0.87 | 0.71 | 1.06 | 0.174 | 0.616 |
| rs144848 | *BRCA2* | 1.02 | 0.56 | 1.85 | 0.944 | 0.989 | 1.10 | 0.91 | 1.33 | 0.310 | 0.771 |
| rs1478485 | *XRCC4* | 0.68 | 0.38 | 1.21 | 0.187 | 0.796 | 1.14 | 0.96 | 1.35 | 0.144 | 0.616 |
| rs1540354 | *MLH1* | 1.40 | 0.55 | 3.57 | 0.654 | 0.855 | 0.88 | 0.68 | 1.13 | 0.320 | 0.771 |
| rs1614984 | *TP53* | 0.65 | 0.36 | 1.19 | 0.159 | 0.796 | 1.19 | 0.99 | 1.42 | 0.057 | 0.477 |
| rs1618536 | *ERCC2* | 0.79 | 0.44 | 1.42 | 0.423 | 0.839 | 0.93 | 0.78 | 1.09 | 0.367 | 0.776 |
| rs1650697 | *MSH3* | 0.96 | 0.51 | 1.80 | 0.904 | 0.989 | 0.91 | 0.74 | 1.10 | 0.321 | 0.771 |
| rs174538 | *FEN1* | 0.99 | 0.51 | 1.92 | 0.977 | 0.996 | 0.99 | 0.83 | 1.20 | 0.953 | 0.983 |
| rs175080 | *MLH3* | 0.86 | 0.49 | 1.50 | 0.585 | 0.845 | 1.04 | 0.88 | 1.23 | 0.628 | 0.871 |
| rs1760944 | *APEX1* | 1.02 | 0.57 | 1.85 | 0.944 | 0.989 | 1.01 | 0.84 | 1.22 | 0.901 | 0.983 |
| rs17655 | *ERCC5* | 1.16 | 0.65 | 2.05 | 0.617 | 0.855 | 0.79 | 0.65 | 0.96 | **0.017** | 0.477 |
| rs176641 | *POLG* | 1.10 | 0.62 | 1.95 | 0.751 | 0.942 | 1.19 | 0.99 | 1.41 | 0.057 | 0.477 |
| rs1776148 | *EXO1* | 1.28 | 0.73 | 2.26 | 0.388 | 0.805 | 0.96 | 0.81 | 1.14 | 0.626 | 0.871 |
| rs1799793 | *ERCC2* | 0.76 | 0.45 | 1.28 | 0.300 | 0.796 | 1.15 | 0.96 | 1.37 | 0.131 | 0.616 |
| rs1799794 | *XRCC3* | 0.79 | 0.41 | 1.50 | 0.465 | 0.839 | 0.94 | 0.78 | 1.14 | 0.556 | 0.828 |
| rs1799796 | *XRCC3* | 0.72 | 0.36 | 1.43 | 0.345 | 0.796 | 0.91 | 0.75 | 1.12 | 0.373 | 0.776 |
| rs1799801 | *ERCC4* | 0.75 | 0.42 | 1.34 | 0.325 | 0.796 | 0.92 | 0.77 | 1.10 | 0.343 | 0.776 |
| rs1799955 | *BRCA2* | 0.92 | 0.47 | 1.80 | 0.799 | 0.942 | 0.98 | 0.79 | 1.20 | 0.827 | 0.983 |
| rs1799966 | *BRCA1* | 1.24 | 0.67 | 2.29 | 0.497 | 0.839 | 1.05 | 0.88 | 1.26 | 0.609 | 0.871 |
| rs1799977 | *MLH1* | 0.69 | 0.38 | 1.24 | 0.206 | 0.796 | 1.01 | 0.85 | 1.21 | 0.903 | 0.983 |
| rs1800067 | *ERCC4* | 0.62 | 0.24 | 1.61 | 0.522 | 0.845 | 0.97 | 0.75 | 1.27 | 0.840 | 0.983 |
| rs1800389 | *WRN* | 0.87 | 0.45 | 1.67 | 0.740 | 0.942 | 0.88 | 0.73 | 1.05 | 0.161 | 0.616 |
| rs1800734 | *MLH1* | 1.39 | 0.74 | 2.62 | 0.301 | 0.796 | 0.99 | 0.81 | 1.19 | 0.881 | 0.983 |
| rs1800935 | *MSH6* | 0.97 | 0.53 | 1.78 | 0.921 | 0.989 | 1.15 | 0.95 | 1.39 | 0.159 | 0.616 |
| rs1800975 | *XPA* | 0.76 | 0.44 | 1.29 | 0.299 | 0.796 | 0.95 | 0.79 | 1.14 | 0.570 | 0.832 |
| rs1801406 | *BRCA2* | 0.86 | 0.44 | 1.68 | 0.659 | 0.889 | 0.90 | 0.75 | 1.09 | 0.287 | 0.771 |
| rs1801516 | *ATM* | 1.95 | 0.74 | 5.15 | 0.372 | 0.796 | 1.09 | 0.86 | 1.38 | 0.491 | 0.828 |
| rs1802904 | *ATR* | 1.41 | 0.60 | 3.27 | 0.602 | 0.845 | 1.11 | 0.87 | 1.43 | 0.398 | 0.776 |
| rs1805386 | *LIG4* | 0.98 | 0.47 | 2.01 | 0.949 | 0.989 | 0.97 | 0.77 | 1.22 | 0.785 | 0.983 |
| rs1805388 | *LIG4* | 1.45 | 0.63 | 3.35 | 0.402 | 0.839 | 1.09 | 0.85 | 1.4 | 0.512 | 0.828 |
| rs1805794 | *NBS1* | 1.29 | 0.74 | 2.25 | 0.368 | 0.796 | 1.02 | 0.86 | 1.22 | 0.798 | 0.983 |
| rs1981928 | *MSH2* | 0.71 | 0.37 | 1.34 | 0.288 | 0.796 | 1.01 | 0.84 | 1.23 | 0.898 | 0.983 |
| rs2020911 | *MSH6* | 0.93 | 0.51 | 1.68 | 0.802 | 0.942 | 0.98 | 0.82 | 1.16 | 0.801 | 0.983 |
| rs2040639 | *XRCC2* | 1.46 | 0.85 | 2.51 | 0.172 | 0.796 | 0.85 | 0.72 | 1 | 0.053 | 0.477 |
| rs2048718 | *BRIP1* | 1.10 | 0.62 | 1.98 | 0.737 | 0.942 | 0.93 | 0.78 | 1.1 | 0.402 | 0.776 |
| rs20580 | *LIG1* | 1.22 | 0.74 | 2.04 | 0.435 | 0.839 | 1.06 | 0.9 | 1.25 | 0.473 | 0.828 |
| rs2074522 | *LIG3* | 0.98 | 0.34 | 2.72 | 1 | 1 | 1.32 | 0.99 | 1.76 | 0.054 | 0.477 |
| rs2075685 | *XRCC4* | 0.72 | 0.41 | 1.27 | 0.254 | 0.796 | 1.09 | 0.92 | 1.3 | 0.314 | 0.771 |
| rs207906 | *XRCC5* | 0.65 | 0.29 | 1.45 | 0.288 | 0.796 | 1 | 0.77 | 1.28 | 0.974 | 0.983 |
| rs2228000 | *XPC* | 0.70 | 0.38 | 1.29 | 0.251 | 0.796 | 0.83 | 0.69 | 0.99 | **0.040** | 0.477 |
| rs2228001 | *XPC* | 0.91 | 0.51 | 1.63 | 0.757 | 0.942 | 0.99 | 0.84 | 1.18 | 0.953 | 0.983 |
| rs2228006 | *PMS2* | 0.85 | 0.37 | 1.96 | 0.553 | 0.845 | 1.11 | 0.88 | 1.4 | 0.396 | 0.776 |
| rs2238463 | *ERCC4* | 0.66 | 0.36 | 1.22 | 0.180 | 0.796 | 0.84 | 0.71 | 1 | **0.049** | 0.477 |
| rs2252775 | *RAD50* | 1.20 | 0.58 | 2.46 | 0.262 | 0.796 | 1.01 | 0.82 | 1.26 | 0.895 | 0.983 |
| rs2272615 | *POLB* | 0.62 | 0.27 | 1.38 | 0.348 | 0.796 | 1.01 | 0.78 | 1.30 | 0.948 | 0.983 |
| rs2286940 | *MLH1* | 0.81 | 0.46 | 1.44 | 0.478 | 0.839 | 1.04 | 0.88 | 1.23 | 0.642 | 0.878 |
| rs2303428 | *MSH2* | 1.76 | 0.81 | 3.81 | 0.178 | 0.796 | 1.09 | 0.82 | 1.44 | 0.560 | 0.828 |
| rs2308321 | *MGMT* | 0.98 | 0.36 | 2.65 | 0.775 | 0.942 | 0.91 | 0.68 | 1.21 | 0.510 | 0.828 |
| rs2345060 | *PMS2* | 0.65 | 0.34 | 1.23 | 0.177 | 0.796 | 0.86 | 0.71 | 1.05 | 0.146 | 0.616 |
| rs2348244 | *MSH6* | 0.87 | 0.33 | 2.26 | 0.821 | 0.975 | 0.78 | 0.60 | 1.01 | 0.056 | 0.477 |
| rs238406 | *ERCC2* | 1.28 | 0.76 | 2.15 | 0.349 | 0.796 | 1 | 0.85 | 1.18 | 0.968 | 0.983 |
| rs2434470 | *ALKBH3* | 0.94 | 0.46 | 1.89 | 0.858 | 0.975 | 0.86 | 0.70 | 1.04 | 0.126 | 0.616 |
| rs2440 | *XRCC5* | 1.73 | 0.95 | 3.18 | 0.070 | 0.796 | 0.99 | 0.83 | 1.17 | 0.890 | 0.983 |
| rs25487 | *XRCC1* | 1.12 | 0.66 | 1.89 | 0.677 | 0.903 | 0.86 | 0.73 | 1.02 | 0.085 | 0.572 |
| rs26279 | *MSH3* | 1.34 | 0.70 | 2.56 | 0.368 | 0.796 | 1 | 0.84 | 1.19 | 0.989 | 0.989 |
| rs26779 | *MSH3* | 0.87 | 0.50 | 1.49 | 0.603 | 0.845 | 0.86 | 0.72 | 1.02 | 0.081 | 0.572 |
| rs293794 | *OGG1* | 0.46 | 0.21 | 1.02 | 0.119 | 0.796 | 1.09 | 0.87 | 1.36 | 0.469 | 0.828 |
| rs3136038 | *ERCC4* | 0.66 | 0.35 | 1.22 | 0.176 | 0.796 | 0.84 | 0.71 | 1.01 | 0.057 | 0.477 |
| rs3136228 | *MSH6* | 1.17 | 0.68 | 1.99 | 0.574 | 0.845 | 1.08 | 0.91 | 1.29 | 0.387 | 0.776 |
| rs3212948 | *ERCC1* | 0.86 | 0.50 | 1.48 | 0.579 | 0.845 | 1.02 | 0.86 | 1.21 | 0.807 | 0.983 |
| rs3212961 | *ERCC1* | 0.98 | 0.43 | 2.23 | 0.821 | 0.954 | 1.08 | 0.84 | 1.40 | 0.556 | 0.828 |
| rs3212986 | *ERCC1* | 0.81 | 0.43 | 1.52 | 0.505 | 0.839 | 1.01 | 0.83 | 1.23 | 0.906 | 0.983 |
| rs3213245 | *XRCC1* | 0.70 | 0.37 | 1.34 | 0.280 | 0.796 | 1.11 | 0.94 | 1.31 | 0.206 | 0.637 |
| rs3218536 | *XRCC2* | 1.50 | 0.66 | 3.39 | 0.571 | 0.845 | 1.01 | 0.76 | 1.35 | 0.942 | 0.983 |
| rs3219489 | *MUTYH* | 0.69 | 0.36 | 1.35 | 0.277 | 0.796 | 0.92 | 0.76 | 1.12 | 0.398 | 0.776 |
| rs3626 | *PCNA* | 1.55 | 0.66 | 3.64 | 0.570 | 0.845 | 0.89 | 0.69 | 1.15 | 0.372 | 0.776 |
| rs3730668 | *POLI* | 1.22 | 0.70 | 2.13 | 0.476 | 0.839 | 0.96 | 0.81 | 1.14 | 0.665 | 0.898 |
| rs3793784 | *ERCC6* | 0.72 | 0.42 | 1.24 | 0.233 | 0.796 | 0.94 | 0.79 | 1.11 | 0.440 | 0.805 |
| rs4150416 | *ERCC3* | 1.12 | 0.61 | 2.06 | 0.715 | 0.930 | 0.82 | 0.68 | 0.98 | **0.032** | 0.477 |
| rs4150441 | *ERCC3* | 0.73 | 0.41 | 1.28 | 0.270 | 0.796 | 1.12 | 0.94 | 1.33 | 0.207 | 0.637 |
| rs4150474 | *ERCC3* | 0.92 | 0.48 | 1.75 | 0.797 | 0.942 | 0.87 | 0.71 | 1.06 | 0.156 | 0.616 |
| rs4234259 | *MLH1* | 0.77 | 0.43 | 1.38 | 0.376 | 0.796 | 1.03 | 0.87 | 1.21 | 0.735 | 0.968 |
| rs4253160 | *ERCC6* | 0.74 | 0.43 | 1.28 | 0.275 | 0.796 | 0.93 | 0.79 | 1.10 | 0.385 | 0.776 |
| rs4968451 | *BRIP1* | 0.70 | 0.31 | 1.56 | 0.589 | 0.845 | 0.91 | 0.73 | 1.14 | 0.421 | 0.785 |
| rs4986764 | *BRIP1* | 0.64 | 0.35 | 1.19 | 0.156 | 0.796 | 0.87 | 0.73 | 1.03 | 0.102 | 0.577 |
| rs4987876 | *ATM* | 0.26 | 0.07 | 0.93 | **0.049** | 0.796 | 0.97 | 0.73 | 1.28 | 0.822 | 0.983 |
| rs569143 | *MRE11A* | 0.72 | 0.43 | 1.20 | 0.204 | 0.796 | 0.90 | 0.76 | 1.07 | 0.224 | 0.655 |
| rs5744934 | *POLE* | 0.98 | 0.48 | 1.98 | 0.953 | 0.989 | 1.06 | 0.85 | 1.31 | 0.629 | 0.871 |
| rs601341 | *MRE11A* | 0.70 | 0.41 | 1.19 | 0.187 | 0.796 | 0.89 | 0.75 | 1.06 | 0.183 | 0.616 |
| rs6413436 | *RAD52* | 1.18 | 0.64 | 2.19 | 0.601 | 0.845 | 1.14 | 0.96 | 1.36 | 0.135 | 0.616 |
| rs664143 | *ATM* | 0.96 | 0.55 | 1.69 | 0.892 | 0.989 | 0.86 | 0.72 | 1.02 | 0.091 | 0.577 |
| rs7182283 | *NEIL1* | 0.82 | 0.47 | 1.43 | 0.484 | 0.839 | 0.93 | 0.79 | 1.10 | 0.416 | 0.785 |
| rs735943 | *EXO1* | 1.10 | 0.67 | 1.81 | 0.704 | 0.928 | 0.91 | 0.77 | 1.07 | 0.248 | 0.704 |
| rs7797466 | *PMS2* | 2.02 | 0.93 | 4.38 | 0.136 | 0.796 | 1.03 | 0.82 | 1.29 | 0.816 | 0.983 |
| rs799917 | *BRCA1* | 1.16 | 0.62 | 2.17 | 0.633 | 0.866 | 1.06 | 0.88 | 1.26 | 0.554 | 0.828 |
| rs8305 | *POLI* | 1.57 | 0.79 | 3.13 | 0.197 | 0.796 | 1.12 | 0.93 | 1.35 | 0.224 | 0.655 |
| rs861528 | *XRCC3* | 1.86 | 0.97 | 3.54 | 0.055 | 0.796 | 1.15 | 0.94 | 1.42 | 0.172 | 0.616 |
| rs861531 | *XRCC3* | 1.37 | 0.76 | 2.48 | 0.289 | 0.796 | 1.06 | 0.89 | 1.26 | 0.530 | 0.828 |
| rs861539 | *XRCC3* | 1.20 | 0.67 | 2.14 | 0.542 | 0.845 | 1.01 | 0.85 | 1.20 | 0.939 | 0.983 |
| rs9350 | *EXO1* | 2.19 | 1.03 | 4.68 | **0.035** | 0.796 | 0.82 | 0.65 | 1.04 | 0.099 | 0.577 |
| rs963248 | *XRCC4* | 1.22 | 0.57 | 2.57 | 0.242 | 0.796 | 1.01 | 0.81 | 1.27 | 0.908 | 0.983 |
| rs9876116 | *MLH1* | 0.81 | 0.46 | 1.43 | 0.467 | 0.839 | 1 | 0.84 | 1.18 | 0.965 | 0.983 |
| rs9894946 | *TP53* | 1.62 | 0.72 | 3.65 | 0.310 | 0.796 | 0.65 | 0.51 | 0.83 | **0.001** | 0.057 |

GC, gastric cancer; OR, odds ratio; CI, confidence interval.

^a^ORs adjusted by gender, *Helicobacter pylori* infection, smoking, and family history of gastric cancer.

^b^QFDR-values obtained after applying the False Discovery Rate (FDR) test.

*P*-values <0.05 are highlighted in bold.
