# Supplementary material for: Relevance of DNA repair gene polymorphisms to gastric cancer risk and phenotype
Source: Oncotarget. 2017 Mar 16;8(22):35848–62. doi: 10.18632/oncotarget.16261 (PMC5482622; doi:10.18632/oncotarget.16261)
Supplement: Supplementary file 8 [file oncotarget-08-35848-s008.doc]

**Supplementary Table 8: Association of DNA repair polymorphisms with gastric cancer risk. Stratified analysis by gender.**

| **Female Gender** | | | | | | | **Male Gender** | | | | |
| --- | --- | --- | --- | --- | --- | --- | --- | --- | --- | --- | --- |
|  |  | **Log-additive genetic model** | | | | | **Log-additive genetic model** | | | | |
|  |  |  | **95% CI** | |  |  |  | **95% CI** | |  |  |
| **db SNP ID** | **Gen** | **OR^a^** | **Lower** | **Upper** | ***P-*value** | **FDR^b^** | **OR^a^** | **Lower** | **Upper** | ***P*-value** | **FDR^b^** |
| rs10079641 | *MSH3* | 0.86 | 0.53 | 1.39 | 0.538 | 0.966 | 1.04 | 0.75 | 1.43 | 0.816 | 0.997 |
| rs1042522 | *TP53* | 0.79 | 0.55 | 1.13 | 0.190 | 0.875 | 0.72 | 0.49 | 1.05 | 0.092 | 0.588 |
| rs1047768 | *ERCC5* | 0.89 | 0.67 | 1.20 | 0.449 | 0.966 | 0.95 | 0.78 | 1.15 | 0.579 | 0.914 |
| rs1047840 | *EXO1* | 1.06 | 0.79 | 1.42 | 0.695 | 0.983 | 0.84 | 0.69 | 1.03 | 0.093 | 0.588 |
| rs1048771 | *RAD54L* | 1.16 | 0.79 | 1.71 | 0.447 | 0.966 | 0.79 | 0.57 | 1.10 | 0.155 | 0.685 |
| rs1051677 | *XRCC5* | 0.83 | 0.48 | 1.43 | 0.599 | 0.966 | 1.25 | 0.9 | 1.75 | 0.183 | 0.710 |
| rs1051685 | *XRCC5* | 1.03 | 0.63 | 1.69 | 0.900 | 0.983 | 0.98 | 0.72 | 1.35 | 0.910 | 0.997 |
| rs1052133 | *OGG1* | 0.96 | 0.68 | 1.36 | 0.817 | 0.983 | 0.93 | 0.73 | 1.17 | 0.529 | 0.914 |
| rs1059262 | *ALKBH2* | 1.06 | 0.74 | 1.52 | 0.757 | 0.983 | 0.85 | 0.66 | 1.09 | 0.189 | 0.710 |
| rs1060915 | *BRCA1* | 0.89 | 0.66 | 1.21 | 0.453 | 0.966 | 1.21 | 0.98 | 1.50 | 0.072 | 0.556 |
| rs11226 | *RAD52* | 1.29 | 0.95 | 1.76 | 0.107 | 0.875 | 1.18 | 0.97 | 1.44 | 0.099 | 0.588 |
| rs1130409 | *APEX1* | 1 | 0.76 | 1.31 | 0.999 | 0.999 | 0.86 | 0.71 | 1.04 | 0.125 | 0.612 |
| rs1136410 | *PARP1* | 1.33 | 1.02 | 1.77 | **0.044** | 0.642 | 1.05 | 0.79 | 1.40 | 0.730 | 0.950 |
| rs13180316 | *XRCC4* | 1.12 | 0.82 | 1.53 | 0.476 | 0.966 | 0.95 | 0.76 | 1.18 | 0.615 | 0.914 |
| rs13181 | *ERCC2* | 1.21 | 0.9 | 1.62 | 0.216 | 0.875 | 1.05 | 0.86 | 1.28 | 0.641 | 0.917 |
| rs1346044 | *WRN* | 0.97 | 0.67 | 1.39 | 0.854 | 0.983 | 0.84 | 0.66 | 1.05 | 0.130 | 0.612 |
| rs144848 | *BRCA2* | 1.08 | 0.8 | 1.47 | 0.608 | 0.966 | 1.10 | 0.88 | 1.38 | 0.395 | 0.832 |
| rs1478485 | *XRCC4* | 1.03 | 0.77 | 1.37 | 0.850 | 0.983 | 1.12 | 0.92 | 1.37 | 0.267 | 0.774 |
| rs1540354 | *MLH1* | 1.07 | 0.68 | 1.69 | 0.761 | 0.983 | 0.85 | 0.64 | 1.13 | 0.266 | 0.774 |
| rs1614984 | *TP53* | 1.12 | 0.86 | 1.45 | 0.407 | 0.944 | 1.03 | 0.83 | 1.26 | 0.802 | 0.997 |
| rs1618536 | *ERCC2* | 1.18 | 0.89 | 1.57 | 0.258 | 0.875 | 0.95 | 0.78 | 1.15 | 0.595 | 0.914 |
| rs1650697 | *MSH3* | 1.28 | 0.91 | 1.81 | 0.159 | 0.875 | 0.79 | 0.63 | 0.99 | **0.039** | 0.503 |
| rs174538 | *FEN1* | 0.91 | 0.66 | 1.26 | 0.575 | 0.966 | 1.04 | 0.84 | 1.28 | 0.744 | 0.957 |
| rs175080 | *MLH3* | 1.18 | 0.88 | 1.57 | 0.279 | 0.875 | 0.97 | 0.80 | 1.17 | 0.729 | 0.950 |
| rs1760944 | *APEX1* | 0.99 | 0.72 | 1.37 | 0.969 | 0.983 | 1.02 | 0.83 | 1.26 | 0.855 | 0.997 |
| rs17655 | *ERCC5* | 0.81 | 0.57 | 1.14 | 0.222 | 0.875 | 0.83 | 0.67 | 1.03 | 0.085 | 0.576 |
| rs176641 | *POLG* | 1.06 | 0.78 | 1.43 | 0.730 | 0.983 | 1.23 | 1.01 | 1.51 | **0.040** | 0.503 |
| rs1776148 | *EXO1* | 1.03 | 0.76 | 1.40 | 0.831 | 0.983 | 0.96 | 0.79 | 1.17 | 0.694 | 0.925 |
| rs1799793 | *ERCC2* | 1.35 | 1.01 | 1.82 | **0.047** | 0.642 | 0.98 | 0.81 | 1.22 | 0.973 | 0.997 |
| rs1799794 | *XRCC3* | 0.98 | 0.70 | 1.37 | 0.896 | 0.983 | 0.91 | 0.73 | 1.13 | 0.397 | 0.832 |
| rs1799796 | *XRCC3* | 0.92 | 0.66 | 1.29 | 0.639 | 0.983 | 0.88 | 0.70 | 1.12 | 0.298 | 0.785 |
| rs1799801 | *ERCC4* | 0.87 | 0.64 | 1.19 | 0.385 | 0.944 | 0.91 | 0.74 | 1.12 | 0.392 | 0.832 |
| rs1799955 | *BRCA2* | 0.91 | 0.64 | 1.29 | 0.591 | 0.966 | 1.01 | 0.79 | 1.28 | 0.972 | 0.997 |
| rs1799966 | *BRCA1* | 0.88 | 0.65 | 1.19 | 0.403 | 0.944 | 1.16 | 0.94 | 1.44 | 0.159 | 0.685 |
| rs1799977 | *MLH1* | 0.91 | 0.68 | 1.22 | 0.537 | 0.966 | 1.01 | 0.82 | 1.25 | 0.901 | 0.997 |
| rs1800067 | *ERCC4* | 0.99 | 0.63 | 1.56 | 0.974 | 0.983 | 0.92 | 0.68 | 1.25 | 0.588 | 0.914 |
| rs1800389 | *WRN* | 0.87 | 0.64 | 1.18 | 0.366 | 0.944 | 0.89 | 0.72 | 1.11 | 0.312 | 0.801 |
| rs1800734 | *MLH1* | 1.01 | 0.73 | 1.41 | 0.972 | 0.983 | 1.02 | 0.82 | 1.27 | 0.863 | 0.997 |
| rs1800935 | *MSH6* | 1.01 | 0.73 | 1.40 | 0.963 | 0.983 | 1.19 | 0.95 | 1.48 | 0.122 | 0.612 |
| rs1800975 | *XPA* | 0.90 | 0.66 | 1.21 | 0.473 | 0.966 | 0.94 | 0.77 | 1.16 | 0.575 | 0.914 |
| rs1801406 | *BRCA2* | 0.88 | 0.64 | 1.21 | 0.420 | 0.944 | 0.91 | 0.73 | 1.14 | 0.408 | 0.832 |
| rs1801516 | *ATM* | 1.13 | 0.77 | 1.66 | 0.537 | 0.966 | 1.12 | 0.84 | 1.5 | 0.427 | 0.854 |
| rs1802904 | *ATR* | 1.20 | 0.78 | 1.85 | 0.399 | 0.944 | 1.1 | 0.83 | 1.47 | 0.497 | 0.886 |
| rs1805386 | *LIG4* | 1.03 | 0.69 | 1.53 | 0.893 | 0.983 | 0.95 | 0.73 | 1.23 | 0.673 | 0.925 |
| rs1805388 | *LIG4* | 1.06 | 0.68 | 1.64 | 0.800 | 0.983 | 1.14 | 0.86 | 1.53 | 0.365 | 0.832 |
| rs1805794 | *NBS1* | 1.18 | 0.87 | 1.61 | 0.285 | 0.875 | 0.99 | 0.81 | 1.22 | 0.934 | 0.997 |
| rs1981928 | *MSH2* | 0.93 | 0.66 | 1.32 | 0.698 | 0.983 | 1 | 0.81 | 1.25 | 0.980 | 0.997 |
| rs2020911 | *MSH6* | 0.78 | 0.58 | 1.06 | 0.108 | 0.875 | 1.08 | 0.88 | 1.31 | 0.478 | 0.886 |
| rs2040639 | *XRCC2* | 0.91 | 0.68 | 1.22 | 0.539 | 0.966 | 0.88 | 0.73 | 1.07 | 0.191 | 0.710 |
| rs2048718 | *BRIP1* | 0.84 | 0.64 | 1.12 | 0.234 | 0.875 | 1.01 | 0.81 | 1.23 | 0.991 | 0.997 |
| rs20580 | *LIG1* | 0.82 | 0.62 | 1.09 | 0.168 | 0.875 | 1.22 | 1.01 | 1.47 | **0.042** | 0.503 |
| rs2074522 | *LIG3* | 1.27 | 0.88 | 1.84 | 0.197 | 0.875 | 1.13 | 0.8 | 1.59 | 0.500 | 0.886 |
| rs2075685 | *XRCC4* | 1.14 | 0.85 | 1.53 | 0.381 | 0.944 | 1.02 | 0.83 | 1.24 | 0.870 | 0.997 |
| rs207906 | *XRCC5* | 0.89 | 0.57 | 1.39 | 0.608 | 0.966 | 0.99 | 0.74 | 1.31 | 0.927 | 0.997 |
| rs2228000 | *XPC* | 0.78 | 0.57 | 1.08 | 0.133 | 0.875 | 0.83 | 0.68 | 1.02 | 0.077 | 0.556 |
| rs2228001 | *XPC* | 0.84 | 0.62 | 1.13 | 0.252 | 0.875 | 1.06 | 0.87 | 1.3 | 0.559 | 0.914 |
| rs2228006 | *PMS2* | 1.32 | 0.87 | 2.01 | 0.184 | 0.875 | 0.99 | 0.77 | 1.31 | 0.997 | 0.997 |
| rs2238463 | *ERCC4* | 0.85 | 0.63 | 1.15 | 0.301 | 0.878 | 0.81 | 0.66 | 0.99 | **0.042** | 0.503 |
| rs2252775 | *RAD50* | 0.85 | 0.58 | 1.23 | 0.384 | 0.944 | 1.12 | 0.87 | 1.43 | 0.378 | 0.832 |
| rs2272615 | *POLB* | 1.13 | 0.72 | 1.77 | 0.592 | 0.966 | 0.9 | 0.67 | 1.2 | 0.475 | 0.886 |
| rs2286940 | *MLH1* | 0.98 | 0.75 | 1.29 | 0.889 | 0.983 | 1.04 | 0.86 | 1.27 | 0.686 | 0.925 |
| rs2303428 | *MSH2* | 1.01 | 0.63 | 1.63 | 0.960 | 0.983 | 1.22 | 0.89 | 1.66 | 0.216 | 0.728 |
| rs2308321 | *MGMT* | 0.89 | 0.52 | 1.52 | 0.834 | 0.983 | 0.92 | 0.67 | 1.28 | 0.625 | 0.914 |
| rs2345060 | *PMS2* | 0.91 | 0.64 | 1.28 | 0.571 | 0.966 | 0.82 | 0.65 | 1.02 | 0.077 | 0.556 |
| rs2348244 | *MSH6* | 0.66 | 0.43 | 0.99 | **0.048** | 0.642 | 0.86 | 0.64 | 1.17 | 0.350 | 0.832 |
| rs238406 | *ERCC2* | 1.06 | 0.81 | 1.39 | 0.681 | 0.983 | 1.06 | 0.88 | 1.27 | 0.569 | 0.914 |
| rs2434470 | *ALKBH3* | 0.81 | 0.57 | 1.17 | 0.259 | 0.875 | 0.88 | 0.7 | 1.11 | 0.272 | 0.774 |
| rs2440 | *XRCC5* | 1.10 | 0.82 | 1.46 | 0.525 | 0.966 | 10.1 | 0.82 | 1.22 | 0.996 | 0.997 |
| rs25487 | *XRCC1* | 1.03 | 0.77 | 1.37 | 0.839 | 0.983 | 0.83 | 0.68 | 1.02 | 0.051 | 0.504 |
| rs26279 | *MSH3* | 1.18 | 0.88 | 1.59 | 0.269 | 0.875 | 0.95 | 0.78 | 1.16 | 0.623 | 0.914 |
| rs26779 | *MSH3* | 0.97 | 0.72 | 1.31 | 0.864 | 0.983 | 0.81 | 0.66 | 0.99 | **0.038** | 0.503 |
| rs293794 | *OGG1* | 0.85 | 0.57 | 1.26 | 0.420 | 0.944 | 1.10 | 0.85 | 1.42 | 0.489 | 0.886 |
| rs3136038 | *ERCC4* | 0.85 | 0.62 | 1.15 | 0.292 | 0.875 | 0.82 | 0.66 | 10.2 | 0.051 | 0.504 |
| rs3136228 | *MSH6* | 0.99 | 0.73 | 1.33 | 0.939 | 0.983 | 1.14 | 0.93 | 1.40 | 0.204 | 0.712 |
| rs3212948 | *ERCC1* | 1.15 | 0.86 | 1.55 | 0.343 | 0.944 | 0.95 | 0.78 | 1.15 | 0.583 | 0.914 |
| rs3212961 | *ERCC1* | 1.27 | 0.81 | 1.99 | 0.152 | 0.875 | 0.99 | 0.74 | 1.33 | 0.971 | 0.997 |
| rs3212986 | *ERCC1* | 1.22 | 0.87 | 1.73 | 0.248 | 0.875 | 0.91 | 0.73 | 1.14 | 0.401 | 0.832 |
| rs3213245 | *XRCC1* | 1.01 | 0.75 | 1.36 | 0.954 | 0.983 | 1.11 | 0.92 | 1.34 | 0.280 | 0.774 |
| rs3218536 | *XRCC2* | 1.01 | 0.61 | 1.65 | 0.847 | 0.983 | 1.08 | 0.78 | 1.49 | 0.645 | 0.917 |
| rs3219489 | *MUTYH* | 0.83 | 0.59 | 1.15 | 0.262 | 0.875 | 0.93 | 0.75 | 1.17 | 0.552 | 0.914 |
| rs3626 | *PCNA* | 0.94 | 0.62 | 1.42 | 0.773 | 0.983 | 0.93 | 0.69 | 1.25 | 0.627 | 0.914 |
| rs3730668 | *POLI* | 0.99 | 0.73 | 1.35 | 0.967 | 0.983 | 0.98 | 0.80 | 1.19 | 0.835 | 0.997 |
| rs3793784 | *ERCC6* | 0.95 | 0.72 | 1.25 | 0.719 | 0.983 | 0.9 | 0.73 | 1.09 | 0.274 | 0.774 |
| rs4150416 | *ERCC3* | 1.23 | 0.98 | 1.56 | 0.075 | 0.833 | 0.97 | 0.79 | 1.20 | 0.796 | 0.997 |
| rs4150441 | *ERCC3* | 1.23 | 0.91 | 1.67 | 0.177 | 0.875 | 1.02 | 0.84 | 1.24 | 0.865 | 0.997 |
| rs4150474 | *ERCC3* | 1.21 | 0.95 | 1.55 | 0.119 | 0.875 | 1.02 | 0.81 | 1.28 | 0.880 | 0.997 |
| rs4234259 | *MLH1* | 0.94 | 0.72 | 1.24 | 0.676 | 0.983 | 1.04 | 0.86 | 1.27 | 0.686 | 0.925 |
| rs4253160 | *ERCC6* | 0.87 | 0.66 | 1.15 | 0.319 | 0.908 | 0.93 | 0.77 | 1.13 | 0.481 | 0.886 |
| rs4968451 | *BRIP1* | 0.90 | 0.64 | 1.28 | 0.569 | 0.966 | 0.89 | 0.67 | 1.17 | 0.396 | 0.832 |
| rs4986764 | *BRIP1* | 0.79 | 0.59 | 1.06 | 0.112 | 0.875 | 0.88 | 0.72 | 1.07 | 0.199 | 0.712 |
| rs4987876 | *ATM* | 1.24 | 0.78 | 1.98 | 0.368 | 0.944 | 0.77 | 0.55 | 1.07 | 0.119 | 0.612 |
| rs569143 | *MRE11A* | 1.04 | 0.78 | 1.38 | 0.781 | 0.983 | 1.23 | 1.01 | 1.50 | **0.036** | 0.503 |
| rs5744934 | *POLE* | 1.14 | 0.79 | 1.63 | 0.485 | 0.966 | 1.01 | 0.78 | 1.30 | 0.965 | 0.997 |
| rs601341 | *MRE11A* | 1.06 | 0.79 | 1.42 | 0.714 | 0.983 | 1.10 | 0.86 | 1.41 | 0.462 | 0.886 |
| rs6413436 | *RAD52* | 1.19 | 0.87 | 1.62 | 0.272 | 0.875 | 1.13 | 0.92 | 1.37 | 0.240 | 0.773 |
| rs664143 | *ATM* | 0.80 | 0.60 | 1.08 | 0.144 | 0.875 | 0.90 | 0.74 | 1.11 | 0.325 | 0.816 |
| rs7182283 | *NEIL1* | 0.94 | 0.70 | 1.25 | 0.653 | 0.983 | 0.92 | 0.76 | 1.11 | 0.381 | 0.832 |
| rs735943 | *EXO1* | 0.98 | 0.75 | 1.32 | 0.974 | 0.983 | 0.89 | 0.74 | 1.08 | 0.243 | 0.773 |
| rs7797466 | *PMS2* | 1.25 | 0.84 | 1.85 | 0.272 | 0.875 | 1.02 | 0.79 | 1.33 | 0.858 | 0.997 |
| rs799917 | *BRCA1* | 0.84 | 0.62 | 1.14 | 0.259 | 0.875 | 1.19 | 0.96 | 1.47 | 0.104 | 0.588 |
| rs8305 | *POLI* | 0.98 | 0.71 | 1.36 | 0.907 | 0.983 | 1.23 | 0.99 | 1.53 | 0.057 | 0.517 |
| rs861528 | *XRCC3* | 1.39 | 0.97 | 1.98 | 0.069 | 0.833 | 1.13 | 0.9 | 1.43 | 0.298 | 0.785 |
| rs861531 | *XRCC3* | 1.08 | 0.81 | 1.46 | 0.596 | 0.966 | 1.08 | 0.88 | 1.32 | 0.461 | 0.886 |
| rs861539 | *XRCC3* | 1.03 | 0.77 | 1.38 | 0.837 | 0.983 | 1.02 | 0.83 | 1.24 | 0.869 | 0.997 |
| rs9350 | *EXO1* | 1.10 | 0.74 | 1.65 | 0.640 | 0.983 | 0.83 | 0.64 | 1.08 | 0.165 | 0.685 |
| rs963248 | *XRCC4* | 0.97 | 0.65 | 1.43 | 0.866 | 0.983 | 1.06 | 0.82 | 1.37 | 0.675 | 0.925 |
| rs9876116 | *MLH1* | 0.93 | 0.71 | 1.21 | 0.576 | 0.966 | 1.01 | 0.83 | 1.23 | 0.919 | 0.997 |
| rs9894946 | *TP53* | 0.78 | 0.51 | 1.20 | 0.158 | 0.875 | 0.77 | 0.62 | 0.96 | **0.017** | 0.503 |

OR, odds ratio; CI, confidence interval.

^a^ORs adjusted by age, *Helicobacter pylori* infection, smoking status, and family history of gastric cancer.

^b^QFDR-values obtained after applying the False Discovery Rate (FDR) test.

*P*-values <0.05 are highlighted in bold.
