# Supplementary material for: Relevance of DNA repair gene polymorphisms to gastric cancer risk and phenotype
Source: Oncotarget. 2017 Mar 16;8(22):35848–62. doi: 10.18632/oncotarget.16261 (PMC5482622; doi:10.18632/oncotarget.16261)
Supplement: Supplementary file 9 [file oncotarget-08-35848-s009.doc]

**Supplementary Table 9: Association of DNA repair gene polymorphisms with gastric cancer risk. Stratified analysis by smoking status (never smokers / current smokers)**.

| **Never smokers** | | | | | | | **Current smokers** | | | | |
| --- | --- | --- | --- | --- | --- | --- | --- | --- | --- | --- | --- |
|  |  | **Log-additive genetic model** | | | | | **Log-additive genetic model** | | | | |
|  |  |  | **95% CI** | |  |  |  | **95% CI** | |  |  |
| **db SNP ID** | **Gen** | **OR^a^** | **Lower** | **Upper** | ***P-*value** | **FDR^b^** | **OR^a^** | **Lower** | **Upper** | ***P*-value** | **FDR^b^** |
| rs10079641 | *MSH3* | 0.90 | 0.62 | 1.33 | 0.611 | 0.957 | 1.50 | 0.80 | 2.83 | 0.195 | 0.994 |
| rs1042522 | *TP53* | 0.81 | 0.62 | 1.06 | 0.124 | 0.810 | 0.60 | 0.38 | 0.96 | **0.031** | 0.943 |
| rs1047768 | *ERCC5* | 0.88 | 0.71 | 1.10 | 0.276 | 0.927 | 1.02 | 0.71 | 1.48 | 0.900 | 0.994 |
| rs1047840 | *EXO1* | 0.81 | 0.64 | 1.02 | 0.076 | 0.810 | 1.11 | 0.75 | 1.66 | 0.600 | 0.994 |
| rs1048771 | *RAD54L* | 1.24 | 0.87 | 1.76 | 0.232 | 0.927 | 0.79 | 0.40 | 1.57 | 0.568 | 0.994 |
| rs1051677 | *XRCC5* | 1.39 | 0.91 | 2.12 | 0.173 | 0.810 | 0.76 | 0.40 | 1.45 | 0.477 | 0.994 |
| rs1051685 | *XRCC5* | 1.01 | 0.69 | 1.45 | 0.989 | 0.990 | 0.90 | 0.46 | 1.73 | 0.743 | 0.994 |
| rs1052133 | *OGG1* | 0.86 | 0.65 | 1.13 | 0.274 | 0.927 | 1.09 | 0.67 | 1.78 | 0.720 | 0.994 |
| rs1059262 | *ALKBH2* | 0.90 | 0.67 | 1.21 | 0.492 | 0.949 | 1.10 | 0.68 | 1.78 | 0.689 | 0.994 |
| rs1060915 | *BRCA1* | 1.09 | 0.85 | 1.39 | 0.492 | 0.949 | 1.01 | 0.67 | 1.51 | 0.965 | 0.994 |
| rs11226 | *RAD52* | 1.25 | 0.98 | 1.58 | 0.072 | 0.810 | 1.31 | 0.87 | 1.96 | 0.188 | 0.994 |
| rs1130409 | *APEX1* | 0.93 | 0.75 | 1.16 | 0.518 | 0.949 | 0.80 | 0.54 | 1.19 | 0.267 | 0.994 |
| rs1136410 | *PARP1* | 1.36 | 0.97 | 1.91 | 0.076 | 0.810 | 0.91 | 0.52 | 1.58 | 0.727 | 0.994 |
| rs13180316 | *XRCC4* | 0.97 | 0.76 | 1.23 | 0.782 | 0.987 | 0.91 | 0.58 | 1.44 | 0.702 | 0.994 |
| rs13181 | *ERCC2* | 1.15 | 0.91 | 1.46 | 0.234 | 0.927 | 1.03 | 0.69 | 1.55 | 0.884 | 0.994 |
| rs1346044 | *WRN* | 0.96 | 0.72 | 1.27 | 0.765 | 0.987 | 1.03 | 0.65 | 1.61 | 0.907 | 0.994 |
| rs144848 | *BRCA2* | 1.06 | 0.82 | 1.37 | 0.641 | 0.976 | 1.10 | 0.72 | 1.69 | 0.653 | 0.994 |
| rs1478485 | *XRCC4* | 1.15 | 0.91 | 1.44 | 0.243 | 0.927 | 0.95 | 0.63 | 1.41 | 0.785 | 0.994 |
| rs1540354 | *MLH1* | 0.85 | 0.60 | 1.21 | 0.362 | 0.949 | 1.15 | 0.65 | 2.04 | 0.631 | 0.994 |
| rs1614984 | *TP53* | 1.20 | 0.94 | 1.53 | 0.148 | 0.810 | 0.79 | 0.52 | 1.21 | 0.283 | 0.994 |
| rs1618536 | *ERCC2* | 1.13 | 0.90 | 1.42 | 0.280 | 0.927 | 0.83 | 0.55 | 1.24 | 0.358 | 0.994 |
| rs1650697 | *MSH3* | 1 | 0.77 | 1.30 | 0.990 | 0.990 | 0.58 | 0.37 | 0.91 | **0.016** | 0.943 |
| rs174538 | *FEN1* | 0.99 | 0.77 | 1.28 | 0.963 | 0.987 | 0.98 | 0.64 | 1.49 | 0.918 | 0.994 |
| rs175080 | *MLH3* | 1.01 | 0.80 | 1.27 | 0.943 | 0.987 | 0.92 | 0.64 | 1.34 | 0.676 | 0.994 |
| rs1760944 | *APEX1* | 0.95 | 0.75 | 1.22 | 0.707 | 0.979 | 1.11 | 0.71 | 1.74 | 0.651 | 0.994 |
| rs17655 | *ERCC5* | 0.77 | 0.59 | 1.01 | 0.058 | 0.810 | 1.01 | 0.66 | 1.54 | 0.963 | 0.994 |
| rs176641 | *POLG* | 1.08 | 0.85 | 1.37 | 0.554 | 0.949 | 1.58 | 1.04 | 2.42 | 0.031 | 0.943 |
| rs1776148 | *EXO1* | 1.01 | 0.80 | 1.29 | 0.914 | 0.987 | 1.09 | 0.74 | 1.62 | 0.662 | 0.994 |
| rs1799793 | *ERCC2* | 1.14 | 0.90 | 1.46 | 0.270 | 0.927 | 1.16 | 0.77 | 1.77 | 0.476 | 0.994 |
| rs1799794 | *XRCC3* | 0.95 | 0.73 | 1.23 | 0.679 | 0.979 | 0.87 | 0.57 | 1.33 | 0.517 | 0.994 |
| rs1799796 | *XRCC3* | 1.01 | 0.78 | 1.32 | 0.926 | 0.987 | 0.89 | 0.55 | 1.44 | 0.629 | 0.994 |
| rs1799801 | *ERCC4* | 0.93 | 0.73 | 1.18 | 0.536 | 0.949 | 0.82 | 0.55 | 1.25 | 0.360 | 0.994 |
| rs1799955 | *BRCA2* | 0.99 | 0.75 | 1.30 | 0.918 | 0.987 | 1.05 | 0.64 | 1.72 | 0.839 | 0.994 |
| rs1799966 | *BRCA1* | 1.05 | 0.82 | 1.34 | 0.701 | 0.979 | 1.01 | 0.67 | 1.51 | 0.965 | 0.994 |
| rs1799977 | *MLH1* | 0.99 | 0.78 | 1.25 | 0.915 | 0.987 | 0.9 | 0.61 | 1.34 | 0.608 | 0.994 |
| rs1800067 | *ERCC4* | 0.95 | 0.67 | 1.33 | 0.756 | 0.987 | 1.02 | 0.54 | 1.92 | 0.957 | 0.994 |
| rs1800389 | *WRN* | 0.98 | 0.76 | 1.25 | 0.861 | 0.987 | 0.94 | 0.57 | 1.54 | 0.799 | 0.994 |
| rs1800734 | *MLH1* | 0.95 | 0.73 | 1.23 | 0.673 | 0.979 | 1.07 | 0.69 | 1.65 | 0.769 | 0.994 |
| rs1800935 | *MSH6* | 1.09 | 0.83 | 1.42 | 0.541 | 0.949 | 1.06 | 0.70 | 1.62 | 0.769 | 0.994 |
| rs1800975 | *XPA* | 0.91 | 0.71 | 1.15 | 0.425 | 0.949 | 0.82 | 0.54 | 1.24 | 0.338 | 0.994 |
| rs1801406 | *BRCA2* | 0.99 | 0.77 | 1.29 | 0.969 | 0.987 | 1.02 | 0.65 | 1.59 | 0.943 | 0.994 |
| rs1801516 | *ATM* | 0.90 | 0.65 | 1.25 | 0.536 | 0.949 | 1.47 | 0.83 | 2.62 | 0.293 | 0.994 |
| rs1802904 | *ATR* | 1.18 | 0.85 | 1.65 | 0.318 | 0.949 | 1.35 | 0.72 | 2.52 | 0.536 | 0.994 |
| rs1805386 | *LIG4* | 0.98 | 0.72 | 1.34 | 0.895 | 0.987 | 0.81 | 0.48 | 1.36 | 0.424 | 0.994 |
| rs1805388 | *LIG4* | 1.36 | 0.96 | 1.91 | 0.079 | 0.810 | 0.91 | 0.50 | 1.65 | 0.133 | 0.994 |
| rs1805794 | *NBS1* | 1.07 | 0.84 | 1.35 | 0.603 | 0.957 | 1.03 | 0.69 | 1.55 | 0.872 | 0.994 |
| rs1981928 | *MSH2* | 0.98 | 0.75 | 1.28 | 0.884 | 0.987 | 0.76 | 0.50 | 1.14 | 0.176 | 0.994 |
| rs2020911 | *MSH6* | 0.92 | 0.73 | 1.17 | 0.501 | 0.949 | 0.99 | 0.66 | 1.47 | 0.950 | 0.994 |
| rs2040639 | *XRCC2* | 0.92 | 0.74 | 1.16 | 0.491 | 0.949 | 1.01 | 0.67 | 1.49 | 0.995 | 0.995 |
| rs2048718 | *BRIP1* | 1.11 | 0.89 | 1.39 | 0.362 | 0.949 | 1.01 | 0.67 | 1.52 | 0.972 | 0.994 |
| rs20580 | *LIG1* | 1.08 | 0.87 | 1.35 | 0.493 | 0.949 | 1.28 | 0.88 | 1.87 | 0.194 | 0.994 |
| rs2074522 | *LIG3* | 1.34 | 0.91 | 1.97 | 0.136 | 0.810 | 1.59 | 0.81 | 3.12 | 0.325 | 0.994 |
| rs2075685 | *XRCC4* | 1.11 | 0.88 | 1.39 | 0.397 | 0.949 | 0.96 | 0.65 | 1.43 | 0.848 | 0.994 |
| rs207906 | *XRCC5* | 0.79 | 0.56 | 1.10 | 0.156 | 0.810 | 1.08 | 0.58 | 2.01 | 0.408 | 0.994 |
| rs2228000 | *XPC* | 0.81 | 0.63 | 1.05 | 0.105 | 0.810 | 0.83 | 0.56 | 1.21 | 0.324 | 0.994 |
| rs2228001 | *XPC* | 0.88 | 0.69 | 1.11 | 0.280 | 0.927 | 1.09 | 0.74 | 1.62 | 0.667 | 0.994 |
| rs2228006 | *PMS2* | 1.26 | 0.92 | 1.73 | 0.150 | 0.810 | 0.85 | 0.51 | 1.40 | 0.517 | 0.994 |
| rs2238463 | *ERCC4* | 0.91 | 0.72 | 1.15 | 0.412 | 0.949 | 0.67 | 0.45 | 1 | **0.048** | 0.943 |
| rs2252775 | *RAD50* | 0.92 | 0.69 | 1.24 | 0.595 | 0.957 | 1.16 | 0.7 | 1.93 | 0.557 | 0.994 |
| rs2272615 | *POLB* | 1.08 | 0.76 | 1.53 | 0.675 | 0.979 | 0.84 | 0.48 | 1.50 | 0.566 | 0.994 |
| rs2286940 | *MLH1* | 1.10 | 0.89 | 1.37 | 0.377 | 0.949 | 0.92 | 0.63 | 1.36 | 0.689 | 0.994 |
| rs2303428 | *MSH2* | 0.89 | 0.60 | 1.31 | 0.552 | 0.949 | 0.98 | 0.59 | 1.63 | 0.941 | 0.994 |
| rs2308321 | *MGMT* | 1.02 | 0.69 | 1.52 | 0.908 | 0.987 | 1.03 | 0.52 | 2.03 | 0.159 | 0.994 |
| rs2345060 | *PMS2* | 0.92 | 0.70 | 1.19 | 0.517 | 0.949 | 0.75 | 0.47 | 1.19 | 0.219 | 0.994 |
| rs2348244 | *MSH6* | 0.71 | 0.50 | 1.01 | 0.054 | 0.810 | 1.19 | 0.64 | 2.23 | 0.584 | 0.994 |
| rs238406 | *ERCC2* | 1.01 | 0.81 | 1.25 | 0.945 | 0.987 | 1.03 | 0.69 | 1.52 | 0.896 | 0.994 |
| rs2434470 | *ALKBH3* | 0.78 | 0.59 | 1.04 | 0.089 | 0.810 | 0.74 | 0.49 | 1.13 | 0.162 | 0.994 |
| rs2440 | *XRCC5* | 1.02 | 0.81 | 1.27 | 0.887 | 0.987 | 1.34 | 0.90 | 2 | 0.142 | 0.994 |
| rs25487 | *XRCC1* | 0.92 | 0.74 | 1.15 | 0.460 | 0.949 | 0.77 | 0.53 | 1.11 | 0.160 | 0.994 |
| rs26279 | *MSH3* | 1.09 | 0.86 | 1.37 | 0.486 | 0.949 | 0.95 | 0.64 | 1.43 | 0.820 | 0.994 |
| rs26779 | *MSH3* | 0.92 | 0.73 | 1.17 | 0.505 | 0.949 | 0.67 | 0.45 | 1.01 | 0.052 | 0.943 |
| rs293794 | *OGG1* | 1.03 | 0.75 | 1.40 | 0.873 | 0.987 | 0.80 | 0.47 | 1.36 | 0.411 | 0.994 |
| rs3136038 | *ERCC4* | 0.91 | 0.71 | 1.15 | 0.414 | 0.949 | 0.66 | 0.44 | 0.99 | **0.043** | 0.943 |
| rs3136228 | *MSH6* | 1.04 | 0.82 | 1.32 | 0.757 | 0.987 | 0.98 | 0.66 | 1.44 | 0.906 | 0.994 |
| rs3212948 | *ERCC1* | 1.05 | 0.83 | 1.31 | 0.703 | 0.979 | 1.10 | 0.73 | 1.65 | 0.665 | 0.994 |
| rs3212961 | *ERCC1* | 1.10 | 0.77 | 1.58 | 0.595 | 0.957 | 1.30 | 0.74 | 2.27 | 0.362 | 0.994 |
| rs3212986 | *ERCC1* | 1.09 | 0.83 | 1.44 | 0.517 | 0.949 | 0.93 | 0.58 | 1.50 | 0.777 | 0.994 |
| rs3213245 | *XRCC1* | 1.01 | 0.81 | 1.27 | 0.929 | 0.987 | 1.27 | 0.86 | 1.88 | 0.228 | 0.994 |
| rs3218536 | *XRCC2* | 1.06 | 0.71 | 1.57 | 0.407 | 0.949 | 0.81 | 0.44 | 1.47 | 0.097 | 0.994 |
| rs3219489 | *MUTYH* | 0.80 | 0.62 | 1.05 | 0.108 | 0.810 | 1.12 | 0.71 | 1.75 | 0.631 | 0.994 |
| rs3626 | *PCNA* | 0.98 | 0.69 | 1.39 | 0.909 | 0.987 | 0.8 | 0.43 | 1.49 | 0.697 | 0.994 |
| rs3730668 | *POLI* | 0.91 | 0.72 | 1.15 | 0.423 | 0.949 | 0.99 | 0.66 | 1.48 | 0.955 | 0.994 |
| rs3793784 | *ERCC6* | 0.93 | 0.75 | 1.16 | 0.538 | 0.949 | 0.71 | 0.48 | 1.06 | 0.096 | 0.994 |
| rs4150416 | *ERCC3* | 0.74 | 0.57 | 0.94 | **0.015** | 0.796 | 0.91 | 0.60 | 1.36 | 0.637 | 0.994 |
| rs4150441 | *ERCC3* | 1.14 | 0.90 | 1.44 | 0.283 | 0.927 | 1.02 | 0.69 | 1.52 | 0.906 | 0.994 |
| rs4150474 | *ERCC3* | 0.86 | 0.70 | 1.12 | 0.215 | 0.927 | 0.92 | 0.60 | 1.41 | 0.696 | 0.994 |
| rs4234259 | *MLH1* | 0.92 | 0.74 | 1.14 | 0.431 | 0.949 | 0.94 | 0.64 | 1.38 | 0.751 | 0.994 |
| rs4253160 | *ERCC6* | 0.89 | 0.71 | 1.11 | 0.313 | 0.949 | 0.72 | 0.48 | 1.06 | 0.096 | 0.994 |
| rs4968451 | *BRIP1* | 1.06 | 0.80 | 1.42 | 0.684 | 0.979 | 0.74 | 0.42 | 1.31 | 0.306 | 0.994 |
| rs4986764 | *BRIP1* | 0.84 | 0.67 | 1.05 | 0.132 | 0.810 | 0.96 | 0.64 | 1.44 | 0.833 | 0.994 |
| rs4987876 | *ATM* | 1.29 | 0.89 | 1.87 | 0.171 | 0.810 | 0.63 | 0.33 | 1.22 | 0.170 | 0.994 |
| rs569143 | *MRE11A* | 0.93 | 0.74 | 1.17 | 0.532 | 0.949 | 0.78 | 0.53 | 1.15 | 0.210 | 0.994 |
| rs5744934 | *POLE* | 1.04 | 0.77 | 1.40 | 0.798 | 0.987 | 1.18 | 0.70 | 2 | 0.093 | 0.994 |
| rs601341 | *MRE11A* | 0.93 | 0.74 | 1.18 | 0.566 | 0.955 | 0.81 | 0.55 | 1.20 | 0.297 | 0.994 |
| rs6413436 | *RAD52* | 1.19 | 0.94 | 1.50 | 0.160 | 0.810 | 1.05 | 0.69 | 1.59 | 0.813 | 0.994 |
| rs664143 | *ATM* | 0.82 | 0.65 | 1.04 | 0.108 | 0.810 | 0.98 | 0.67 | 1.44 | 0.913 | 0.994 |
| rs7182283 | *NEIL1* | 0.96 | 0.76 | 1.22 | 0.757 | 0.987 | 0.81 | 0.56 | 1.17 | 0.266 | 0.994 |
| rs735943 | *EXO1* | 0.97 | 0.78 | 1.22 | 0.823 | 0.987 | 1 | 0.70 | 1.44 | 0.985 | 0.994 |
| rs7797466 | *PMS2* | 0.93 | 0.68 | 1.27 | 0.629 | 0.970 | 0.99 | 0.63 | 1.57 | 0.977 | 0.994 |
| rs799917 | *BRCA1* | 1.04 | 0.82 | 1.33 | 0.724 | 0.987 | 1.10 | 0.73 | 1.64 | 0.650 | 0.994 |
| rs8305 | *POLI* | 1.17 | 0.90 | 1.51 | 0.234 | 0.927 | 0.98 | 0.65 | 1.48 | 0.924 | 0.994 |
| rs861528 | *XRCC3* | 1.24 | 0.94 | 1.63 | 0.131 | 0.810 | 1.14 | 0.72 | 1.81 | 0.564 | 0.994 |
| rs861531 | *XRCC3* | 1.01 | 0.79 | 1.28 | 0.965 | 0.987 | 1.24 | 0.84 | 1.84 | 0.275 | 0.994 |
| rs861539 | *XRCC3* | 0.97 | 0.77 | 1.23 | 0.797 | 0.987 | 1.17 | 0.79 | 1.73 | 0.437 | 0.994 |
| rs9350 | *EXO1* | 0.98 | 0.71 | 1.34 | 0.883 | 0.987 | 0.81 | 0.49 | 1.32 | 0.399 | 0.994 |
| rs963248 | *XRCC4* | 0.97 | 0.71 | 1.33 | 0.869 | 0.987 | 1.19 | 0.73 | 1.93 | 0.489 | 0.994 |
| rs9876116 | *MLH1* | 1.06 | 0.85 | 1.31 | 0.597 | 0.957 | 0.87 | 0.59 | 1.28 | 0.467 | 0.994 |
| rs9894946 | *TP53* | 0.69 | 0.49 | 0.97 | **0.033** | 0.810 | 0.88 | 0.53 | 1.47 | 0.636 | 0.994 |

OR, odds ratio; CI, confidence interval.

^a^ORs adjusted by gender, age, *Helicobacter pylori* infection, and family history of gastric cancer.

^b^QFDR-values obtained after applying the False Discovery Rate (FDR) test.

*P*-values <0.05 are highlighted in bold.
