# Supplementary material for: Relevance of DNA repair gene polymorphisms to gastric cancer risk and phenotype
Source: Oncotarget. 2017 Mar 16;8(22):35848–62. doi: 10.18632/oncotarget.16261 (PMC5482622; doi:10.18632/oncotarget.16261)
Supplement: Supplementary file 10 [file oncotarget-08-35848-s010.doc]

**Supplementary Table 10: Association of DNA repair gene polymorphisms with gastric cancer risk. Stratified analysis by family history of gastric cancer**.

| **Family history of GC (negative)** | | | | | | | **Family history of GC (positive)** | | | | |
| --- | --- | --- | --- | --- | --- | --- | --- | --- | --- | --- | --- |
|  |  | **Log-additive genetic model** | | | | | **Log-additive genetic model** | | | | |
|  |  |  | **95% CI** | |  |  |  | **95% CI** | |  |  |
| **db SNP ID** | **Gen** | **OR^a^** | **Lower** | **Upper** | ***P-*value** | **FDR^b^** | **OR^a^** | **Lower** | **Upper** | ***P*-value** | **FDR^b^** |
| rs10079641 | *MSH3* | 1.08 | 0.80 | 1.46 | 0.609 | 0.936 | 0.70 | 0.29 | 1.70 | 0.501 | 0.840 |
| rs1042522 | *TP53* | 0.82 | 0.66 | 1.01 | 0.056 | 0.473 | 0.58 | 0.31 | 1.07 | 0.083 | 0.709 |
| rs1047768 | *ERCC5* | 0.96 | 0.81 | 1.15 | 0.677 | 0.936 | 1.03 | 0.58 | 1.83 | 0.916 | 1 |
| rs1047840 | *EXO1* | 0.88 | 0.73 | 1.07 | 0.194 | 0.626 | 1.03 | 0.57 | 1.87 | 0.926 | 1 |
| rs1048771 | *RAD54L* | 1.06 | 0.79 | 1.41 | 0.714 | 0.952 | 1.19 | 0.41 | 3.46 | 1 | 1 |
| rs1051677 | *XRCC5* | 1.23 | 0.90 | 1.69 | 0.195 | 0.626 | 0.73 | 0.26 | 2.04 | 0.585 | 0.867 |
| rs1051685 | *XRCC5* | 0.97 | 0.72 | 1.31 | 0.832 | 0.992 | 1.54 | 0.50 | 4.76 | 0.071 | 0.709 |
| rs1052133 | *OGG1* | 1.06 | 0.85 | 1.32 | 0.617 | 0.936 | 1.08 | 0.82 | 1.41 | 0.592 | 0.867 |
| rs1059262 | *ALKBH2* | 0.92 | 0.73 | 1.16 | 0.494 | 0.889 | 0.81 | 0.39 | 1.66 | 0.564 | 0.867 |
| rs1060915 | *BRCA1* | 1.05 | 0.86 | 1.28 | 0.616 | 0.936 | 1.23 | 0.66 | 2.28 | 0.514 | 0.840 |
| rs11226 | *RAD52* | 1.13 | 0.87 | 1.46 | 0.360 | 0.806 | 0.79 | 0.45 | 1.39 | 0.408 | 0.819 |
| rs1130409 | *APEX1* | 0.85 | 0.71 | 1.01 | 0.070 | 0.473 | 1.41 | 0.81 | 2.45 | 0.217 | 0.757 |
| rs1136410 | *PARP1* | 1.19 | 0.90 | 1.57 | 0.220 | 0.626 | 1.52 | 0.67 | 3.46 | 0.672 | 0.931 |
| rs13180316 | *XRCC4* | 1.01 | 0.82 | 1.23 | 0.987 | 0.992 | 1.21 | 0.62 | 2.37 | 0.570 | 0.867 |
| rs13181 | *ERCC2* | 1.07 | 0.89 | 1.28 | 0.483 | 0.888 | 1.24 | 0.66 | 2.32 | 0.508 | 0.840 |
| rs1346044 | *WRN* | 0.91 | 0.73 | 1.13 | 0.395 | 0.806 | 1.06 | 0.49 | 2.28 | 0.885 | 1 |
| rs144848 | *BRCA2* | 1.15 | 0.94 | 1.41 | 0.167 | 0.626 | 1.18 | 0.56 | 2.47 | 0.859 | 1 |
| rs1478485 | *XRCC4* | 1.01 | 0.84 | 1.21 | 0.931 | 0.992 | 1.10 | 0.61 | 1.97 | 0.746 | 0.956 |
| rs1540354 | *MLH1* | 0.96 | 0.73 | 1.26 | 0.746 | 0.971 | 0.59 | 0.24 | 1.45 | 0.252 | 0.757 |
| rs1614984 | *TP53* | 1.11 | 0.92 | 1.35 | 0.267 | 0.642 | 1.47 | 0.81 | 2.67 | 0.194 | 0.757 |
| rs1618536 | *ERCC2* | 0.91 | 0.76 | 1.09 | 0.287 | 0.659 | 1.34 | 0.75 | 2.39 | 0.326 | 0.783 |
| rs1650697 | *MSH3* | 0.87 | 0.71 | 1.08 | 0.203 | 0.626 | 1.18 | 0.60 | 2.35 | 0.625 | 0.913 |
| rs174538 | *FEN1* | 1.01 | 0.83 | 1.23 | 0.924 | 0.992 | 0.89 | 0.47 | 1.71 | 0.731 | 0.956 |
| rs175080 | *MLH3* | 0.99 | 0.83 | 1.18 | 0.907 | 0.992 | 1.59 | 0.88 | 2.87 | 0.114 | 0.709 |
| rs1760944 | *APEX1* | 0.97 | 0.79 | 1.19 | 0.769 | 0.980 | 1.53 | 0.81 | 2.92 | 0.183 | 0.757 |
| rs17655 | *ERCC5* | 0.89 | 0.73 | 1.09 | 0.273 | 0.642 | 0.53 | 0.25 | 1.08 | 0.081 | 0.709 |
| rs176641 | *POLG* | 1.06 | 0.83 | 1.35 | 0.634 | 0.936 | 0.97 | 0.53 | 1.79 | 0.933 | 1 |
| rs1776148 | *EXO1* | 1.06 | 0.87 | 1.28 | 0.568 | 0.936 | 1.50 | 0.82 | 2.72 | 0.177 | 0.757 |
| rs1799793 | *ERCC2* | 1.09 | 0.90 | 1.32 | 0.386 | 0.806 | 0.74 | 0.42 | 1.32 | 0.309 | 0.783 |
| rs1799794 | *XRCC3* | 0.88 | 0.71 | 1.08 | 0.219 | 0.626 | 2.07 | 0.90 | 4.74 | 0.265 | 0.757 |
| rs1799796 | *XRCC3* | 0.96 | 0.77 | 1.20 | 0.737 | 0.971 | 0.53 | 0.26 | 1.06 | 0.072 | 0.709 |
| rs1799801 | *ERCC4* | 0.89 | 0.74 | 1.08 | 0.255 | 0.642 | 0.79 | 0.42 | 1.48 | 0.465 | 0.840 |
| rs1799955 | *BRCA2* | 1.05 | 0.84 | 1.31 | 0.693 | 0.936 | 0.47 | 0.21 | 1.02 | 0.062 | 0.709 |
| rs1799966 | *BRCA1* | 1.02 | 0.84 | 1.24 | 0.860 | 0.992 | 1.22 | 0.66 | 2.26 | 0.528 | 0.851 |
| rs1799977 | *MLH1* | 0.96 | 0.79 | 1.16 | 0.661 | 0.936 | 1.34 | 0.66 | 2.72 | 0.427 | 0.824 |
| rs1800067 | *ERCC4* | 0.81 | 0.61 | 1.08 | 0.150 | 0.624 | 1.46 | 0.58 | 3.68 | 0.736 | 0.956 |
| rs1800389 | *WRN* | 0.89 | 0.73 | 1.09 | 0.257 | 0.642 | 0.66 | 0.35 | 1.24 | 0.197 | 0.757 |
| rs1800734 | *MLH1* | 1.01 | 0.82 | 1.24 | 0.933 | 0.992 | 0.92 | 0.47 | 1.81 | 0.816 | 1 |
| rs1800935 | *MSH6* | 1.14 | 0.93 | 1.41 | 0.212 | 0.626 | 0.66 | 0.36 | 1.20 | 0.175 | 0.757 |
| rs1800975 | *XPA* | 0.89 | 0.74 | 1.08 | 0.244 | 0.642 | 0.73 | 0.39 | 1.37 | 0.333 | 0.783 |
| rs1801406 | *BRCA2* | 0.87 | 0.70 | 1.06 | 0.169 | 0.626 | 0.92 | 0.44 | 1.96 | 0.834 | 1 |
| rs1801516 | *ATM* | 1.21 | 0.94 | 1.57 | 0.144 | 0.624 | 0.84 | 0.37 | 1.88 | 0.677 | 0.931 |
| rs1802904 | *ATR* | 1.26 | 0.96 | 1.64 | 0.096 | 0.546 | 0.95 | 0.44 | 2.03 | 0.478 | 0.840 |
| rs1805386 | *LIG4* | 1.03 | 0.81 | 1.32 | 0.804 | 0.992 | 0.97 | 0.43 | 2.17 | 0.326 | 0.783 |
| rs1805388 | *LIG4* | 1.06 | 0.81 | 1.39 | 0.693 | 0.936 | 3.24 | 1.06 | 9.87 | 0.100 | 0.709 |
| rs1805794 | *NBS1* | 1.05 | 0.87 | 1.27 | 0.633 | 0.936 | 1.25 | 0.66 | 2.37 | 0.491 | 0.840 |
| rs1981928 | *MSH2* | 1.02 | 0.81 | 1.22 | 0.967 | 0.992 | 0.99 | 0.51 | 1.96 | 0.986 | 1 |
| rs2020911 | *MSH6* | 0.99 | 0.82 | 1.20 | 0.958 | 0.992 | 0.98 | 0.55 | 1.76 | 0.957 | 1 |
| rs2040639 | *XRCC2* | 0.93 | 0.77 | 1.11 | 0.395 | 0.806 | 0.97 | 0.55 | 1.71 | 0.918 | 1 |
| rs2048718 | *BRIP1* | 0.98 | 0.82 | 1.18 | 0.850 | 0.992 | 1.38 | 0.70 | 2.72 | 0.344 | 0.791 |
| rs20580 | *LIG1* | 1.18 | 0.99 | 1.41 | 0.067 | 0.473 | 0.76 | 0.44 | 1.31 | 0.326 | 0.783 |
| rs2074522 | *LIG3* | 1.19 | 0.87 | 1.62 | 0.270 | 0.642 | 1.34 | 0.52 | 3.43 | 1 | 1 |
| rs2075685 | *XRCC4* | 0.94 | 0.78 | 1.13 | 0.517 | 0.916 | 1.33 | 0.72 | 2.44 | 0.358 | 0.805 |
| rs207906 | *XRCC5* | 0.84 | 0.64 | 1.10 | 0.209 | 0.626 | 1.20 | 0.49 | 2.93 | 0.686 | 0.938 |
| rs2228000 | *XPC* | 0.82 | 0.68 | 0.99 | **0.048** | 0.473 | 1.08 | 0.54 | 2.18 | 0.821 | 1 |
| rs2228001 | *XPC* | 1.05 | 0.87 | 1.26 | 0.620 | 0.936 | 0.74 | 0.42 | 1.32 | 0.307 | 0.783 |
| rs2228006 | *PMS2* | 1.02 | 0.79 | 1.32 | 0.875 | 0.992 | 0.93 | 0.45 | 1.94 | 0.244 | 0.757 |
| rs2238463 | *ERCC4* | 0.84 | 0.69 | 1.01 | 0.061 | 0.473 | 0.62 | 0.34 | 1.14 | 0.123 | 0.709 |
| rs2252775 | *RAD50* | 1.01 | 0.80 | 1.26 | 0.971 | 0.992 | 1.40 | 0.65 | 2.99 | 0.377 | 0.814 |
| rs2272615 | *POLB* | 0.92 | 0.70 | 1.20 | 0.535 | 0.931 | 0.68 | 0.30 | 1.55 | 0.367 | 0.810 |
| rs2286940 | *MLH1* | 1.07 | 0.89 | 1.27 | 0.485 | 0.888 | 1.21 | 0.64 | 2.29 | 0.548 | 0.858 |
| rs2303428 | *MSH2* | 1.14 | 0.86 | 1.51 | 0.377 | 0.806 | 0.96 | 0.38 | 2.44 | 0.253 | 0.757 |
| rs2308321 | *MGMT* | 0.89 | 0.65 | 1.22 | 0.482 | 0.888 | 1.22 | 0.42 | 3.47 | 0.800 | 0.939 |
| rs2345060 | *PMS2* | 0.92 | 0.74 | 1.13 | 0.416 | 0.832 | 0.56 | 0.28 | 1.14 | 0.109 | 0.709 |
| rs2348244 | *MSH6* | 0.81 | 0.61 | 1.07 | 0.145 | 0.624 | 1.47 | 0.53 | 4.13 | 0.629 | 0.913 |
| rs238406 | *ERCC2* | 0.98 | 0.82 | 1.16 | 0.785 | 0.986 | 1.41 | 0.81 | 2.45 | 0.217 | 0.757 |
| rs2434470 | *ALKBH3* | 0.88 | 0.71 | 1.09 | 0.251 | 0.642 | 0.60 | 0.31 | 1.15 | 0.162 | 0.709 |
| rs2440 | *XRCC5* | 1.08 | 0.90 | 1.29 | 0.434 | 0.852 | 1.02 | 0.56 | 1.85 | 0.956 | 1 |
| rs25487 | *XRCC1* | 0.88 | 0.73 | 1.05 | 0.144 | 0.624 | 0.89 | 0.50 | 1.60 | 0.704 | 0.939 |
| rs26279 | *MSH3* | 0.99 | 0.83 | 1.21 | 0.989 | 0.992 | 1.04 | 0.58 | 1.85 | 0.894 | 1 |
| rs26779 | *MSH3* | 0.85 | 0.70 | 1.02 | 0.077 | 0.490 | 0.94 | 0.52 | 1.72 | 0.845 | 1 |
| rs293794 | *OGG1* | 1.01 | 0.80 | 1.28 | 0.925 | 0.992 | 0.83 | 0.37 | 1.86 | 0.648 | 0.922 |
| rs3136038 | *ERCC4* | 0.81 | 0.67 | 0.98 | **0.034** | 0.473 | 0.72 | 0.40 | 1.32 | 0.289 | 0.779 |
| rs3136228 | *MSH6* | 1.09 | 0.90 | 1.33 | 0.358 | 0.805 | 0.80 | 0.44 | 1.44 | 0.454 | 0.840 |
| rs3212948 | *ERCC1* | 1.03 | 0.85 | 1.24 | 0.771 | 0.980 | 0.70 | 0.40 | 1.24 | 0.222 | 0.757 |
| rs3212961 | *ERCC1* | 1.20 | 0.91 | 1.59 | 0.202 | 0.626 | 0.57 | 0.26 | 1.24 | 0.163 | 0.757 |
| rs3212986 | *ERCC1* | 0.98 | 0.79 | 1.22 | 0.869 | 0.992 | 0.76 | 0.39 | 1.46 | 0.409 | 0.819 |
| rs3213245 | *XRCC1* | 1.17 | 0.97 | 1.40 | 0.096 | 0.546 | 1.24 | 0.68 | 2.26 | 0.477 | 0.840 |
| rs3218536 | *XRCC2* | 1.08 | 0.79 | 1.46 | 0.643 | 0.936 | 0.51 | 0.22 | 1.16 | 0.114 | 0.709 |
| rs3219489 | *MUTYH* | 1.06 | 0.86 | 1.31 | 0.592 | 0.936 | 1.11 | 0.87 | 1.41 | 0.392 | 0.814 |
| rs3626 | *PCNA* | 0.93 | 0.71 | 1.23 | 0.628 | 0.936 | 0.70 | 0.32 | 1.53 | 0.227 | 0.757 |
| rs3730668 | *POLI* | 0.88 | 0.72 | 1.06 | 0.169 | 0.626 | 0.73 | 0.42 | 1.25 | 0.242 | 0.757 |
| rs3793784 | *ERCC6* | 0.84 | 0.70 | 1.01 | 0.065 | 0.473 | 1.14 | 0.63 | 2.04 | 0.665 | 0.931 |
| rs4150416 | *ERCC3* | 0.83 | 0.68 | 1.01 | 0.067 | 0.473 | 0.62 | 0.32 | 1.20 | 0.151 | 0.757 |
| rs4150441 | *ERCC3* | 1.04 | 0.87 | 1.25 | 0.662 | 0.936 | 1.66 | 0.86 | 3.19 | 0.125 | 0.709 |
| rs4150474 | *ERCC3* | 0.87 | 0.71 | 1.08 | 0.210 | 0.626 | 0.68 | 0.35 | 1.36 | 0.278 | 0.768 |
| rs4234259 | *MLH1* | 1.05 | 0.88 | 1.25 | 0.618 | 0.936 | 1.30 | 0.70 | 2.43 | 0.405 | 0.819 |
| rs4253160 | *ERCC6* | 0.83 | 0.69 | 0.99 | **0.041** | 0.473 | 0.98 | 0.57 | 1.69 | 0.951 | 1 |
| rs4968451 | *BRIP1* | 0.90 | 0.70 | 1.15 | 0.389 | 0.806 | 0.97 | 0.43 | 2.18 | 0.814 | 1 |
| rs4986764 | *BRIP1* | 0.86 | 0.72 | 1.04 | 0.113 | 0.583 | 1.10 | 0.59 | 2.06 | 0.753 | 0.956 |
| rs4987876 | *ATM* | 1.02 | 0.76 | 1.36 | 0.896 | 0.992 | 0.28 | 0.09 | 0.90 | **0.020** | 0.520 |
| rs569143 | *MRE11A* | 0.84 | 0.70 | 1.01 | 0.059 | 0.473 | 0.82 | 0.46 | 1.46 | 0.507 | 0.840 |
| rs5744934 | *POLE* | 1.15 | 0.91 | 1.45 | 0.249 | 0.642 | 0.98 | 0.43 | 2.25 | 0.969 | 1 |
| rs601341 | *MRE11A* | 0.87 | 0.72 | 1.05 | 0.147 | 0.624 | 1.01 | 0.58 | 1.73 | 0.989 | 1 |
| rs6413436 | *RAD52* | 1.22 | 1.01 | 1.48 | **0.036** | 0.473 | 0.65 | 0.36 | 1.15 | 0.136 | 0.736 |
| rs664143 | *ATM* | 0.83 | 0.69 | 1.01 | 0.055 | 0.473 | 1.39 | 0.77 | 2.49 | 0.266 | 0.757 |
| rs7182283 | *NEIL1* | 0.96 | 0.80 | 1.16 | 0.690 | 0.936 | 0.56 | 0.31 | 1.01 | 0.068 | 0.709 |
| rs735943 | *EXO1* | 0.93 | 0.78 | 1.12 | 0.455 | 0.878 | 1.48 | 0.79 | 2.77 | 0.211 | 0.757 |
| rs7797466 | *PMS2* | 1.07 | 0.85 | 1.36 | 0.560 | 0.936 | 2 | 0.71 | 5.63 | 0.512 | 0.840 |
| rs799917 | *BRCA1* | 1.01 | 0.83 | 1.22 | 0.934 | 0.992 | 1.32 | 0.70 | 2.47 | 0.388 | 0.819 |
| rs8305 | *POLI* | 1.25 | 1.02 | 1.53 | **0.032** | 0.473 | 0.60 | 0.31 | 1.15 | 0.123 | 0.709 |
| rs861528 | *XRCC3* | 1.20 | 0.96 | 1.49 | 0.112 | 0.583 | 1.05 | 0.54 | 2.03 | 0.881 | 1 |
| rs861531 | *XRCC3* | 1.01 | 0.83 | 1.22 | 0.937 | 0.992 | 0.95 | 0.52 | 1.74 | 0.872 | 1 |
| rs861539 | *XRCC3* | 0.95 | 0.79 | 1.15 | 0.622 | 0.936 | 0.86 | 0.47 | 1.56 | 0.619 | 0.913 |
| rs9350 | *EXO1* | 0.99 | 0.78 | 1.28 | 0.992 | 0.992 | 0.32 | 0.14 | 0.78 | **0.014** | 0.520 |
| rs963248 | *XRCC4* | 1.02 | 0.80 | 1.30 | 0.887 | 0.992 | 0.95 | 0.44 | 2.03 | 0.886 | 1 |
| rs9876116 | *MLH1* | 1.02 | 0.85 | 1.22 | 0.843 | 0.992 | 1.17 | 0.63 | 2.18 | 0.624 | 0.913 |
| rs9894946 | *TP53* | 0.77 | 0.56 | 1.04 | 0.092 | 0.546 | 0.83 | 0.61 | 1.13 | 0.229 | 0.757 |

GC, gastric cancer; OR, odds ratio; CI, confidence interval.

^a^ORs adjusted by gender, age, *Helicobacter pylori* infection, and smoking status.

^b^QFDR-values obtained after applying the False Discovery Rate (FDR) test.

*P*-values <0.05 are highlighted in bold.
