# Supplementary material for: Relevance of DNA repair gene polymorphisms to gastric cancer risk and phenotype
Source: Oncotarget. 2017 Mar 16;8(22):35848–62. doi: 10.18632/oncotarget.16261 (PMC5482622; doi:10.18632/oncotarget.16261)
Supplement: Supplementary file 11 [file oncotarget-08-35848-s011.doc]

**Supplementary Table 11: Haplotypes in DNA repair genes and association with GC** risk.

| **Gene** | **SNPs** | **Haplotype** | **Frequency HC** | | **Frequency GC** | **OR (95% CI)*** | | ***P* value** | |  |
| --- | --- | --- | --- | --- | --- | --- | --- | --- | --- | --- |
| *APEX1* | rs1130409 | GC | 0.3739 | | 0.3529 | Reference | |  | |  |
|  | rs1760944 | TA | 0.2579 | | 0.2638 | 1.08 (0.87 - 1.33) | | 0.4743 | |  |
|  |  | TC | 0.2505 | | 0.2703 | 1.14 (0.90 - 1.45) | | 0.2746 | |  |
|  |  | GA | 0.1177 | | 0.1129 | 1.02 (0.72 - 1.43) | | 0.9212 | |  |
| *ATM* | rs1801516 | GGC | 0.3864 | | 0.4109 | | Reference | |  | |
|  | rs4987876 | GGT | 0.3838 | | 0.3544 | | 0.86 (0.71 - 1.03) | | 0.0940 | |
|  | rs664143 | AGC | 0.1364 | | 0.1505 | | 1.04 (0.81 - 1.33) | | 0.7626 | |
|  |  | GTC | 0.0934 | | 0.0842 | | 0.85 (0.64 - 1.13) | | 0.2665 | |
| *BRCA1* | rs1060915 | TAC | 0.6500 | 0.6359 | | | Reference | |  | |
|  | rs1799966 | CGT | 0.3258 | 0.3447 | | | 1.08 (0.91 - 1.29) | | 0.3657 | |
|  | rs799917 |  |  |  | | |  | |  | |
| *BRCA2* | rs144848 | TAA | 0.4131 | 0.4150 | | | Reference | |  | |
|  | rs1799955 | GAA | 0.2768 | 0.2953 | | | 1.06 (0.87-1.29) | | 0.5805 | |
|  | rs1801406 | TGG | 0.2241 | 0.2202 | | | 0.97 (0.78-1.20) | | 0.7520 | |
|  |  | TAG | 0.0846 | 0.0686 | | | 0.80 (0.58-1.11) | | 0.1778 | |
| *ERCC1* | rs3212948 | CCG | 0.6040 | 0.6079 | | | Reference | |  | |
|  | rs3212961 | GCT | 0.2514 | 0.2499 | | | 0.99 (0.82 - 1.19) | | 0.9191 | |
|  | rs3212986 | GAG | 0.0940 | 0.1006 | | | 1.08 (0.82 - 1.42) | | 0.6033 | |
| *ERCC2* | rs13181 | TAGT | 0.4639 | 0.4506 | | | Reference | |  | |
|  | rs1618536 | GGAG | 0.2649 | 0.2698 | | | 1.04 (0.86 - 1.26) | | 0.6610 | |
|  | rs1799793 | TGGG | 0.1573 | 0.1369 | | | 0.91 (0.72 - 1.15) | | 0.4394 | |
|  | rs238406 |  |  |  | | |  | |  | |
| *ERCC3* | rs4150416 | TGT | 0.3970 | 0.4147 | | | Reference | |  | |
|  | rs4150441 | TAT | 0.2784 | 0.2959 | | | 1.02 (0.84 - 1.25) | | 0.8399 | |
|  | rs4150474 | GAG | 0.2587 | 0.2322 | | | 0.86 (0.70 - 1.05) | | 0.1417 | |
|  |  | GAT | 0.0658 | 0.0572 | | | 0.83 (0.58 - 1.18) | | 0.2973 | |
| *ERCC6* | rs3793784 | CA | 0.5372 | 0.5623 | | | Reference | |  | |
|  | rs4253160 | GT | 0.4258 | 0.4049 | | | 0.91 (0.77 - 1.07) | | 0.2543 | |
| *EXO1* | rs1047840 | GGCC | 0.2735 | 0.2780 | | | Reference | |  | |
|  | rs1776148 | GATC | 0.1460 | 0.1418 | | | 0.92 (0.69 - 1.24) | | 0.5839 | |
|  | rs735943 | AATC | 0.1454 | 0.1416 | | | 0.93 (0.70 - 1.24) | | 0.6165 | |
|  | rs9350 | AGTC | 0.0952 | 0.0749 | | | 0.79 (0.55 - 1.14) | | 0.2077 | |
|  |  | GGTC | 0.0671 | 0.0873 | | | 1.18 (0.79 - 1.76) | | 0.4165 | |
|  |  | AGCT | 0.0722 | 0.0660 | | | 0.90 (0.62 - 1.30) | | 0.5658 | |
|  |  | GGCT | 0.0585 | 0.0550 | | | 0.95 (0.63 - 1.45) | | 0.8211 | |
| *LIG4* | rs1805386 | TC | 0.6982 | 0.6891 | | | Reference | |  | |
|  | rs1805388 | CC | 0.1692 | 0.1649 | | | 0.98 (0.79 - 1.23) | | 0.8816 | |
|  |  | TT | 0.1326 | 0.1459 | | | 1.12 (0.87 - 1.42) | | 0.3780 | |
| *MLH1* | rs1540354 | TGGTGG | 0.3350 | 0.3281 | | | Reference | |  | |
|  | rs1799977 | TAACAA | 0.2410 | 0.2429 | | | 1.03 (0.83 - 1.28) | | 0.7602 | |
|  | rs1800734 | TAGCAA | 0.1476 | 0.1505 | | | 1.05 (0.82 - 1.34) | | 0.7130 | |
|  | rs2286940 | TAGTGG | 0.1396 | 0.1403 | | | 1.03 (0.80 - 1.33) | | 0.8309 | |
|  | rs4234259 | AAGCAA | 0.1215 | 0.1085 | | | 0.93 (0.71 - 1.21) | | 0.5832 | |
|  | rs9876116 |  |  |  | | |  | |  | |
| *MRE11A* | rs569143 | CG | 0.4895 | 0.5232 | | | Reference | |  | |
|  | rs601341 | GA | 0.4438 | 0.4108 | | | 0.86 (0.73 - 1.02) | | 0.0873 | |
|  |  | GG | 0.06520 | 0.06490 | | | 0.95 (0.68 - 1.32) | | 0.7410 | |
| *MSH2* | rs1981928 | TT | 0.7227 | 0.7276 | | | Reference | |  | |
|  | rs2303428 | AT | 0.1810 | 0.1627 | | | 0.90 (0.72 - 1.12) | | 0.3330 | |
|  |  | AC | 0.0943 | 0.1096 | | | 1.14 (0.87 - 1.49) | | 0.3343 | |
| *MSH3* | rs10079641 | CCAG | 0.3845 | 0.4021 | | | Reference | |  | |
|  | rs1650697 | CCGG | 0.1773 | 0.1823 | | | 0.98 (0.76 - 1.25) | | 0.8473 | |
|  | rs26279 | CTGA | 0.1341 | 0.1330 | | | 0.94 (0.73 - 1.22) | | 0.6430 | |
|  | rs26779 | CTAA | 0.1134 | 0.0907 | | | 0.76 (0.55 - 1.05) | | 0.1004 | |
|  |  | GCAA | 0.0975 | 0.0903 | | | 0.89 (0.66 - 1.20) | | 0.4304 | |
|  |  | CCAA | 0.0603 | 0.0560 | | | 0.87 (0.59 - 1.28) | | 0.4744 | |
| *MSH6* | rs1800935 | TATT | 0.4574 | 0.4839 | | | Reference | |  | |
|  | rs2020911 | CTTG | 0.1597 | 0.1954 | | | 1.15 (0.92 - 1.43) | | 0.2267 | |
|  | rs2348244 | TTCT | 0.1356 | 0.1132 | | | 0.79 (0.61 - 1.03) | | 0.0762 | |
|  | rs3136228 | CATG | 0.1132 | 0.1046 | | | 0.89 (0.66 - 1.18) | | 0.4166 | |
|  |  | TATG | 0.0701 | 0.0606 | | | 0.84 (0.60 - 1.18) | | 0.3051 | |
| *OGG1* | rs1052133 | CT | 0.6140 | 0.6230 | | | Reference | |  | |
|  | rs293794 | GT | 0.2137 | 0.2023 | | | 0.93 (0.76 - 1.15) | | 0.5062 | |
|  |  | CC | 0.1687 | 0.1705 | | | 0.99 (0.79 - 1.25) | | 0.9558 | |
| *PMS2* | rs2228006 | GAG | 0.4481 | 0.4521 | | | Reference | |  | |
|  | rs2345060 | GGG | 0.2496 | 0.2227 | | | 0.89 (0.72 - 1.09) | | 0.2671 | |
|  | rs7797466 | AAG | 0.1508 | 0.1623 | | | 1.06 (0.83 - 1.35) | | 0.6342 | |
|  |  | GAA | 0.1475 | 0.1629 | | | 1.09 (0.86 - 1.38) | | 0.4831 | |
| *RAD52* | rs11226 | CT | 0.5969 | 0.5477 | | | Reference | |  | |
|  | rs6413436 | TC | 0.3225 | 0.3533 | | | 1.19 (1.00 - 1.41) | | 0.0494 | |
|  |  | TT | 0.0778 | 0.0959 | | | 1.34 (0.99 - 1.81) | | 0.0594 | |
| *WRN* | rs1346044 | TT | 0.6382 | 0.6738 | | | Reference | |  | |
|  | rs1800389 | CC | 0.1345 | 0.1232 | | | 0.87 (0.68 - 1.12) | | 0.2796 | |
|  |  | TC | 0.1331 | 0.1204 | | | 0.87 (0.67 - 1.12) | | 0.2713 | |
|  |  | CT | 0.0942 | 0.0826 | | | 0.83 (0.61 - 1.14) | | 0.2533 | |
| *XPC* | rs2228000 | CC | 0.3958 | 0.3923 | | | Reference | |  | |
|  | rs2228001 | TA | 0.3275 | 0.2844 | | | 0.87 (0.72 - 1.06) | | 0.1658 | |
|  |  | CA | 0.2767 | 0.323 | | | 1.18 (0.97 - 1.45) | | 0.0965 | |
| *XRCC1* | rs25487 | GC | 0.3756 | 0.3842 | | | Reference | |  | |
|  | rs3213245 | AT | 0.3655 | 0.3442 | | | 0.88 (0.74 - 1.06) | | 0.1685 | |
|  |  | GT | 0.2406 | 0.2526 | | | 1.00 (0.81 - 1.24) | | 0.9756 | |
| *XRCC2* | rs2040639 | AG | 0.4649 | 0.4347 | | | Reference | |  | |
|  | rs3218536 | GG | 0.4363 | 0.4616 | | | 1.13 (0.95 - 1.33) | | 0.1605 | |
|  |  | GA | 0.0988 | 0.1036 | | | 1.13 (0.85 - 1.50) | | 0.4128 | |
| *XRCC3* | rs1799794 | GAGGC | 0.2575 | 0.2444 | | | Reference | |  | |
|  | rs1799796 | AAATT | 0.2273 | 0.2469 | | | 1.15 (0.91 - 1.45) | | 0.2452 | |
|  | rs861528 | AGGGC | 0.2428 | 0.2237 | | | 0.96 (0.76 - 1.21) | | 0.7108 | |
|  | rs861531 | AAGTT | 0.1466 | 0.1320 | | | 0.94 (0.72 - 1.24) | | 0.6761 | |
|  | rs861539 | AAGGC | 0.1173 | 0.1316 | | | 1.16 (0.88 - 1.52) | | 0.2948 | |
| *XRCC4* | rs13180316 | GTTA | 0.3073 | 0.31894 | | | Reference | |  | |
|  | rs1478485 | ACGA | 0.2521 | 0.22915 | | | 0.90 (0.72 - 1.12) | | 0.3596 | |
|  | rs2075685 | GCGA | 0.1730 | 0.1677 | | | 0.95 (0.73 - 1.23) | | 0.7048 | |
|  | rs963248 | GCGG | 0.1111 | 0.12020 | | | 1.00 (0.75 - 1.34) | | 0.9823 | |
| *XRCC5* | rs1051677 | TAGT | 0.4413 | 0.4477 | | | Reference | |  | |
|  | rs1051685 | TAGC | 0.3271 | 0.3142 | | | 0.94 (0.78 - 1.14) | | 0.5551 | |
|  | rs207906 | CAGC | 0.0882 | 0.0970 | | | 1.09 (0.81 - 1.46) | | 0.5885 | |
|  | rs2440 | TGAC | 0.0797 | 0.0771 | | | 0.95 (0.70 - 1.30) | | 0.7626 | |

GC, gastric cancer; OR, odds ratio; CI, confidence interval.

Haplotypes with frequencies > 0.05 are shown in the table.

Odds ratios and 95% confidence intervals were calculated taking as a reference the more common haplotype.
