# Supplementary material for: Relevance of DNA repair gene polymorphisms to gastric cancer risk and phenotype
Source: Oncotarget. 2017 Mar 16;8(22):35848–62. doi: 10.18632/oncotarget.16261 (PMC5482622; doi:10.18632/oncotarget.16261)
Supplement: Supplementary file 12 [file oncotarget-08-35848-s012.doc]

**Supplementary Table 12: Characteristics of candidate SNPs analy**zed in the study.

| **SNP^a^** | **Pathway^b^** | **Gene** | **Chr** | **Position^c^** | **SNP Type** | **Alleles^d^** | **MAF** | **HWE^e^** | **Total N (%)** |
| --- | --- | --- | --- | --- | --- | --- | --- | --- | --- |
| rs3219489 | MMR | *MUTYH* | 1 | 45331833 | Q324H | G/C | 0.27 | 0.542 | 1192 (98.8) |
| rs3219484 | MMR | *MUTYH* | 1 | 45334484 | V22M | G/A | NA | NA | ― |
| rs1048771 | HR | *RAD54L* | 1 | 46278228 | A730A | C/T | 0.12 | 0.412 | 1126 (93.4) |
| rs1136410 | BER | *PARP1* | 1 | 226367601 | V762A | T/C | 0.13 | 0.263 | 1206 (100) |
| rs735943 | MMR | *EXO1* | 1 | 241866849 | H354R | C/T | 0.47 | 0.086 | 1201 (99.6) |
| rs1047840 | MMR | *EXO1* | 1 | 241878999 | E589K | G/A | 0.39 | 0.932 | 1190 (98.7) |
| rs1776148 | MMR | *EXO1* | 1 | 241879243 | E670G | G/A | 0.39 | 0.863 | 1200 (99.5) |
| rs9350 | MMR | *EXO1* | 1 | 241885372 | P757L | C/T | 0.15 | 0.215 | 1195 (99.1) |
| rs1863332 | MMR | *MSH2* | 2 | 47402759 | Upstream | A/C | 0.08 | 0.032 | 1027 (85.2) |
| rs2303426 | MMR | *MSH2* | 2 | 47403411 | Intronic | G/C | NA | NA | ― |
| rs1981928 | MMR | *MSH2* | 2 | 47445336 | Intronic | T/A | 0.27 | 1.000 | 1200 (99.5) |
| rs2303428 | MMR | *MSH2* | 2 | 47476361 | Intronic | T/C | 0.10 | 0.153 | 1204 (99.8) |
| rs3136228 | MMR | *MSH6* | 2 | 47782677 | Upstream | T/G | 0.35 | 0.717 | 1197 (99.3) |
| rs2348244 | MMR | *MSH6* | 2 | 47792346 | Intronic | T/C | 0.13 | 1.000 | 1205 (99.9) |
| rs1800935 | MMR | *MSH6* | 2 | 47795976 | D180D | T/C | 0.29 | 0.682 | 1197 (99.3) |
| rs2020911 | MMR | *MSH6* | 2 | 47803699 | Intronic | A/T | 0.35 | 0.533 | 1204 (99.8) |
| rs4150474 | NER | *ERCC3* | 2 | 127275751 | Intronic | T/G | 0.24 | 0.199 | 1203 (99.8) |
| rs4150441 | NER | *ERCC3* | 2 | 127283339 | Intronic | A/G | 0.41 | 0.733 | 1199 (99.4) |
| rs4150416 | NER | *ERCC3* | 2 | 127288972 | Intronic | T/G | 0.31 | 0.457 | 1204 (99.8) |
| rs207906 | NHEJ | *XRCC5* | 2 | 216148178 | T524T | G/A | 0.12 | 0.351 | 1205 (99.9) |
| rs1051677 | NHEJ | *XRCC5* | 2 | 216205525 | 3´ UTR | T/C | 0.09 | 1.000 | 1204 (99.8) |
| rs1051685 | NHEJ | *XRCC5* | 2 | 216205653 | 3´ UTR | A/G | 0.10 | 0.062 | 1204 (99.8) |
| rs2440 | NHEJ | *XRCC5* | 2 | 216206043 | 3´ UTR | C/T | 0.45 | 0.804 | 1203 (99.8) |
| rs1052133 | BER | *OGG1* | 3 | 9757089 | S326C | C/G | 0.21 | 0.632 | 1206 (100) |
| rs293794 | BER | *OGG1* | 3 | 9761943 | Intronic | T/C | 0.17 | 0.670 | 1198 (99.3) |
| rs2228001 | NER | *XPC* | 3 | 14145949 | K939Q | A/C | 0.39 | 0.865 | 1128 (93.5) |
| rs2228000 | NER | *XPC* | 3 | 14158387 | A499V | C/T | 0.31 | 0.403 | 1198 (99.3) |
| rs1800734 | MMR | *MLH1* | 3 | 36993455 | 5´ UTR | G/A | 0.24 | 0.267 | 1195 (99.1) |
| rs1540354 | MMR | *MLH1* | 3 | 37002998 | Intronic | T/A | 0.12 | 0.124 | 1202 (99.7) |
| rs4234259 | MMR | *MLH1* | 3 | 37007142 | Intronic | A/G | 0.49 | 0.101 | 1197 (99.3) |
| rs1799977 | MMR | *MLH1* | 3 | 37012077 | I219V | A/G | 0.33 | 0.360 | 1206 (100) |
| rs2286940 | MMR | *MLH1* | 3 | 37028615 | Intronic | C/T | 0.49 | 0.120 | 1200 (99.5) |
| rs9876116 | MMR | *MLH1* | 3 | 37042249 | Intronic | A/G | 0.48 | 0.087 | 1201 (99.6) |
| rs1802904 | CCC | *ATR* | 3 | 142449489 | Q2625Q | A/G | 0.13 | 0.264 | 1202 (99.7) |
| rs2227928 | CCC | *ATR* | 3 | 142562770 | M211T | C/T | NA | NA | ― |
| rs1650697 | MMR | *MSH3* | 5 | 80654962 | V79T | C/T | 0.25 | 0.458 | 1116 (92.5) |
| rs26779 | MMR | *MSH3* | 5 | 80763384 | Intronic | G/A | 0.39 | 0.398 | 1201 (99.6) |
| rs10079641 | MMR | *MSH3* | 5 | 80798145 | Intronic | C/G | 0.10 | 0.381 | 1182 (98.0) |
| rs184967 | MMR | *MSH3* | 5 | 80854162 | Q949R | G/A | NA | NA | ― |
| rs26279 | MMR | *MSH3* | 5 | 80873118 | A1045T | A/G | 0.32 | 0.110 | 1203 (99.8) |
| rs2075685 | NHEJ | *XRCC4* | 5 | 83076846 | Upstream | G/T | 0.44 | 1.000 | 1204 (99.8) |
| rs1478485 | NHEJ | *XRCC4* | 5 | 83084708 | Intronic | C/T | 0.41 | 0.865 | 1203 (99.8) |
| rs13180316 | NHEJ | *XRCC4* | 5 | 83163634 | Intronic | G/A | 0.27 | 1.000 | 1194 (99.0) |
| rs963248 | NHEJ | *XRCC4* | 5 | 83238075 | Intronic | A/G | 0.16 | 1.000 | 1205 (99.9) |
| rs2252775 | NHEJ | *RAD50* | 5 | 132582752 | Intronic | A/C | 0.21 | 0.062 | 1202 (99.7) |
| rs2228006 | MMR | *PMS2* | 7 | 5987144 | K541E | G/A | 0.16 | 0.053 | 1126 (93.4) |
| rs2345060 | MMR | *PMS2* | 7 | 5999498 | Intronic | A/G | 0.24 | 1.000 | 1203 (99.8) |
| rs7797466 | MMR | *PMS2* | 7 | 6007152 | Intronic | G/A | 0.16 | 0.056 | 1203 (99.8) |
| rs3218536 | HR | *XRCC2* | 7 | 152648922 | R188H | G/A | 0.10 | 0.253 | 1205 (99.9) |
| rs2040639 | HR | *XRCC2* | 7 | 152678103 | Upstream | G/A | 0.45 | 0.566 | 1194 (99.0) |
| rs1800389 | NHEJ | *WRN* | 8 | 31067041 | C171C | T/C | 0.26 | 0.214 | 1199 (99.4) |
| rs1346044 | NHEJ | *WRN* | 8 | 31167138 | C1367R | T/C | 0.22 | 0.563 | 1199 (99.4) |
| rs12678588 | BER | *POLB* | 8 | 42355555 | Q137R | G | 0.00 | NA | 1206 (100) |
| rs2272615 | BER | *POLB* | 8 | 42361530 | Downstream | A/G | 0.12 | 0.449 | 1190 (98.7) |
| rs1805794 | NHEJ | *NBS1* | 8 | 89978251 | E185Q | C/G | 0.30 | 0.138 | 1204 (99.8) |
| rs1800975 | NER | *XPA* | 9 | 97697296 | 5´ UTR | G/A | 0.31 | 0.637 | 1197 (99.3) |
| rs2228526 | NER | *ERCC6* | 10 | 49470671 | M1097V | A/G | NA | NA | ― |
| rs4253160 | NER | *ERCC6* | 10 | 49485920 | Intronic | A/T | 0.45 | 0.461 | 1201 (99.6) |
| rs2228524 | NER | *ERCC6* | 10 | 49532830 | L45L | G/C | 0.26 | 0.437 | 1066 (88.4) |
| rs3793784 | NER | *ERCC6* | 10 | 49539493 | 5´ UTR | C/G | 0.42 | 0.319 | 1199 (99.4) |
| rs12917 | DR | *MGMT* | 10 | 129708019 | L84F | C/T | 0.14 | 0.865 | 1065 (88.3) |
| rs2308321 | DR | *MGMT* | 10 | 129766800 | I174V | A/G | 0.09 | 0.014 | 1144 (94.9) |
| rs2434470 | DR | *ALKBH3* | 11 | 43919052 | E228D | C/G | 0.23 | 0.911 | 1198 (99.3) |
| rs174538 | BER | *FEN1* | 11 | 61792609 | Upstream | G/A | 0.30 | 0.558 | 1112 (92.2) |
| rs601341 | HR | *MRE11A* | 11 | 94434611 | Intronic | G/A | 0.43 | 0.408 | 1198 (99.3) |
| rs569143 | HR | *MRE11A* | 11 | 94455221 | Intronic | C/G | 0.49 | 0.807 | 1199 (99.4) |
| rs4987876 | CCC | *ATM* | 11 | 108221910 | Intronic | G/T | 0.09 | 0.085 | 1201 (99.6) |
| rs1801516 | CCC | *ATM* | 11 | 108304735 | D1853N | G/A | 0.14 | 0.602 | 1204 (99.8) |
| rs664143 | CCC | *ATM* | 11 | 108354934 | Intronic | C/T | 0.37 | 0.932 | 1204 (99.8) |
| rs11226 | HR | *RAD52* | 12 | 912647 | 3´ UTR | C/T | 0.43 | 0.864 | 1118 (92.7) |
| rs6413436 | HR | *RAD52* | 12 | 913513 | Intronic | T/C | 0.34 | 0.926 | 1203 (99.8) |
| rs1059262 | DR | *ALKBH2* | 12 | 109087930 | 3´ UTR | T/G | 0.18 | 0.789 | 1203 (99.8) |
| rs5744934 | NER | *POLE* | 12 | 132643940 | S1396N | A/G | 0.17 | 1.000 | 1200 (99.5) |
| rs5744751 | NER | *POLE* | 12 | 132677409 | A252V | C/T | NA | NA | ― |
| rs144848 | HR | *BRCA2* | 13 | 32332592 | N372H | T/G | 0.29 | 0.154 | 1201 (99.6) |
| rs1801406 | HR | *BRCA2* | 13 | 32337751 | K1132K | A/G | 0.30 | 0.634 | 1202 (99.7) |
| rs1799955 | HR | *BRCA2* | 13 | 32355095 | S2414S | A/G | 0.22 | 0.484 | 1205 (99.9) |
| rs1047768 | NER | *ERCC5* | 13 | 102852167 | H46H | C/T | 0.42 | 0.452 | 1204 (99.8) |
| rs17655 | NER | *ERCC5* | 13 | 102875652 | D1104H | C/G | 0.26 | 0.540 | 1201 (99.6) |
| rs1805386 | NHEJ | *LIG4* | 13 | 108209565 | D568D | T/C | 0.17 | 0.662 | 1200 (99.5) |
| rs1805388 | NHEJ | *LIG4* | 13 | 108211243 | T9I | C/T | 0.14 | 1.000 | 1113 (92.3) |
| rs1760944 | BER | *APEX1* | 14 | 20454990 | Upstream | C/A | 0.38 | 0.364 | 1090 (90.4) |
| rs1130409 | BER | *APEX1* | 14 | 20456995 | D148E | T/G | 0.48 | 1.000 | 1201 (99.6) |
| rs10483813 | HR | *RAD51B* | 14 | 68564567 | Intronic | T/A | NA | NA | ― |
| rs175080 | MMR | *MLH3* | 14 | 75047125 | L844P | G/A | 0.44 | 0.280 | 1194 (99.0) |
| rs861539 | HR | *XRCC3* | 14 | 103699416 | T241M | C/T | 0.37 | 0.603 | 1206 (100) |
| rs1799796 | HR | *XRCC3* | 14 | 103699590 | Intronic | A/G | 0.23 | 0.375 | 1202 (99.7) |
| rs861531 | HR | *XRCC3* | 14 | 103706470 | Intronic | G/T | 0.39 | 0.794 | 1192 (98.8) |
| rs1799794 | HR | *XRCC3* | 14 | 103712930 | 5´ UTR | A/G | 0.25 | 0.669 | 1201 (99.6) |
| rs861528 | HR | *XRCC3* | 14 | 103716661 | Intronic | G/A | 0.25 | 0.727 | 1116 (92.5) |
| rs7182283 | BER | *NEIL1* | 15 | 75351418 | Intronic | G/T | 0.47 | 0.163 | 1191 (98.8) |
| rs176641 | BER | *POLG* | 15 | 89346951 | Upstream | A/C | 0.36 | 0.589 | 1205 (99.9) |
| rs3136038 | NER | *ERCC4* | 16 | 13919522 | Upstream | C/T | 0.36 | 0.262 | 1202 (99.7) |
| rs2238463 | NER | *ERCC4* | 16 | 13924045 | Intronic | C/G | 0.38 | 0.269 | 1203 (99.8) |
| rs1800067 | NER | *ERCC4* | 16 | 13935176 | R415Q | G/A | 0.12 | 0.698 | 1189 (98.6) |
| rs1799801 | NER | *ERCC4* | 16 | 13948101 | S835S | T/C | 0.30 | 0.925 | 1202 (99.7) |
| rs9894946 | CCC | *TP53* | 17 | 7667762 | Intronic | G/A | 0.15 | 0.771 | 1188 (98.5) |
| rs1614984 | CCC | *TP53* | 17 | 7668134 | Downstream | C/T | 0.40 | 0.727 | 1113 (92.3) |
| rs1042522 | CCC | *TP53* | 17 | 7676154 | P72R | G/C | 0.25 | 0.41 | 1204 (99.8) |
| rs2074522 | BER | *LIG3* | 17 | 35002629 | Intronic | G/A | 0.08 | 0.565 | 1203 (99.8) |
| rs1799966 | HR | *BRCA1* | 17 | 43071077 | S1613G | A/G | 0.34 | 0.784 | 1198 (99.3) |
| rs3737559 | HR | *BRCA1* | 17 | 43082287 | Intronic | G/A | 0.07 | 1.000 | 988 (81.9) |
| rs1060915 | HR | *BRCA1* | 17 | 43082453 | S1436S | T/C | 0.34 | 0.781 | 1203 (99.8) |
| rs799917 | HR | *BRCA1* | 17 | 43092919 | P871L | C/T | 0.36 | 0.529 | 1200 (99.5) |
| rs4986764 | HR | *BRIP1* | 17 | 61685986 | S919P | C/T | 0.38 | 0.107 | 1199 (99.4) |
| rs4968451 | HR | *BRIP1* | 17 | 61849946 | Intronic | A/C | 0.17 | 0.778 | 1201 (99.6) |
| rs2048718 | HR | *BRIP1* | 17 | 61863458 | Upstream | C/T | 0.42 | 1.000 | 1159 (96.1) |
| rs3730668 | BER | *POLI* | 18 | 54269477 | Upstream | G/T | 0.42 | 0.080 | 1192 (98.8) |
| rs8305 | BER | *POLI* | 18 | 54294435 | A731T | A/G | 0.27 | 0.672 | 1206 (100) |
| rs25487 | BER | *XRCC1* | 19 | 43551574 | Q399R | G/A | 0.37 | 0.147 | 1206 (100) |
| rs25489 | BER | *XRCC1* | 19 | 43552260 | R280H | G/A | NA | NA | ― |
| rs3213245 | BER | *XRCC1* | 19 | 43575535 | 5´ UTR | T/C | 0.39 | 0.795 | 1203 (99.8) |
| rs13181 | NER | *ERCC2* | 19 | 45351661 | K751Q | T/G | 0.34 | 0.200 | 1205 (99.9) |
| rs1799793 | NER | *ERCC2* | 19 | 45364001 | D312N | G/A | 0.32 | 0.445 | 1205 (99.9) |
| rs238406 | NER | *ERCC2* | 19 | 45365051 | R156R | G/T | 0.50 | 0.121 | 1206 (100) |
| rs1618536 | NER | *ERCC2* | 19 | 45368348 | Intronic | G/A | 0.48 | 0.031 | 1105 (91.6) |
| rs3212986 | NER | *ERCC1* | 19 | 45409478 | Q504K | G/T | 0.26 | 0.524 | 1126 (93.4) |
| rs3212961 | NER | *ERCC1* | 19 | 45419065 | Intronic | C/A | 0.12 | 0.550 | 1205 (99.9) |
| rs11615 | NER | *ERCC1* | 19 | 45420395 | N118N | T/C | NA | NA | ― |
| rs3212948 | NER | *ERCC1* | 19 | 45421104 | Intron 3 | C/G | 0.37 | 0.136 | 1197 (99.3) |
| rs20580 | BER | *LIG1* | 19 | 48151296 | A170A | C/A | 0.49 | 0.287 | 1200 (99.5) |
| rs3626 | BER | *PCNA* | 20 | 5115125 | 3´ UTR | G/C | 0.13 | 0.593 | 1205 (99.9) |
| rs132788 | NHEJ | *XRCC6* | 22 | 41663764 | G593G | G/T | NA | NA | ― |

Chr, chromosome; MAF, minor allele frequency; N, number of individuals successfully genotyped.

NA, not applied.

^a^SNP identification according to the NCBI data base.

^b^DNA repair pathway. BER: base excision repair; NER: nucleotide excision repair; MMR: mismatch repair; HR: repair of DNA double-strand breaks by homologous recombination; NHEJ: repair of DNA double-strand breaks by non homologous end joining; DR: direct repair; CCC: cell cycle checkpoint signalling pathway.

^c^Chromosome position according to the Genome Reference Consortium Human Build 38.p2 (GRCh38.p2).

^d^Major/minor alleles.

^e^Hardy–Weinberg equilibrium *P* values in control population.
